# Supplementary material for: Disparate macrophage responses are linked to infection outcome of Hantan virus in humans or rodents
Source: Nat Commun. 2024 Jan 10;15:438. doi: 10.1038/s41467-024-44687-4 (PMC10781751; doi:10.1038/s41467-024-44687-4)
Supplement: Supplementary file 1 — Supplementary Information [file 41467_2024_44687_MOESM1_ESM.pdf]

Supplementary Information

Title: Disparate Macrophage Responses Determine Infection Outcome of Hantan Virus in Humans or Rodents

Hongwei Ma[1,2,6], Yongheng Yang[1,6], Tiejian Nie[3,6], Rong Yan[1], Yue Si[1], Jing Wei[1,4], Mengyun Li[1], He Liu[1], Wei Ye[1], Hui Zhang[1], Linfeng Cheng[1], Liang Zhang[1], Xin Lv[1], Limin Luo[5], Zhikai Xu[1,*], Xijing Zhang[2,*], Yingfeng Lei[1,*], Fanglin Zhang[1,*]

[1] Department of Microbiology & Pathogen Biology, School of Basic Medical Sciences, Air Force Medical University (the Fourth Military Medical University), Xi'an, Shaanxi 710032, China.
[2] Department of Anaesthesiology & Critical Care Medicine, Xijing Hospital, Air Force Medical University (the Fourth Military Medical University), Xi'an, Shaanxi 710032, China.
[3] Department of Experimental Surgery, Tangdu Hospital, Air Force Medical University (the Fourth Military Medical University), Xi'an, Shaanxi 710038, China.
[4] Shaanxi Provincial Centre for Disease Control and Prevention, Xi'an, Shaanxi 710054, China.
[5] Department of Infectious Disease, Air Force Hospital of Southern Theatre Command, Guangzhou, Guangdong 510602, China.
[6] These authors contributed equally.

[*] Correspondence:
flzhang@fmmu.edu.cn (Fanglin Zhang),
yflei@fmmu.edu.cn (Yingfeng Lei),
zhangxj918@163.com (Xijing Zhang),
zhikaixu@fmmu.edu.cn (Zhikai Xu).

Contents

Suppl. Fig.1 (Related to Fig.1)	3
Suppl. Fig.2 (Related to Fig.1)	4
Suppl. Fig.3 (Related to Fig.2)	5
Suppl. Fig.4 (Related to Fig.2)	6
Suppl. Fig.5 (Related to Fig.2)	8
Suppl. Fig.6 (Related to Fig.3)	9
Suppl. Fig.7 (Related to Fig.3)	11
Suppl. Fig.8 (Related to Fig.4)	13
Suppl. Fig.9 (Related to Fig.4)	15
Suppl. Fig.10 (Related to Fig.4)	16
Suppl. Fig.11 (Related to Fig.4)	18
Suppl. Fig.12 (Related to Fig.5)	20
Suppl. Fig.13 (Related to Fig.6)	21
Suppl. Fig.14 (Related to Fig.6)	23
Suppl. Fig.15 (Related to Fig.6)	24
Suppl. Fig.16 (Related to Fig.7)	26
Suppl. Fig.17 (Related to Fig.7)	28
Suppl. Fig.18 (Related to Fig.8)	29
Suppl. Fig.19 (Related to Fig.8)	31
Suppl. Fig.20 (Related to Fig.8)	33
Suppl. Fig.21	34
Suppl. Fig.22	36
Suppl. Fig.23 (Related to Fig.9)	38
Suppl. Fig.24	39
Suppl. Table 1 (Related to Fig.2)	41
Suppl. Table 2	42
Suppl. Table 3	43
Suppl. Table 4	47

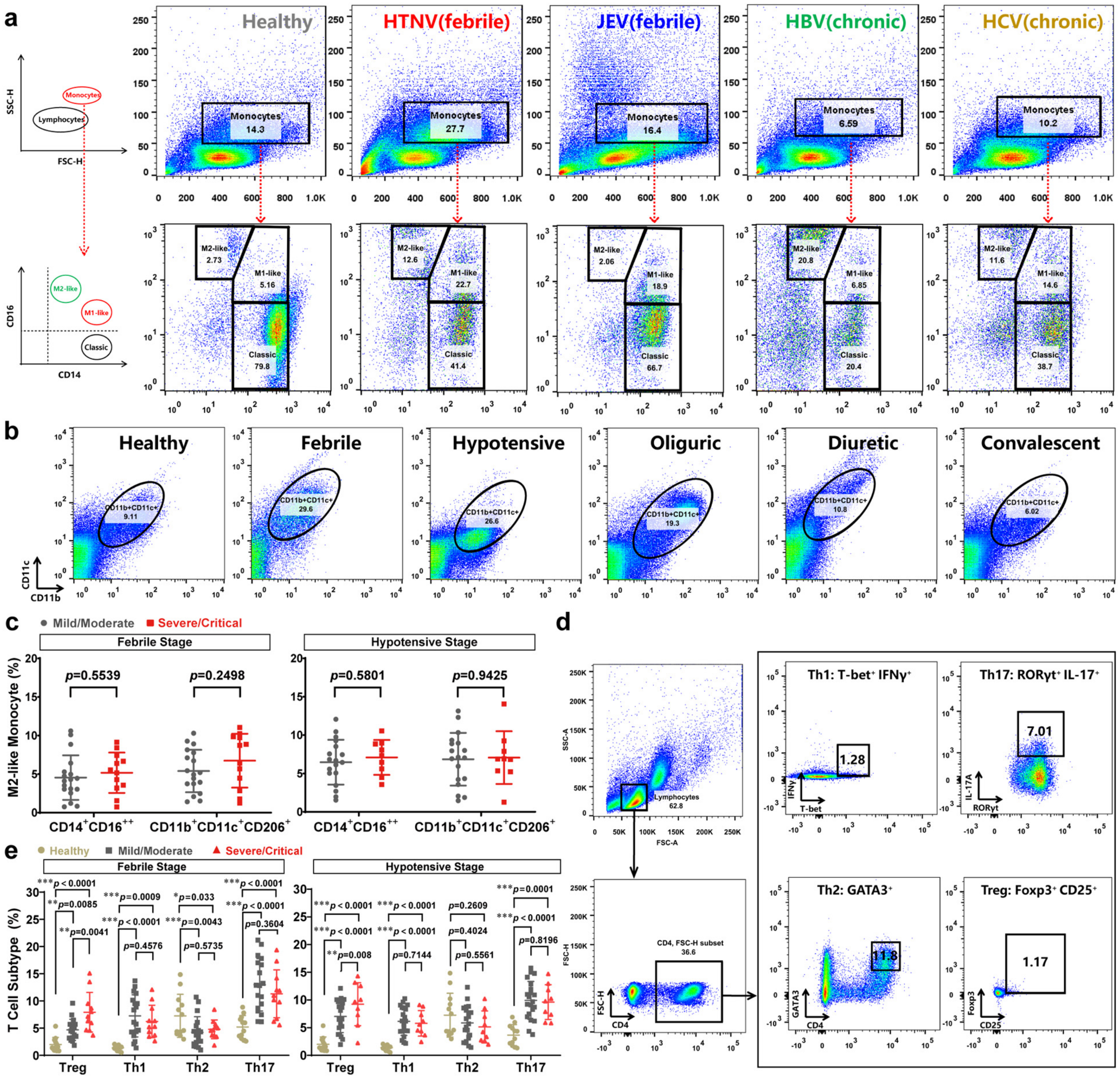

Suppl. Fig.1: Monocyte and T-Cell Subsets in HFRS Patients, Related to Fig.1

(**a**) Gating strategy to identify monocyte subtypes with CD14 and CD16.

(**b**) Gating strategy to measure monocyte percentage with CD11b and CD11c.

(**c**) The relationship between the M2-like monocyte percentage and HFRS severity. For febrile stage, mild/ moderate, n=18, severe/critical, n=12. For hypotensive stage, mild/moderate, n=18, severe/critical, n=9.

(**d**) Gating strategy to distinguish T-cell subsets through flow cytometry with different markers.

(**e**) The T-cell subset percentage of HFRS patients with varying disease severity at the acute infection stage. Gating strategy is shown in Suppl. Fig.1d. For febrile stage, healthy, n=12; mild/moderate, n=18, severe/critical, n=12. For hypotensive stage, healthy, n=12; mild/moderate, n=21, severe/critical, n=9. Data are shown as the mean ± SD. Analysis is performed with two-sided unpaired Student's t test (**c**), or one-way ANOVA (**d**, Dunnett's multiple comparisons test). The exact p values and sample numbers are shown in the figure or figure legend. * $p < 0.05$, ** $p < 0.01$, *** $p < 0.001$. Source data are provided as a Source Data file.

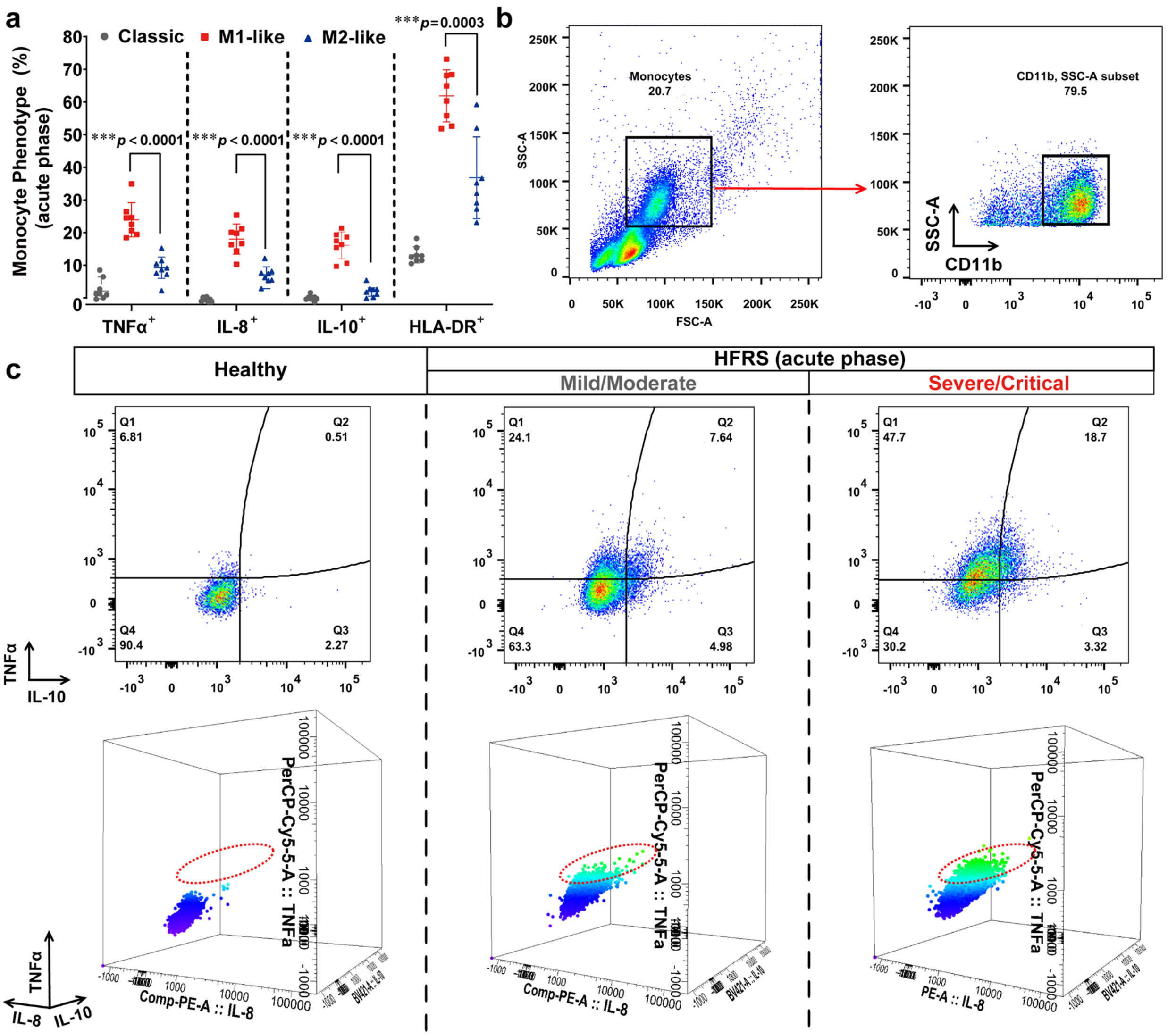

Suppl. Fig.2: Monocyte Phenotype of HFRS Patients, Related to Fig.1

(**a**) The cytokine secretion and HLA-DR expression of monocytes measured by flow cytometry from HFRS patients at the acute stage (n=8).

(**b**) Gating strategy for monocytes ($CD11b^+$).

(**c**) Representative flow cytometric images to evaluate the cytokine secretion pattern of monocytes at the acute infection stage. The gating strategy for $CD11b^+$ monocytes is shown in Suppl. Fig.2b.

Data are shown as the mean ± SD. Analysis is performed with two-sided unpaired Student's t (**a**). The exact p values and sample numbers are shown in the figure. * $p < 0.05$, ** $p < 0.01$, *** $p < 0.001$. Source data are provided as a Source Data file.

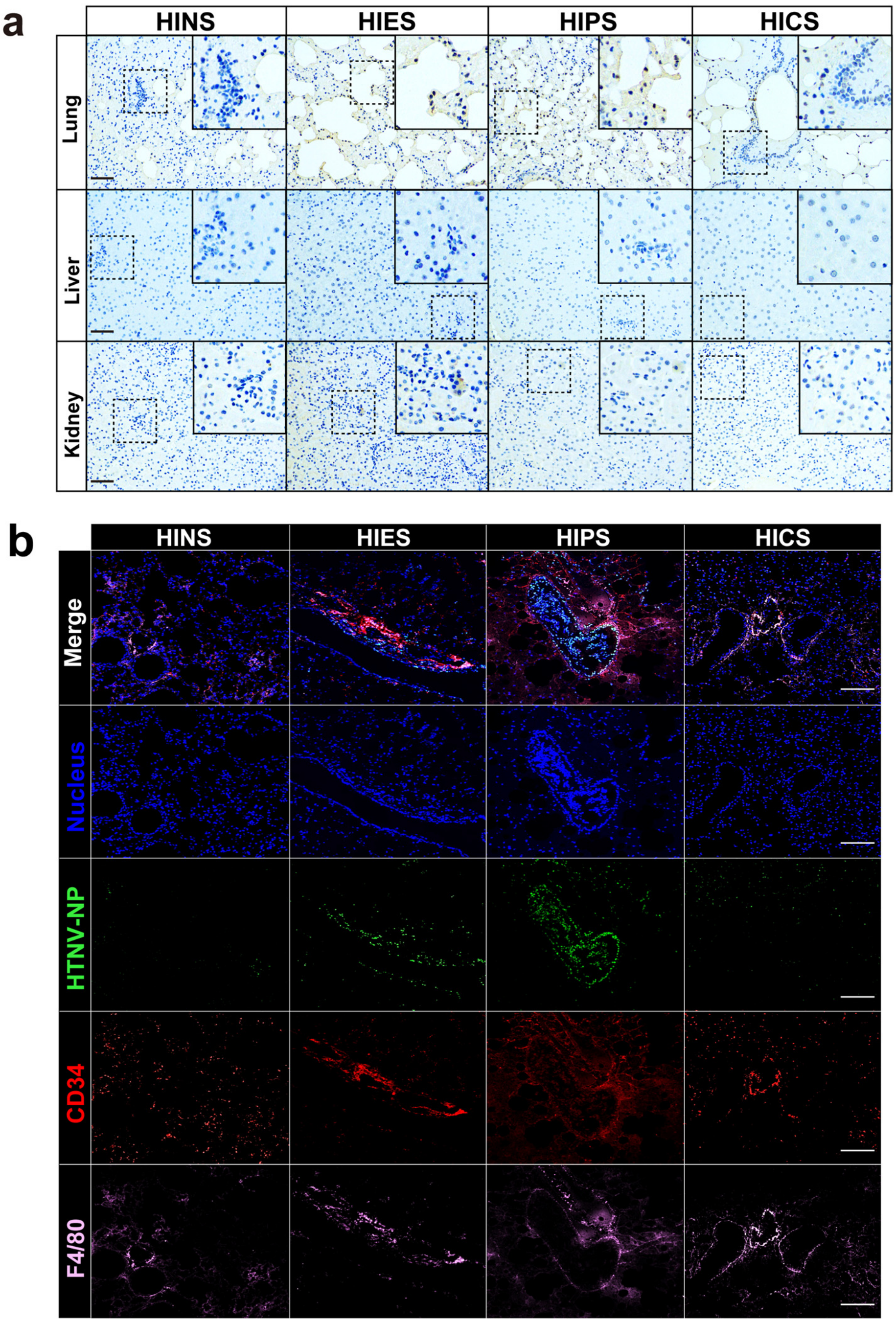

Suppl. Fig.3: Immunopathogenic Features of HTNV Infection in Rodents, Related to Fig.2

(**a**) Representative immunohistochemistry analysis of HTNV NP expression from *A. agrarius* mice of three independent experiments (n=4 for each test). Scale bars, 100 μm.

(**b**) Representative immunohistochemistry analysis of AMs (F4/80^{+}) distribution in the lung tissue of three independent experiments (n=4 for each test). Scale bars, 200 μm.

59

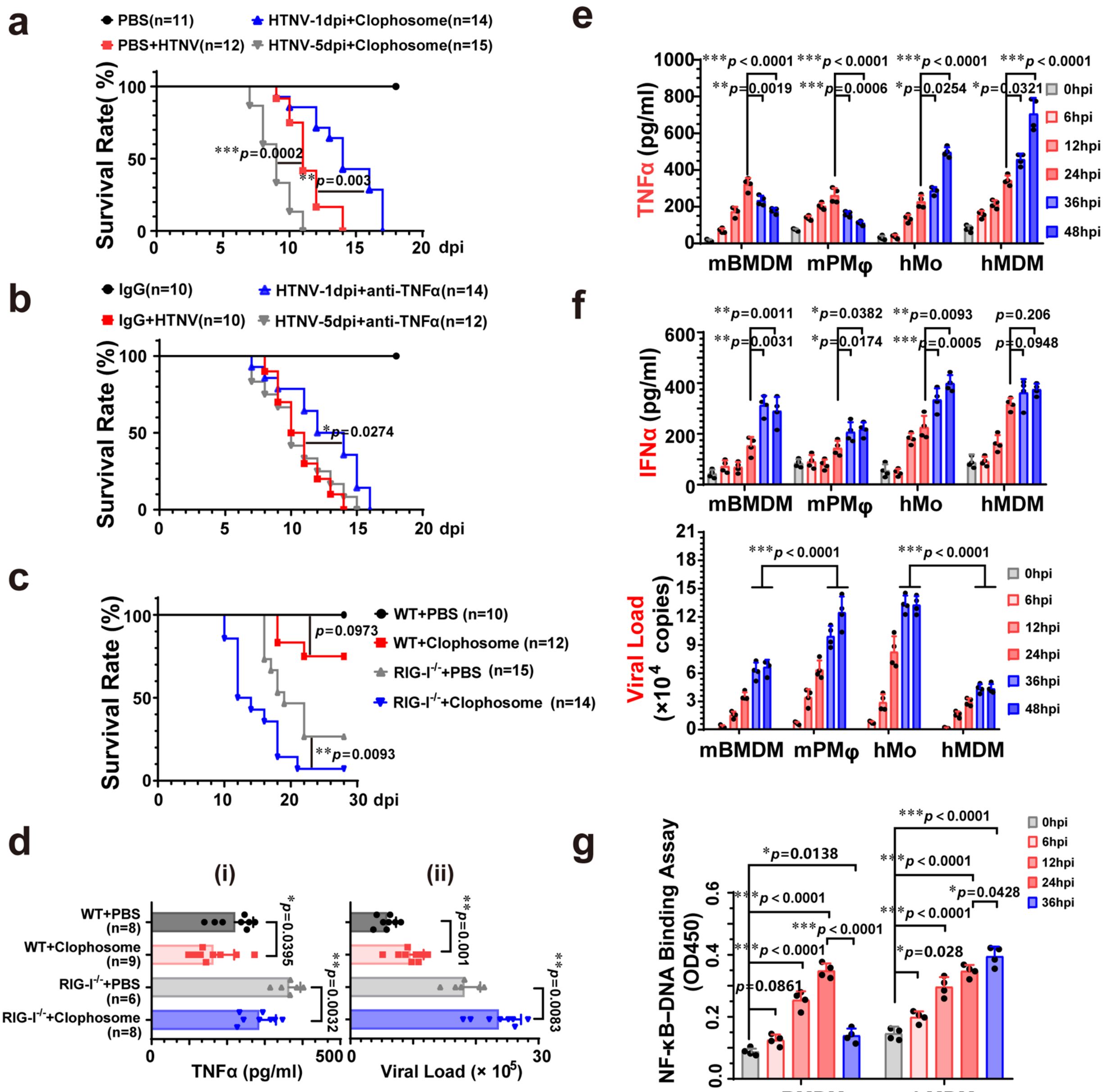
a
PBS(n=11)
HTNV-1dpi+Clophosome(n=14)
PBS+HTNV(n=12)
HTNV-5dpi+Clophosome(n=15)
Survival Rate(%)
***p=0.0002
**p=0.003
dpi
b
IgG(n=10)
HTNV-1dpi+anti-TNFα(n=14)
IgG+HTNV(n=10)
HTNV-5dpi+anti-TNFα(n=12)
Survival Rate (%)
*p=0.0274
dpi
c
Survival Rate (%)
p=0.0973
**p=0.0093
WT+PBS (n=10)
WT+Clophosome (n=12)
RIG-I-/-+PBS (n=15)
RIG-I-/-+Clophosome (n=14)
dpi
d
(i)
(ii)
WT+PBS (n=8)
WT+Clophosome (n=9)
RIG-I-/-+PBS (n=6)
RIG-I-/-+Clophosome (n=8)
*p=0.0395
**p=0.0032
**p=0.001
**p=0.0083
TNFα (pg/ml)
Viral Load (× 10^5)
e
TNFα (pg/ml)
***p < 0.0001
**p=0.0019
***p=0.0006
*p=0.0254
*p=0.0321
0hpi
6hpi
12hpi
24hpi
36hpi
48hpi
mBMDM
mPMφ
hMo
hMDM
f
IFNα (pg/ml)
**p=0.0011
**p=0.0031
*p=0.0382
*p=0.0174
**p=0.0093
***p=0.0005
p=0.206
p=0.0948
Viral Load (×10^4 copies)
***p < 0.0001
g
NF-κB-DNA Binding Assay (OD450)
*p=0.0138
***p < 0.0001
p=0.0861
*p=0.0428
*p=0.028

Suppl. Fig.4: Inflammatory Macrophage Activation Contributes to the Pathogenesis of HTNV Infection, Related to Fig.2

(**a** and **b**) Survival curve of 4-day-old neonatal wild-type (WT) mice (HTNV challenge, 8×10^5 $TCID_{50}$/g). Mice are treated with clophosome (10 μl/g) (**a**), or neutralizing antibodies against TNFα (5 μg/g) (**b**).

(**c**) Survival curve of 6- to 8-week-old adult WT or RIG-I$^{-/-}$ mice pretreated with isotype control (PBS) or clophosome (HTNV challenge, 8×10^5 $TCID_{50}$/g).

(**d**) The serum TNFα concentration (ELISA) (i) and viral load (qRT-PCR) (ii) at 3 dpi from (**c**).

(**e**) Supernatant cytokine concentration of TNFα assessed by ELISA (MOI=1, n=4), related to Fig.2d.

(**f**) Supernatant cytokine concentration of IFNα (ELISA) and the viral loads of HTNV (qPCR) (MOI=1, n=4), related to Fig.2d.

(**g**) The DNA binding capacity of p65 from 0 hpi to 36 hpi with an MOI of 1 (n=4), related to Fig.2d.

Data are representative of three independent experiments. Data are shown as the mean ± SEM. Analysis is performed using the survival curve comparison (**a-c**, log-rank [Mantel–Cox] test), two-sided unpaired Student's *t* test (**d**), or one-way ANOVA (**e-g**, Dunnett's multiple comparisons test). The exact *p* values and sample numbers are shown in the figure or figure legends. * $p < 0.05$, ** $p < 0.01$, *** $p < 0.001$. Source data are provided as a Source Data file.

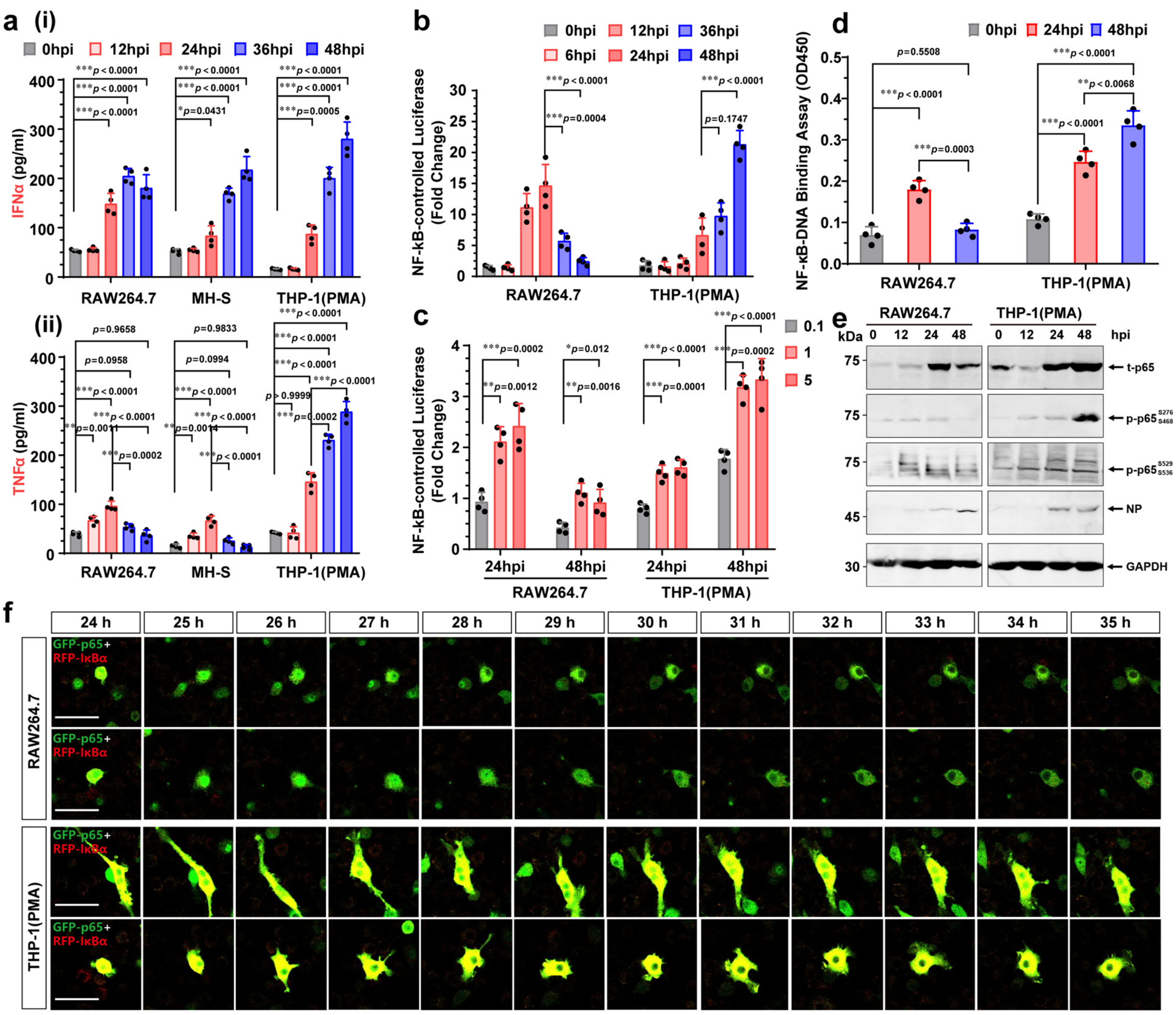

Suppl. Fig.5: Macrophage Reprogramming Processes Mediated by the NF-κB Pathway post HTNV Infection, Related to Fig.2

(**a**) The IFNα or TNFα concentration of cell supernatants measured by ELISA (n=4).

(**b** and **c**) Analysis of p65 activity with time (**b**) or dose effects (**c**) by dual-luciferase reporter assays (n=4).

(**d**) The DNA binding capacity of p65 from 0 to 48 hpi with an MOI of 1 (n=4).

(**e**) Representative immunoblot analysis of p65 and Stat1 signaling at different time points with an MOI of 1 (n=4) of three independent experiments.

(**f**) Representative live cell imaging depicting the translocation of GFP-p65 in the cytoplasm and nucleus at the late HTNV infection phase of three independent experiments. Scale bars, 50 μm.

Data are representative of three independent experiments. Data are shown as the mean ± SEM. Analysis is performed using one-way ANOVA (**a**-**d**, Dunnett's multiple comparisons test). The exact *p* values and sample numbers are shown in the figure or figure legends. * $p < 0.05$, ** $p < 0.01$, *** $p < 0.001$. Molecular weight markers are shown to the left of the blots in kDa, and antibodies used are indicated to the right. Source data are provided as a Source Data file.

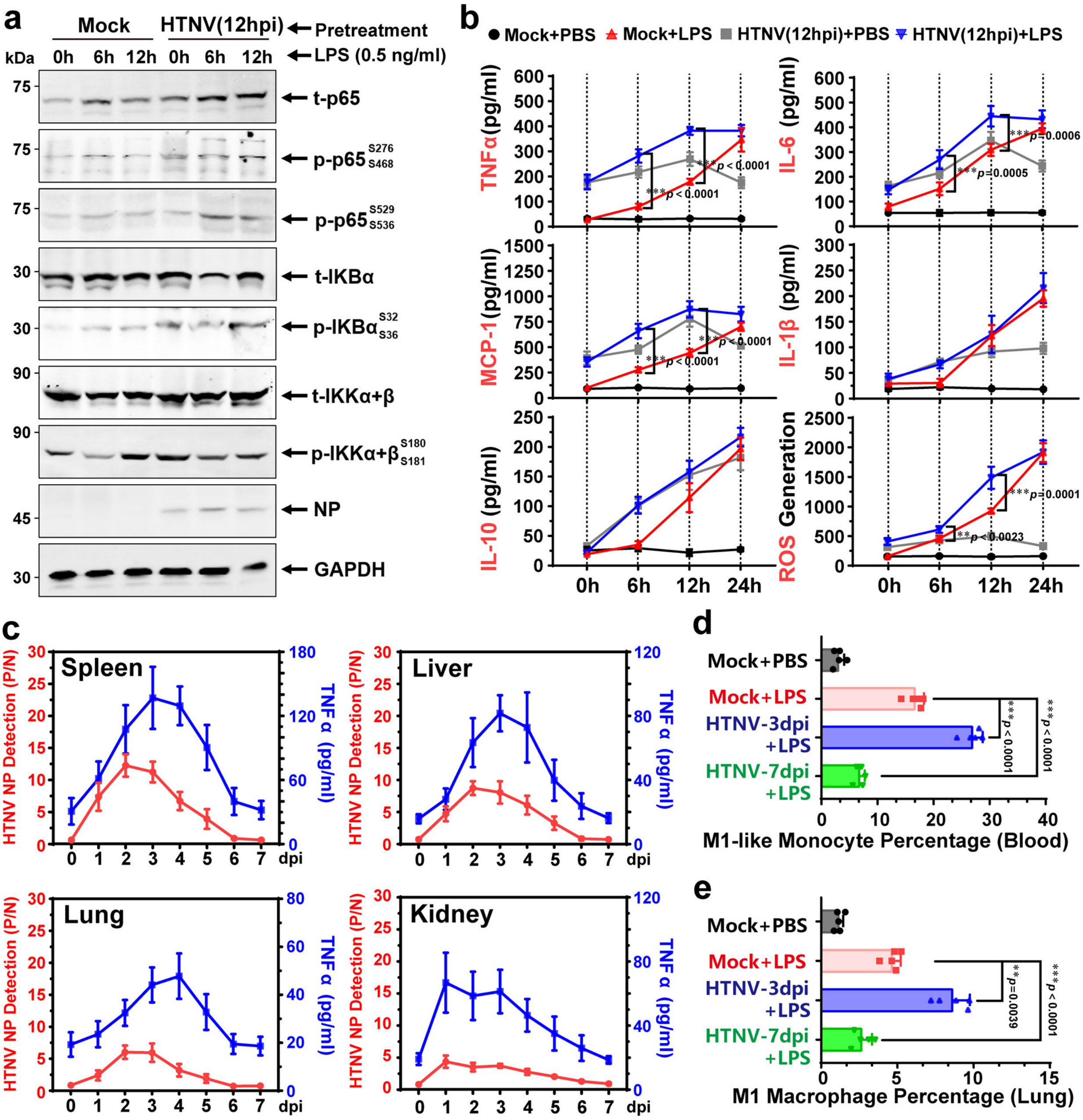

a
Mock
HTNV(12hpi)
Pretreatment
kDa
0h 6h 12h 0h 6h 12h
LPS (0.5 ng/ml)
75
t-p65
75
p-p65 S276 S468
75
p-p65 S529 S536
30
t-IKBα
30
p-IKBα S32 S36
90
t-IKKα+β
90
p-IKKα+β S180 S181
45
NP
30
GAPDH
b
Mock+PBS
Mock+LPS
HTNV(12hpi)+PBS
HTNV(12hpi)+LPS
TNFα(pg/ml)
IL-6 (pg/ml)
MCP-1(pg/ml)
IL-1β (pg/ml)
IL-10 (pg/ml)
ROS Generation
***p < 0.0001
***p=0.0006
***p=0.0005
**p=0.0023
***p=0.0001
0h 6h 12h 24h
c
Spleen
Liver
Lung
Kidney
HTNV NP Detection (P/N)
TNF α (pg/ml)
dpi
d
Mock+PBS
Mock+LPS
HTNV-3dpi +LPS
HTNV-7dpi +LPS
***p < 0.0001
M1-like Monocyte Percentage (Blood)
e
Mock+PBS
Mock+LPS
HTNV-3dpi +LPS
HTNV-7dpi +LPS
**p=0.0039
***p < 0.0001
M1 Macrophage Percentage (Lung)

Suppl. Fig.6: Macrophage Reprograming by HTNV Protect Mice from Secondary LPS challenge, Related to Fig.3

(**a** and **b**) Immunoblot analysis evaluating the NF-κB pathway (**a**) and ELISA results measuring cytokine production from supernatants or cellular ROS production (**b**, n=4).

(**c**) HTNV-NP production (left) and TNFα concentration (right) assessed by ELISA in various tissues of HTNV-infected mice (i.m., 8×10^5 $TCID_{50}$/g) from 0 to 7 dpi (n=4).

(**d** and **e**) Statistical analysis for flow cytometry assays, (**d**) for Fig.3e and (**e**) for Fig.3f (n=5).

Data are shown as the mean ± SEM. Data are representative of two independent experiments. Analysis is performed using two-sided unpaired Student's *t* test (**b**), or one-way ANOVA (**d** and **e**, Dunnett's multiple comparisons test). The exact *p* values and sample numbers are shown in the figure or figure legends. * $p < 0.05$, ** $p < 0.01$, *** $p < 0.001$. Molecular weight markers are shown to the left of the blots in kDa, and antibodies used are indicated to the right. Source data are provided as a Source Data file.

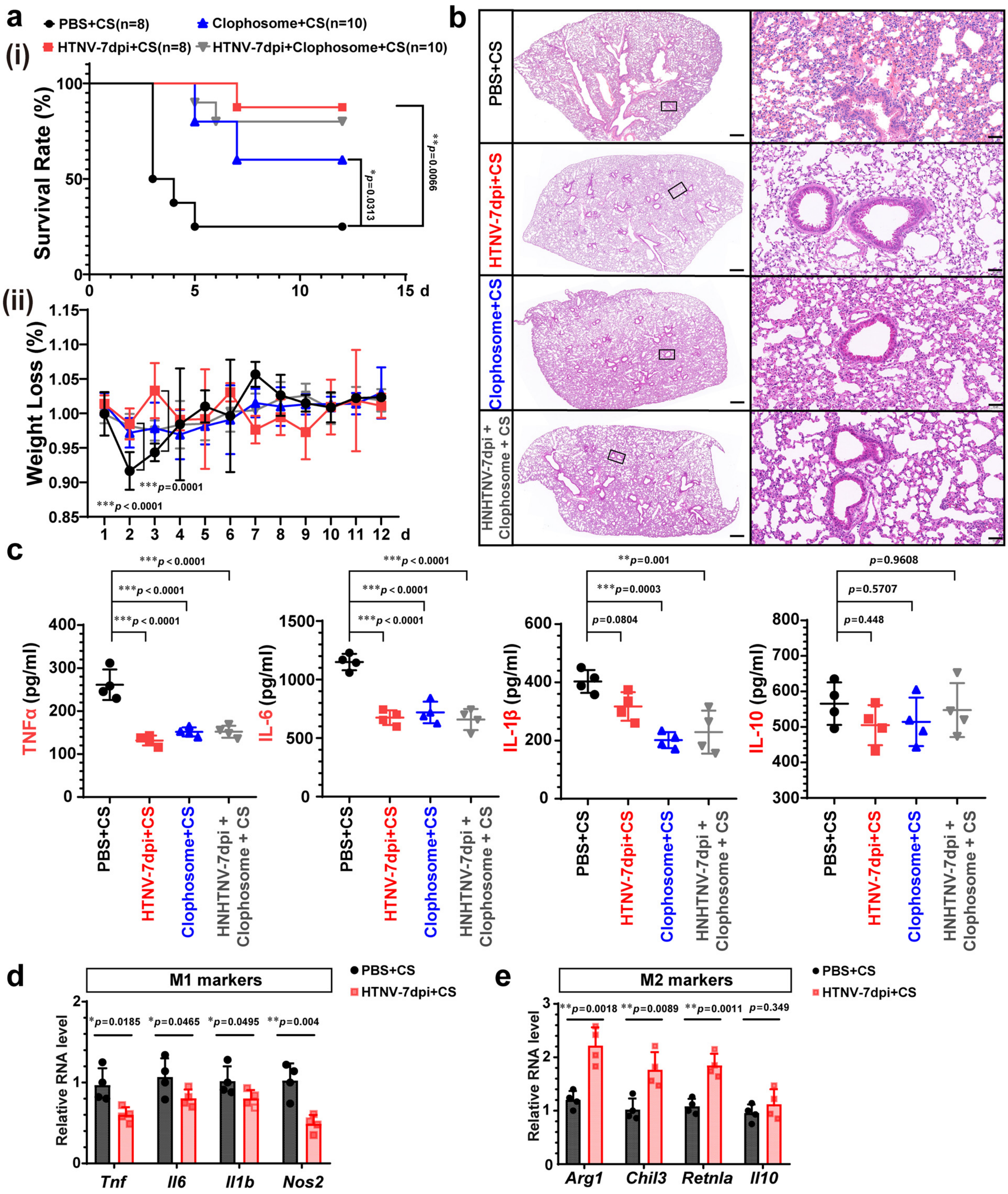
a
PBS+CS(n=8)
Clophosome+CS(n=10)
HTNV-7dpi+CS(n=8)
HTNV-7dpi+Clophosome+CS(n=10)
(i)
Survival Rate (%)
*p=0.0313
**p=0.0066
(ii)
Weight Loss (%)
***p=0.0001
***p < 0.0001
b
PBS+CS
HTNV-7dpi+CS
Clophosome+CS
HNHTNV-7dpi + Clophosome + CS
c
TNFα (pg/ml)
IL-6 (pg/ml)
IL-1β (pg/ml)
IL-10 (pg/ml)
***p < 0.0001
**p=0.001
***p=0.0003
p=0.0804
p=0.9608
p=0.5707
p=0.448
d
M1 markers
PBS+CS
HTNV-7dpi+CS
Relative RNA level
*p=0.0185
*p=0.0465
*p=0.0495
**p=0.004
Tnf
Il6
Il1b
Nos2
e
M2 markers
**p=0.0018
**p=0.0089
**p=0.0011
p=0.349
Arg1
Chil3
Retnla
Il10

Suppl. Fig.7: Late-phase Inactivation of M1 Defends Rodents against Lethal Polymicrobial Sepsis, Related to Fig.3

(**a**) Survival and weight loss data of mice pretreated with HTNV (i.m., 8×10^5 $TCID_{50}$/g) or/and clophosome (i.p., 10 µl/g), and then challenged with CS (i.p., 0.6 mg/g).
(**b**) H&E staining of lungs at 3 d post-CS stimulation from (**a**). Scale bars, 200 µm (left) or 20 µm (right).
(**c**) Serum cytokine concentration detected by ELISA at 3 d post-CS stimulation from (**a**, n=4).
(**d** and **e**) M1 (**d**) or M2 (**e**) macrophage polarization markers assayed by qRT-PCR of mouse AMs at 3 days post CS stimulation from (**a**, n=4). Target gene mRNA is normalized to GAPDH mRNA.
Data are shown as the mean ± SEM. Data are representative of two independent experiments. Analysis is performed using the survival curve comparison (**a-(i)**, log-rank [Mantel–Cox] test), one-way ANOVA (**c**, Dunnett's multiple comparisons test), or two-sided unpaired Student's *t* test (**d** and **e**). The exact *p* values and sample numbers are shown in the figure or figure legends. * $p < 0.05$, ** $p < 0.01$, *** $p < 0.001$. Source data are provided as a Source Data file.

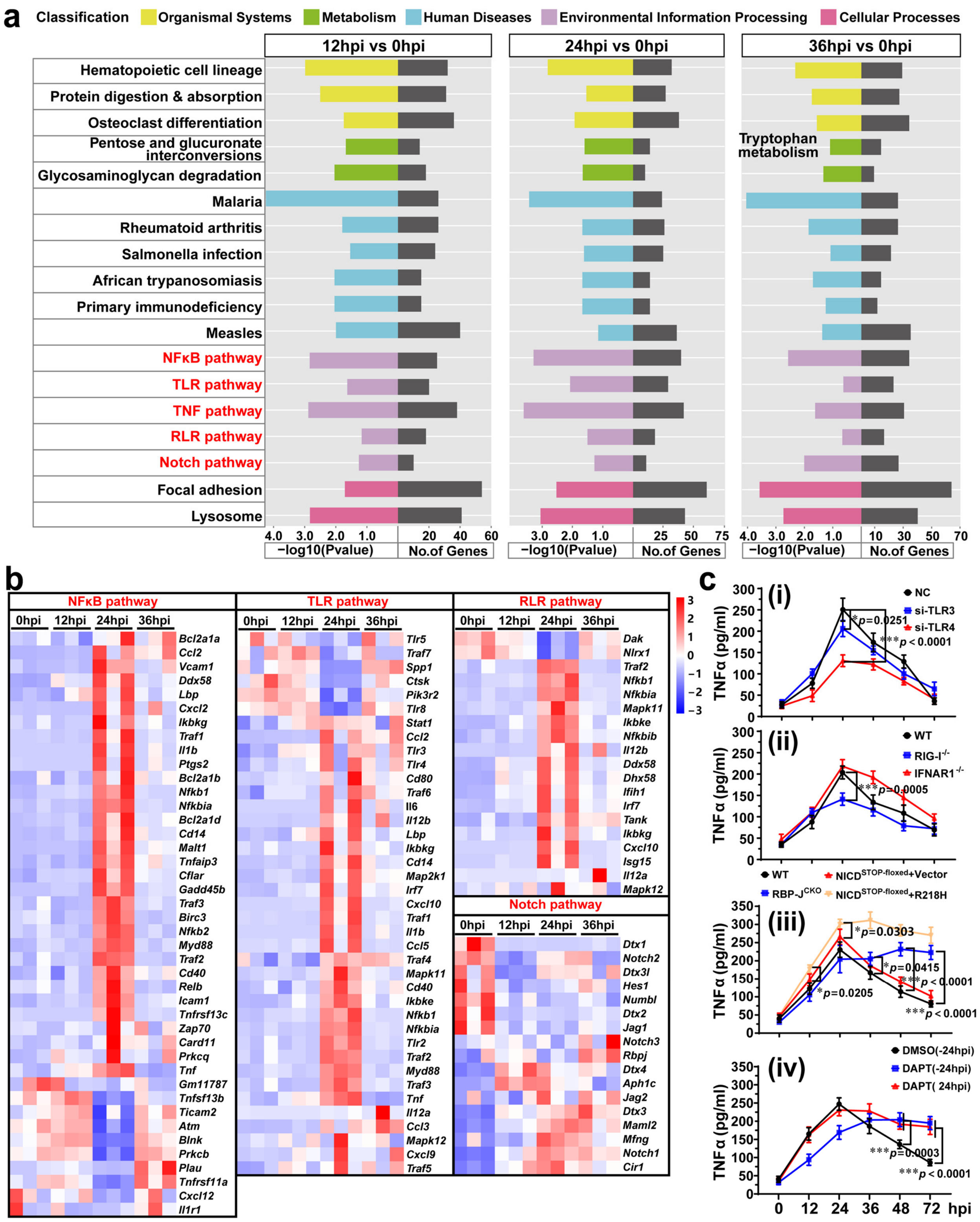
a
Classification
Organismal Systems
Metabolism
Human Diseases
Environmental Information Processing
Cellular Processes
12hpi vs 0hpi
24hpi vs 0hpi
36hpi vs 0hpi
Hematopoietic cell lineage
Protein digestion & absorption
Osteoclast differentiation
Pentose and glucuronate interconversions
Glycosaminoglycan degradation
Malaria
Rheumatoid arthritis
Salmonella infection
African trypanosomiasis
Primary immunodeficiency
Measles
NFκB pathway
TLR pathway
TNF pathway
RLR pathway
Notch pathway
Focal adhesion
Lysosome
Tryptophan metabolism
−log10(Pvalue)
No.of Genes
b
NFκB pathway
TLR pathway
RLR pathway
Notch pathway
0hpi
12hpi
24hpi
36hpi
Bcl2a1a Ccl2 Vcam1 Ddx58 Lbp Cxcl2 Ikbkg Traf1 Il1b Ptgs2 Bcl2a1b Nfkb1 Nfkbia Bcl2a1d Cd14 Malt1 Tnfaip3 Cflar Gadd45b Traf3 Birc3 Nfkb2 Myd88 Traf2 Cd40 Relb Icam1 Tnfrsf13c Zap70 Card11 Prkcq Tnf Gm11787 Tnfsf13b Ticam2 Atm Blnk Prkcb Plau Tnfrsf11a Cxcl12 Il1r1
Tlr5 Traf7 Spp1 Ctsk Pik3r2 Tlr8 Stat1 Ccl2 Tlr3 Tlr4 Cd80 Traf6 Il6 Il12b Lbp Ikbkg Cd14 Map2k1 Irf7 Cxcl10 Traf1 Il1b Ccl5 Traf4 Mapk11 Cd40 Ikbke Nfkb1 Nfkbia Tlr2 Traf2 Myd88 Traf3 Tnf Il12a Ccl3 Mapk12 Cxcl9 Traf5
Dak Nlrx1 Traf2 Nfkb1 Nfkbia Mapk11 Ikbke Nfkbib Il12b Ddx58 Dhx58 Ifih1 Irf7 Tank Ikbkg Cxcl10 Isg15 Il12a Mapk12
Dtx1 Notch2 Dtx3l Hes1 Numbl Dtx2 Jag1 Notch3 Rbpj Dtx4 Aph1c Jag2 Dtx3 Maml2 Mfng Notch1 Cir1
3 2 1 0 −1 −2 −3
c
(i)
NC
si-TLR3
si-TLR4
*p=0.0251
***p < 0.0001
TNFα (pg/ml)
(ii)
WT
RIG-I-/-
IFNAR1-/-
***p=0.0005
TNF α (pg/ml)
(iii)
WT
NICDSTOP-floxed+Vector
RBP-JCKO
NICDSTOP-floxed+R218H
*p=0.0303
*p=0.0415
***p < 0.0001
*p=0.0205
***p < 0.0001
(iv)
DMSO(-24hpi)
DAPT(-24hpi)
DAPT(24hpi)
***p=0.0003
***p < 0.0001
0 12 24 36 48 72 hpi

Suppl. Fig.8: Pathway Analysis for Macrophage Phenotype post HTNV Infection, Related to Fig.4

(**a**) GO term and KEGG pathway enrichment analysis of infected macrophages (12, 24 and 36 hpi) versus controls (0 hpi).

(**b**) Heatmap showing gene expression alterations associated with NF-κB, TLR, RLR and Notch signaling.

(**c**) Supernatant TNFα concentration assessed by ELISA (n=4).

(-i) The mBMDM from the WT mice are transfected with negative control (NC) siRNAs or siRNAs for silencing TLR3 (si-TLR3) or TLR4 (si-TLR4) for 24 hr and then infected with HTNV.

(-ii) The mBMDM generated from the WT, $RIG^{-/-}$ or $IFNAR1^{-/-}$ mice are challenged with HTNV.

(-iii) The mBMDM generated from the WT, $RBP\text{-}J^{CKO}$ (Lyz2-Cre × $RBP\text{-}J^{floxed}$, conditionally inhibiting Notch signaling in monocytes and macrophages by knocking out RBP-J) or $NICD^{STOP\text{-}floxed}$ (Lyz2-Cre × $NICD^{STOP\text{-}floxed}$, conditionally activating the Notch pathway in monocytes and macrophages by overexpressing NICD) mice are infected with HTNV, of which the $NICD^{STOP\text{-}floxed}$ mBMDM are electrotransfected with the control or vector encoding R218H.

(-iv) The mBMDM from the WT mice are pretreated with DMSO or DAPT (50 μmol/L) for 24 hr (-24h group), or treated at 24 hpi (24 h group) and then infected with HTNV.

Data are shown as the mean ± SEM and are representative of three independent experiments. Analysis is performed using one-way ANOVA (**c**, Dunnett's multiple comparisons test). The exact *p* values and sample numbers are shown in the figure or figure legends. * $p < 0.05$, ** $p < 0.01$, *** $p < 0.001$. Source data are provided as a Source Data file.

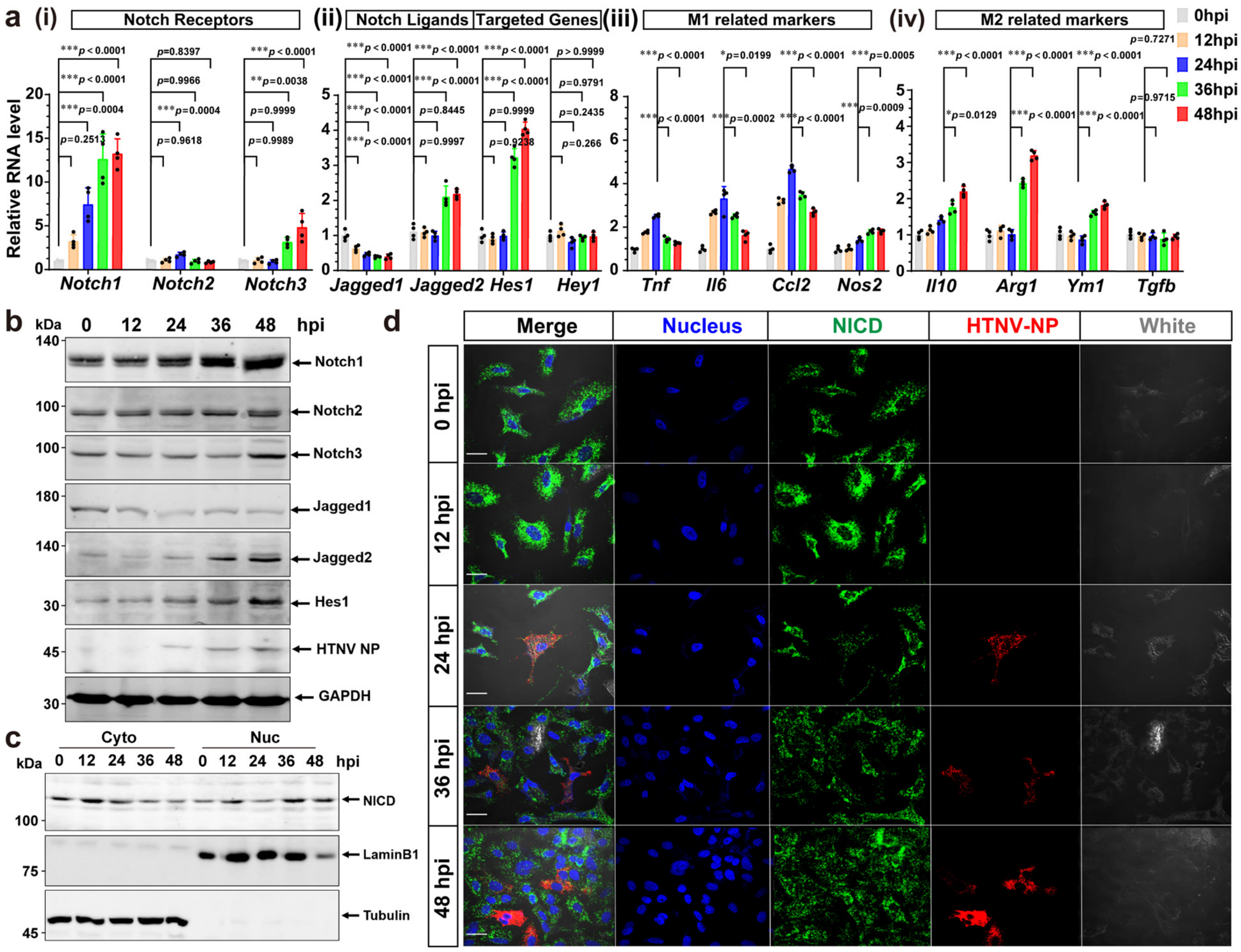

Suppl. Fig.9: HTNV Dynamically Regulates the Notch Pathway in Murine Macrophage *in vitro*, Related to Fig.4

(**a**) The mRNA measurement of WT mBMDM from 0 hpi to 48 hpi by qRT-PCR for murine Notch receptors (i), ligands and target genes (ii), as well as the M1 (iii)- or M2 (iv)-related genes, normalized to *Gapdh* (MOI=1, n=4).

(**b**) Representative immunoblot analysis of the Notch pathway in mBMDM at an MOI of 1 of three independent experiments.

(**c**) Representative immunoblot analysis of the NICD amount in the cytoplasm or nucleus in mBMDM at an MOI of 1 of three independent experiments.

(**d**) Representative immunofluorescence assays performed to observe the expression and subcellular localization of NICD in WT mBMDM (MOI=1) of three independent experiments. Scale bars, 10 μm. Data are shown as the mean ± SEM and are representative of three independent experiments. Target gene mRNA is normalized to GAPDH mRNA. Analysis is performed using one-way ANOVA (**a**, Dunnett's multiple comparisons test). The exact p values and sample numbers are shown in the figure or figure legends. * $p < 0.05$, ** $p < 0.01$, *** $p < 0.001$. Molecular weight markers are shown to the left of the blots in kDa, and antibodies used are indicated to the right. Source data are provided as a Source Data file.

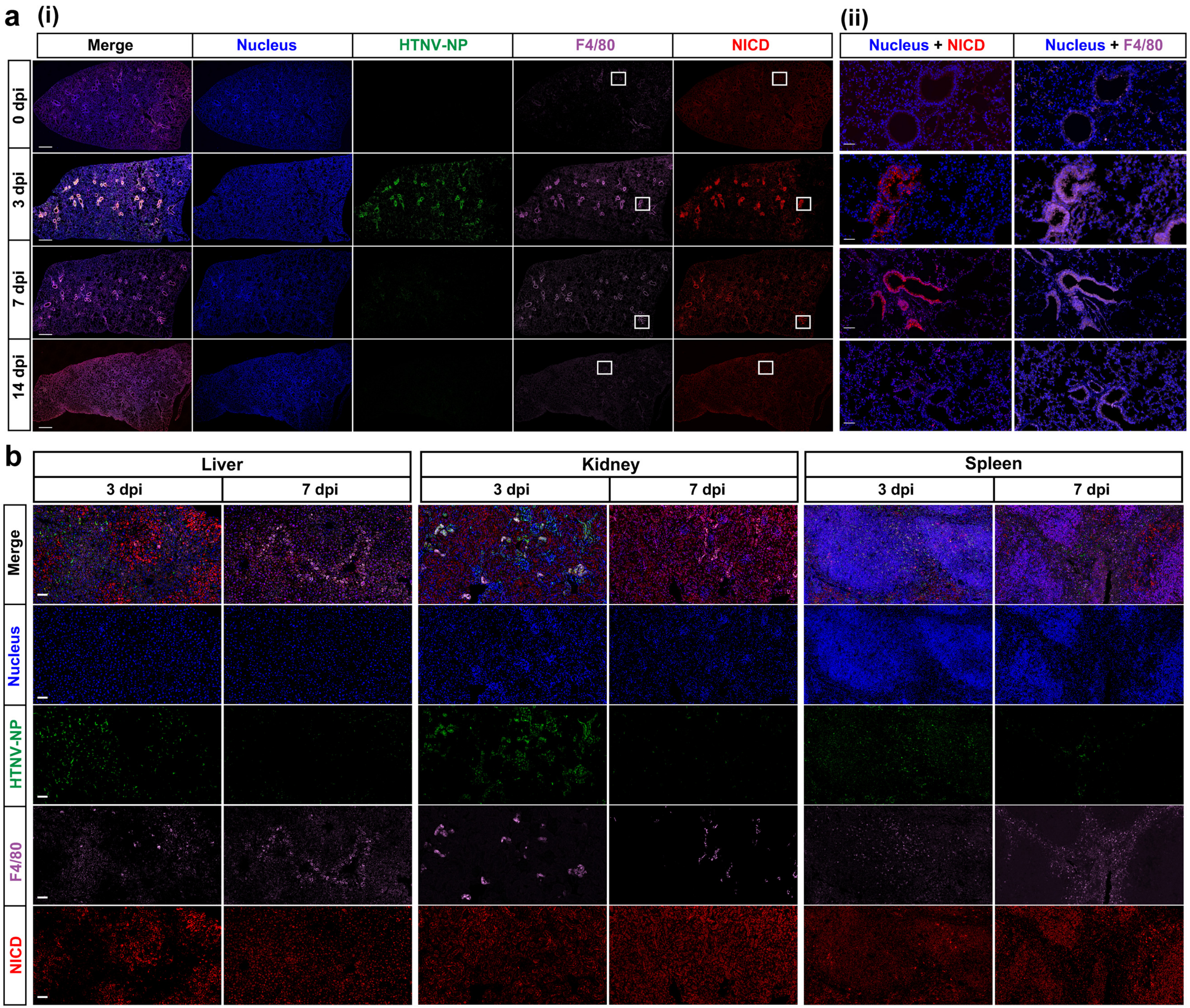

a
(i)
Merge
Nucleus
HTNV-NP
F4/80
NICD
(ii)
Nucleus + NICD
Nucleus + F4/80
0 dpi
3 dpi
7 dpi
14 dpi
b
Liver
Kidney
Spleen
3 dpi
7 dpi
Merge
Nucleus
HTNV-NP
F4/80
NICD

Suppl. Fig.10: HTNV Dynamically Regulates the Notch Pathway in Murine Macrophage *in vivo*, Related to Fig.4

(**a**) Representative immunofluorescence assays for the subcellular localization of NICD in lung tissue (8×10^5 $TCID_{50}$/g) of three independent experiments (n=4 for each test). Scale bars, 500 µm in (i) and 20 µm in (ii).

(**b**) Representative immunofluorescence analysis for the subcellular localization of NICD in F4/80^+ macrophages from multiple mouse tissues at 3 dpi and 7 dpi of three independent experiments (n=4 for each test). Scale bars, 20 µm.

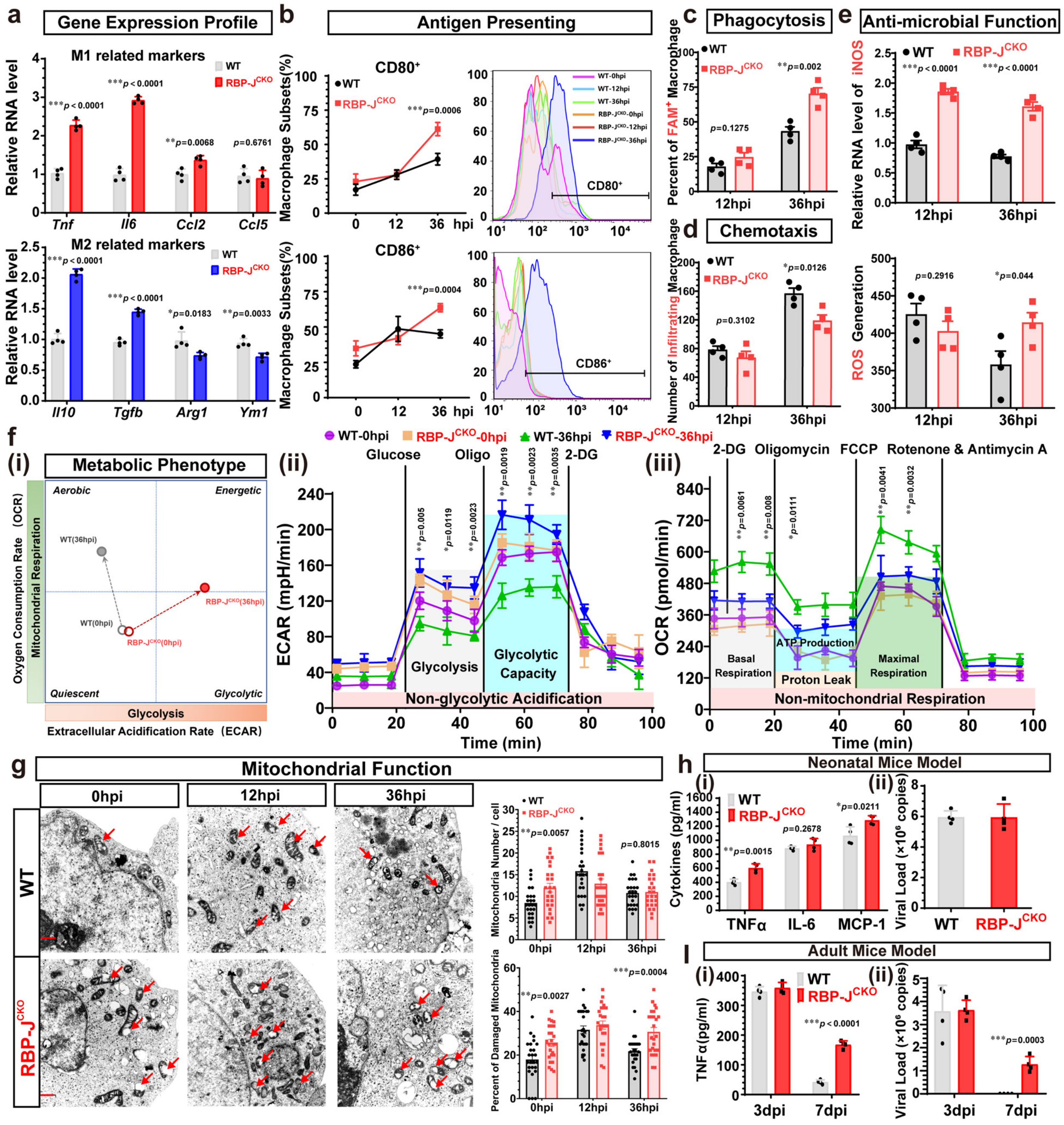

a
Gene Expression Profile
M1 related markers
M2 related markers
Relative RNA level
Tnf
Il6
Ccl2
Ccl5
Il10
Tgfb
Arg1
Ym1
WT
RBP-J^CKO
b
Antigen Presenting
CD80⁺
CD86⁺
Macrophage Subsets(%)
hpi
c
Phagocytosis
Percent of FAM⁺ Macrophage
d
Chemotaxis
Number of Infiltrating Macrophage
e
Anti-microbial Function
Relative RNA level of iNOS
ROS Generation
12hpi
36hpi
f
(i)
Metabolic Phenotype
Aerobic
Energetic
Quiescent
Glycolytic
Oxygen Consumption Rate (OCR)
Mitochondrial Respiration
Glycolysis
Extracellular Acidification Rate (ECAR)
WT-0hpi
RBP-J^CKO-0hpi
WT-36hpi
RBP-J^CKO-36hpi
(ii)
Glucose
Oligo
2-DG
Glycolysis
Glycolytic Capacity
Non-glycolytic Acidification
ECAR (mpH/min)
Time (min)
(iii)
2-DG
Oligomycin
FCCP
Rotenone & Antimycin A
Basal Respiration
ATP Production
Proton Leak
Maximal Respiration
Non-mitochondrial Respiration
OCR (pmol/min)
Time (min)
g
Mitochondrial Function
0hpi
12hpi
36hpi
WT
RBP-J^CKO
Mitochondria Number / cell
Percent of Damaged Mitochondria
h
Neonatal Mice Model
(i)
(ii)
Cytokines (pg/ml)
TNFα
IL-6
MCP-1
Viral Load (×10⁶ copies)
l
Adult Mice Model
TNFα(pg/ml)
3dpi
7dpi

Suppl. Fig.11: The Notch Pathway Determines the Murine Macrophage Phenotype Shift, Related to Fig.4

(**a**) qRT-PCR for the expression of M1 or M2 related genes 36 hpi (MOI=1, n=4).
(**b**) Flow cytometry analysis for of CD80/86 expression at 36 hpi (MOI=1, n=4).
(**c**) The phagocytic capacity evaluated by calculating the percentage of FAM^+ cells. Scale bars, 100 μm.
(**d**) The chemotaxis ability evaluated by calculating the infiltrated cells based on the transwell system. Scale bars, 100 μm.
(**e**) iNOS mRNA expression by qRT-PCR and cellular ROS production by H2DCFDA staining (n=4).
(**f**) Metabolic phenotype shift (-i), ECAR (-ii) and OCR (-iii) assays by Seahorse (MOI=1, n=3). The two-sided unpaired Student's *t* test is performed, WT-36hpi vs. $RBP\text{-}J^{CKO}$-36hpi.
(**g**) The mitochondrial morphology evaluated with transmission electron microscopy (TEM). Left, TEM imaging; Right, calculation for the mitochondrial number and related damage percentage of each cell (25 cells are computed for each group).
(**h**) Serum cytokine concentration (ELISA) (-i) and viral load (qRT-PCR) (-ii) at 7 dpi from Fig.4e (n=4).
(**i**) Serum TNFα concentration (ELISA) (-i) and viral load (qRT-PCR) (-ii) at 3 dpi or 7 dpi from Fig.4f (i.p., 8×10^5 $TCID_{50}$/g, n=4).

Data are shown as the mean ± SEM and are representative of three independent experiments. Target gene mRNA is normalized to GAPDH mRNA. Analysis is performed using two-sided unpaired Student' s *t* test. The exact *p* values and sample numbers are shown in the figure or figure legends. * $p < 0.05$, ** $p < 0.01$, *** $p < 0.001$. Source data are provided as a Source Data file.

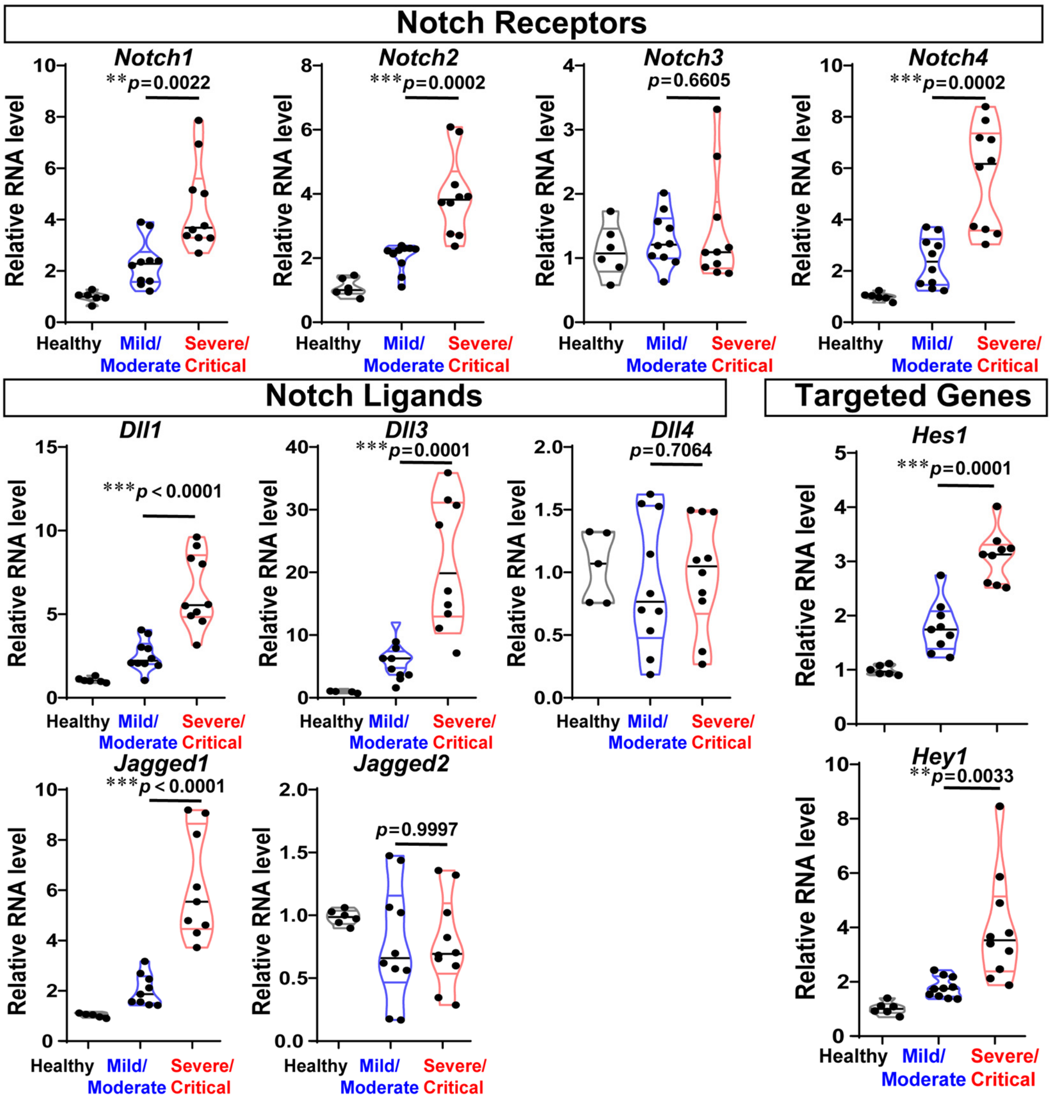

Suppl. Fig.12: The Expression of Notch Pathway-related Genes in Monocytes Correlates with the HFRS Severity, Related to Fig.5

The expression levels of Notch receptors, ligands and target genes in monocytes from healthy individuals and HFRS patients with various disease severity are evaluated by qRT-PCR. Data are shown as the median, quartile, and standard deviation. Analysis is performed using two-sided unpaired Student's *t* test. Target gene mRNA is normalized to GAPDH mRNA. The exact *p* values and sample numbers are shown in the figure or figure legends. * $p < 0.05$, ** $p < 0.01$, *** $p < 0.001$. Source data are provided as a Source Data file.

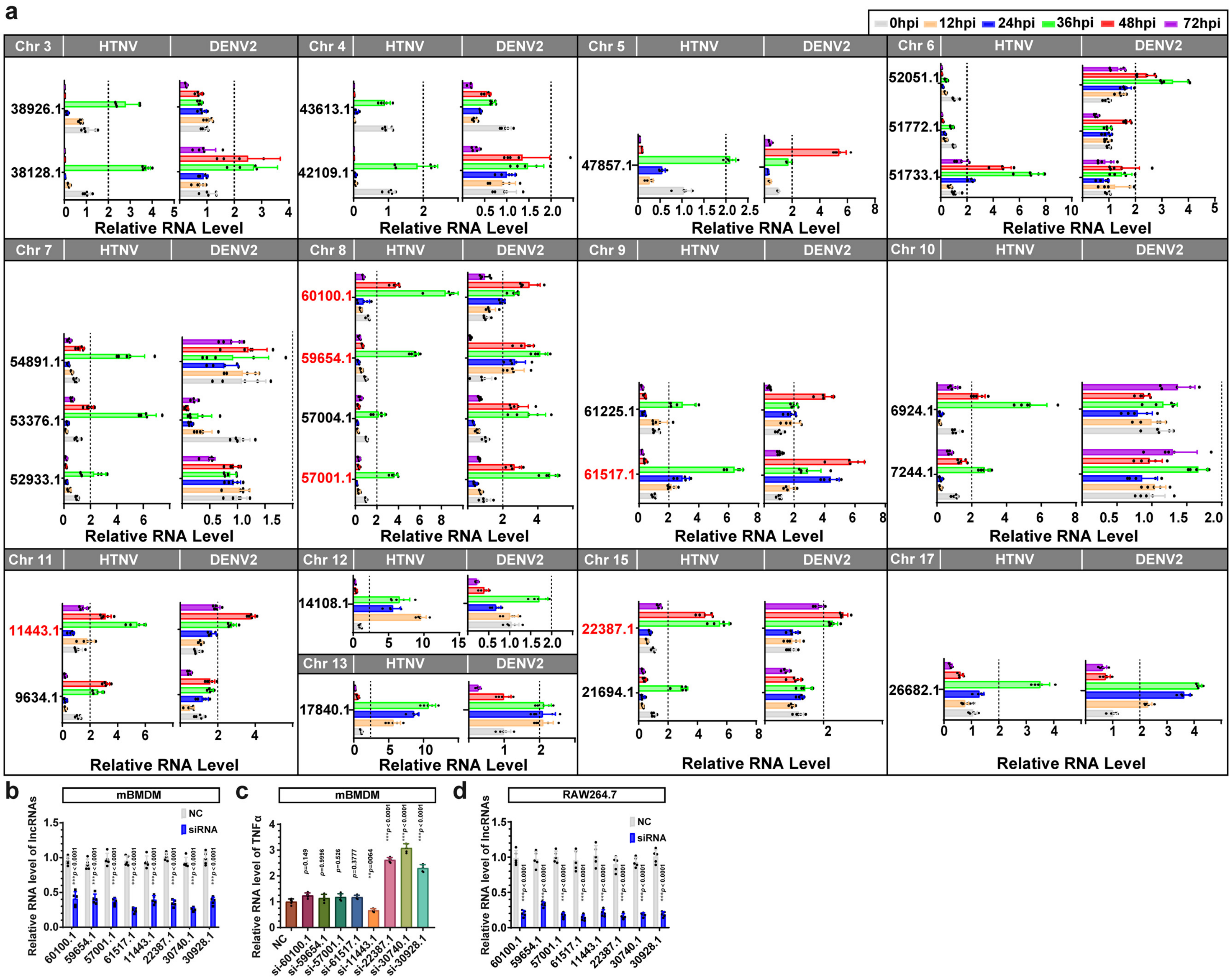
a
0hpi 12hpi 24hpi 36hpi 48hpi 72hpi
Chr 3 HTNV DENV2
38926.1
38128.1
Chr 4 HTNV DENV2
43613.1
42109.1
Chr 5 HTNV DENV2
47857.1
Chr 6 HTNV DENV2
52051.1
51772.1
51733.1
Chr 7 HTNV DENV2
54891.1
53376.1
52933.1
Chr 8 HTNV DENV2
60100.1
59654.1
57004.1
57001.1
Chr 9 HTNV DENV2
61225.1
61517.1
Chr 10 HTNV DENV2
6924.1
7244.1
Chr 11 HTNV DENV2
11443.1
9634.1
Chr 12 HTNV DENV2
14108.1
Chr 13 HTNV DENV2
17840.1
Chr 15 HTNV DENV2
22387.1
21694.1
Chr 17 HTNV DENV2
26682.1
Relative RNA Level
b
mBMDM
NC
siRNA
Relative RNA level of lncRNAs
60100.1 59654.1 57001.1 61517.1 11443.1 22387.1 30740.1 30928.1
c
mBMDM
Relative RNA level of TNFα
NC si-60100.1 si-59654.1 si-57001.1 si-61517.1 si-11443.1 si-22387.1 si-30740.1 si-30928.1
p=0.149 p=0.9996 p=0.526 p=0.3777 **p=.0064 ****p<0.0001 ****p<0.0001 ****p<0.0001
d
RAW264.7
NC
siRNA
Relative RNA level of lncRNAs
60100.1 59654.1 57001.1 61517.1 11443.1 22387.1 30740.1 30928.1

Suppl. Fig.13: A Cluster of Murine-specific LncRNAs Identified in HTNV-infected Macrophages, Related to Fig.6

(**a**) qRT-PCR analysis of the indicated lncRNAs shown in Fig.6a in HTNV- or DENV2-infected mBMDM from 0 to 72 hpi (MOI=1, grouped by their location on chromosomes, n=5).
(**b**) qRT-PCR detection for lncRNA silencing efficiency in mBMDM (n=5).
(**c**) qRT-PCR analysis of TNFα mRNA expression in mBMDM at 36 hpi (n=4).
(**d**) qRT-PCR detection for lncRNA silencing efficiency in RAW264.7 (n=4).
Data are shown as the mean ± SEM, and are representative of three independent experiments. Target gene mRNA is normalized to GAPDH mRNA. Analysis is performed using two-sided unpaired Student's *t* test. The exact *p* values and sample numbers are shown in the figure or figure legends. * $p < 0.05$, ** $p < 0.01$, *** $p < 0.001$. Source data are provided as a Source Data file.

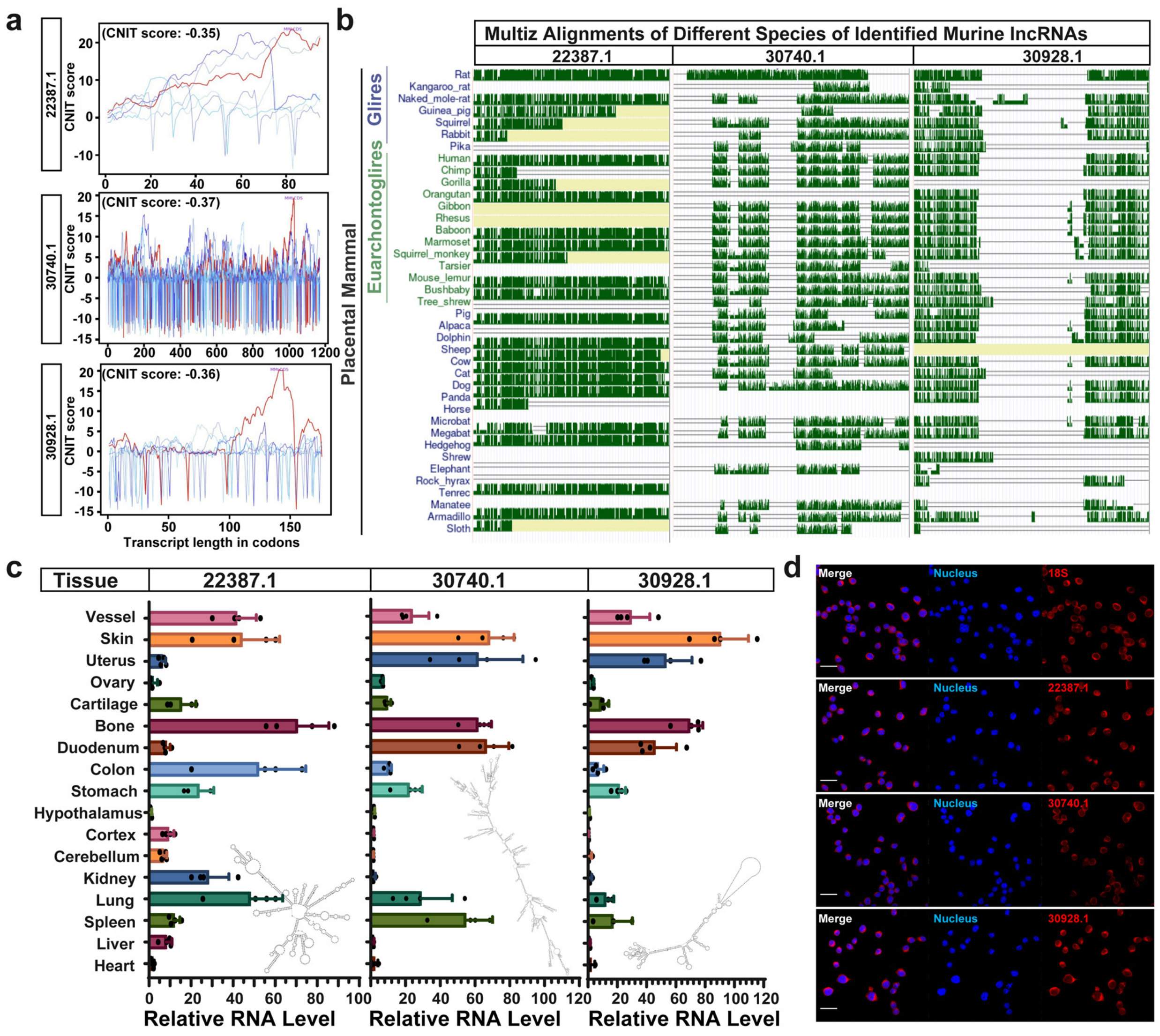

Suppl. Fig.14: Characteristics of Identified Murine-specific LncRNAs, Related to Fig.6

(**a**) Potential coding ability of the indicated lncRNAs by CNIT.

The red line represents the correct transcriptional reading frame, and the other five lines represent the other reading frames. The green line indicates the distribution of the coverage (y-axis) of the MLCDS region for each transcript across the normalized length. MLCDS, the most-like CDS region. Codon length, the total length of the identified sequence converted to codon length (the identified sequence length/3).

(**b**) Sequence alignment for the indicated lncRNAs based on the UCSC Genome Browser database.

(**c**) qRT-PCR analysis for indicated lncRNA in multiple tissues (normalized to the heart, n=4) and RNAfold prediction for their secondary structure. Target gene mRNA is normalized to GAPDH mRNA.

(**d**) FISH analysis of the indicated lncRNAs in mBMDM (18S detection as the control). Scale bars, 20 μm. Data are shown as the mean ± SEM. Data are representative of three independent experiments. Source data are provided as a Source Data file.

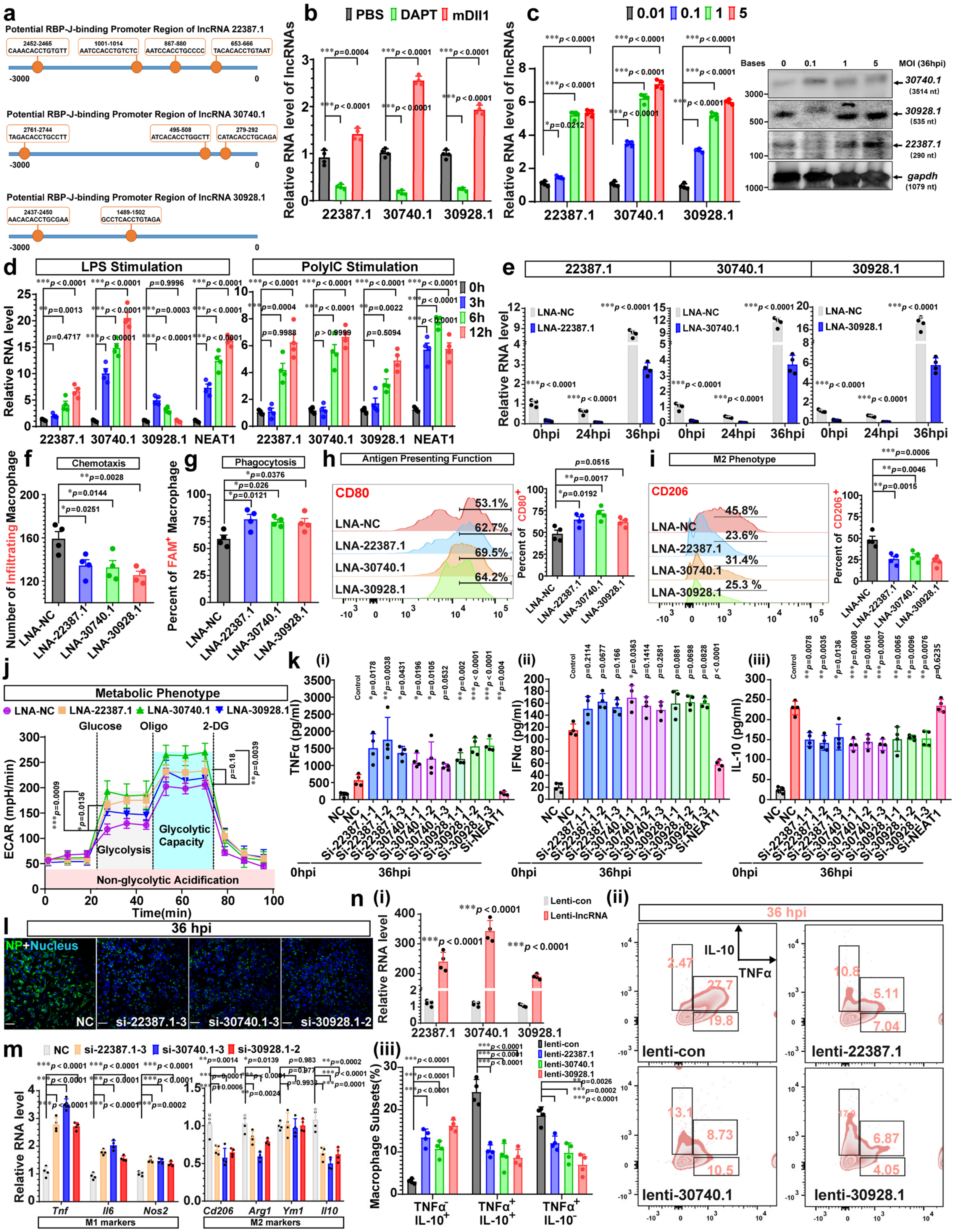
a
Potential RBP-J-binding Promoter Region of lncRNA 22387.1
Potential RBP-J-binding Promoter Region of lncRNA 30740.1
Potential RBP-J-binding Promoter Region of lncRNA 30928.1
b
PBS DAPT mDll1
Relative RNA level of lncRNAs
c
0.01 0.1 1 5
Bases 0 0.1 1 5 MOI (36hpi)
30740.1 (3514 nt)
30928.1 (535 nt)
22387.1 (290 nt)
gapdh (1079 nt)
d
LPS Stimulation
PolyIC Stimulation
Relative RNA level
0h 3h 6h 12h
22387.1 30740.1 30928.1 NEAT1
e
22387.1 30740.1 30928.1
LNA-NC LNA-22387.1 LNA-30740.1 LNA-30928.1
0hpi 24hpi 36hpi
f
Chemotaxis
Number of Infiltrating Macrophage
g
Phagocytosis
Percent of FAM+ Macrophage
h
Antigen Presenting Function
CD80
i
M2 Phenotype
CD206
j
Metabolic Phenotype
Glucose Oligo 2-DG
ECAR (mpH/min)
Time(min)
Glycolysis
Glycolytic Capacity
Non-glycolytic Acidification
k
TNFα (pg/ml)
IFNα (pg/ml)
IL-10 (pg/ml)
0hpi 36hpi
l
36 hpi
NP+Nucleus
NC si-22387.1-3 si-30740.1-3 si-30928.1-2
m
NC si-22387.1-3 si-30740.1-3 si-30928.1-2
Relative RNA level
Tnf Il6 Nos2
M1 markers
Cd206 Arg1 Ym1 Il10
M2 markers
n
Lenti-con Lenti-lncRNA
Macrophage Subsets(%)
TNFα- IL-10+
TNFα+ IL-10+
TNFα+ IL-10-
36 hpi
IL-10
TNFα
lenti-con lenti-22387.1 lenti-30740.1 lenti-30928.1

Suppl. Fig.15: Screened LncRNAs Form Negative Feedback for M1 Polarization, Related to Fig.6

(**a**) The promoter prediction of the indicated lncRNAs.

(**b**) qRT-PCR analysis of the indicated lncRNAs in mBMDM that are pretreated with DAPT (50 μmol/L) or mDll1 (10 ng/ml, n=4).

(**c**) qRT-PCR (left, n=4) and Northern blot (right) analysis of the indicated lncRNAs in mBMDM at 36 hpi.

(**d**) qRT-PCR analysis of the indicated lncRNAs in mBMDM post LPS (5 ng/ml) or polyIC (1 μg/ml stimulation, n=4).

(**e**) qRT-PCR measurement for RNAi efficiency with LNAs (50 nmol/L) in $NICD^{STOP\text{-}floxed}$ mBMDM (n=4).

(**f** and **g**) The chemotaxis (**f**) and phagocytosis (**g**) ability analysis at 36 hpi for mBMDM in Fig.6f (n=4).

(**h** and **i**) Flow cytometry analysis of CD80 (**h**) and CD206 (**i**) at 36 hpi for mBMDM in Fig.6f (n=4).

(**j**) ECAR examination at 36 hpi for mBMDM in Fig.6f (n=3).

(**k**) Supernatant cytokines detected by ELISA at 36 hpi from WT mBMDM with RNAi (n=4).

(**l**) Immunofluorescent analysis of HTNV NP expression in mBMDM in (**k**).

(**m**) qRT-PCR analysis of M1- and M2-related genes in mBMDM in (**k**) (n=4).

(**n**) qRT-PCR analysis to confirm the lncRNA overexpression efficiency in mBMDM with a lentivirus system (-i). Flow cytometry analysis of TNFα and IL-10 expression in $RBP\text{-}J^{CKO}$ mBMDM at 36 hpi (-ii) and the related statistical analysis (-iii) (n=4).

Data are shown as the mean ± SEM, and are representative of three independent experiments. Target gene mRNA is normalized to GAPDH mRNA. Analysis is performed using two-sided unpaired Student' s *t* test (**e** and **n**) or one-way ANOVA (**b-d**, and **f-n**, Dunnett's multiple comparisons test). The exact *p* values and sample numbers are shown in the figure or figure legends. * $p < 0.05$, ** $p < 0.01$, *** $p < 0.001$. RNA markers are shown to the left of the blots in Bases, and RNA probes used are indicated to the right. Source data are provided as a Source Data file.

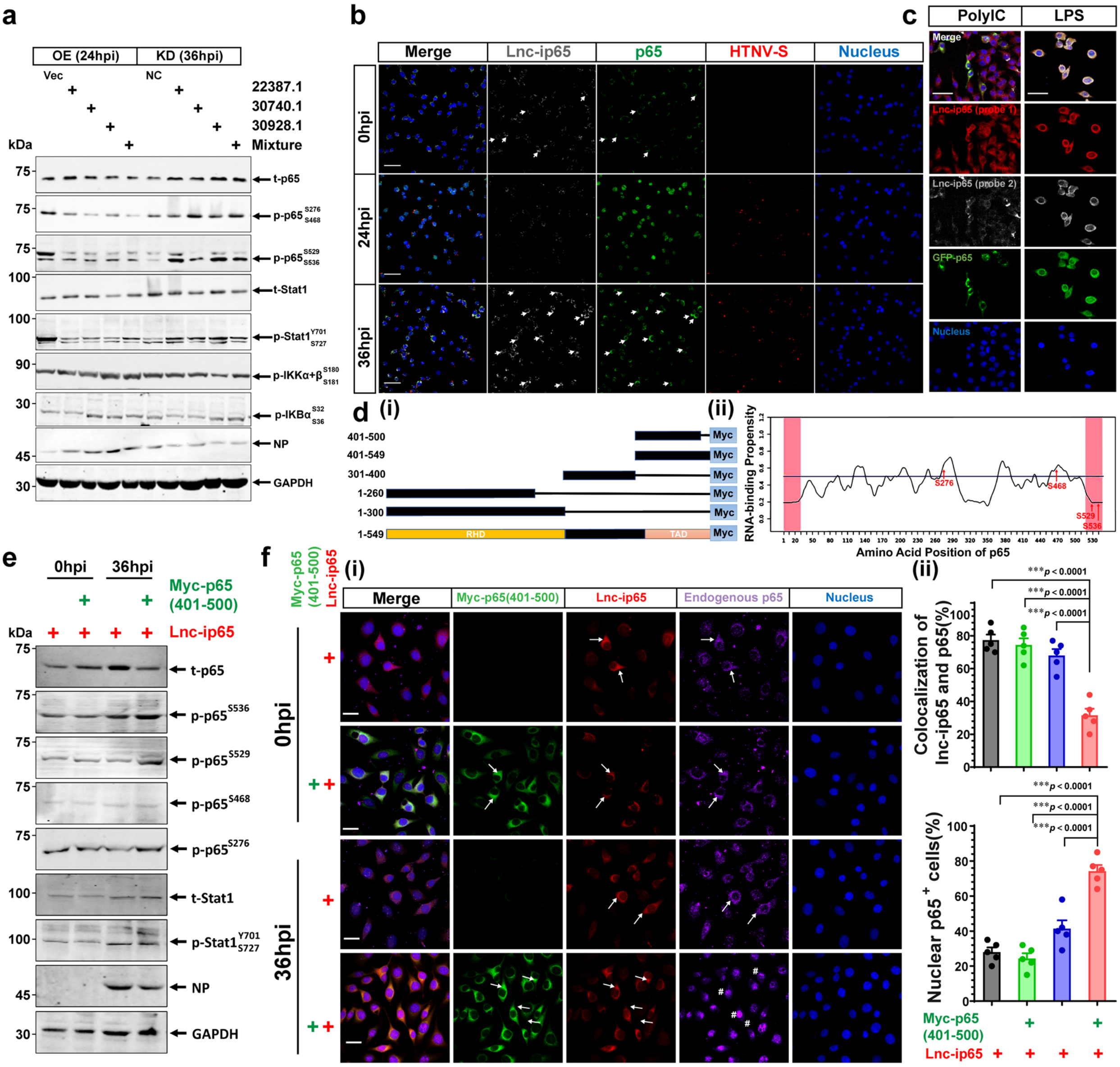
a
OE (24hpi)
KD (36hpi)
Vec
NC
22387.1
30740.1
30928.1
Mixture
kDa
t-p65
p-p65 S276 S468
p-p65 S529 S536
t-Stat1
p-Stat1 Y701 S727
p-IKKα+β S180 S181
p-IKBα S32 S36
NP
GAPDH
b
Merge
Lnc-ip65
p65
HTNV-S
Nucleus
0hpi
24hpi
36hpi
c
PolyIC
LPS
Merge
Lnc-ip65 (probe 1)
Lnc-ip65 (probe 2)
GFP-p65
Nucleus
d (i)
401-500
401-549
301-400
1-260
1-300
1-549
Myc
RHD
TAD
(ii)
RNA-binding Propensity
S276
S468
S529
S536
Amino Acid Position of p65
e
0hpi
36hpi
Myc-p65 (401-500)
Lnc-ip65
t-p65
p-p65 S536
p-p65 S529
p-p65 S468
p-p65 S276
t-Stat1
p-Stat1 Y701 S727
NP
GAPDH
f (i)
Myc-p65 (401-500)
Lnc-ip65
Merge
Myc-p65(401-500)
Endogenous p65
Nucleus
0hpi
36hpi
(ii)
Colocalization of lnc-ip65 and p65(%)
Nuclear p65+ cells(%)
***p < 0.0001
Myc-p65 (401-500)
Lnc-ip65

Suppl. Fig.16: Murine-specific LncRNAs Restrict P65 Activation, Related to Fig.7

(**a**) Representative immunoblot analysis of three independent experiments for mBMDM with the indicated lncRNAs overexpression or knockdown.

(**b**) Representative RNAScope detection images of three independent experiments for lnc-ip65 and HTNV S segment in mBMDM, and immunofluorescent analysis for endogenous p65. Scale bars, 100 μm.

(**c**) Representative immunofluorescent analysis of three independent experiments for lnc-ip65 with different probes (FISH) and GFP-65 in RAW264.7 cells post polyIC (1 μg/ml) or LPS (5 ng/ml) stimulation for 12 hr. Scale bars, 50 μm.

(**d**) Truncation strategy of p65 (-i) and its RNA-binding domain prediction (-ii).

(**e**) Representative immunoblot analysis of three independent experiments for p65 or Stat1 in RAW264.7 cells overexpressing myc-p65 (401-500 aa) and lnc-ip65.

(**f**) Representative immunofluorescent analysis of three independent experiments for the subcellular localization of exogenous/endogenous p65 and lnc-ip65 in RAW264.7 cells (-i) and related statistical analysis (-ii). Scale bars, 25 μm. Arrows point to the cytoplasmic distribution, and pounds to the nucleus distribution.

Data are shown as the mean ± SEM, and are representative of three independent experiments. Analysis is performed using one-way ANOVA (Dunnett's multiple comparisons test). The exact *p* values and sample numbers are shown in the figure or figure legends. * $p < 0.05$, ** $p < 0.01$, *** $p < 0.001$. Molecular weight markers are shown to the left of the blots in kDa, and antibodies used are indicated to the right. Source data are provided as a Source Data file.

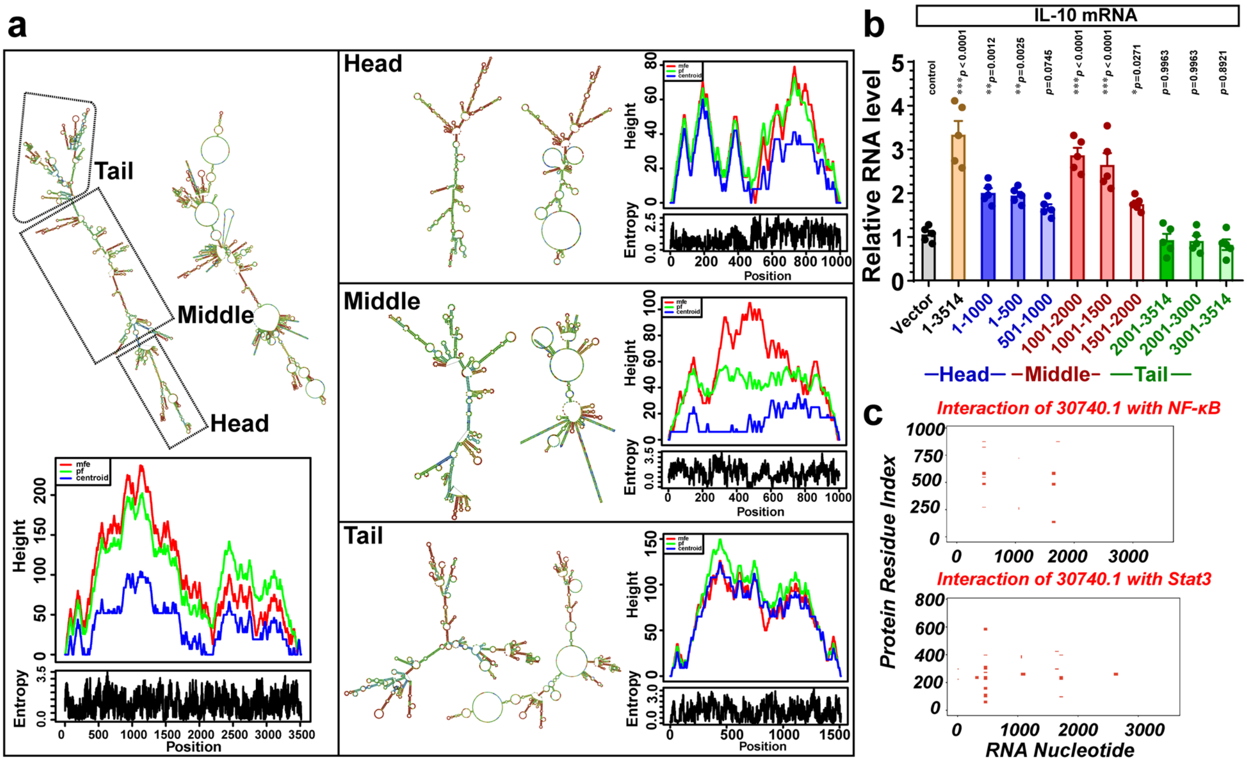

Suppl. Fig.17: Structure and Function of Lnc-ip65, Related to Fig. 7

(**a**) Secondary structure of lnc-ip65 predicted with RNAfold and the related mutant construction. The minimum free energy (MFE) secondary structure, centroid secondary structure, and a mountain plot representation of MFE structure, the thermodynamic ensemble of RNA structures, and the centroid structure are shown.

(**b**) qRT-PCR analysis (n=5) for IL-10 mRNA expression from Fig.7h.

(**c**) Potential interaction relationship of lncRNA 30740.1 with key TFs predicted by catRAPID omics.

Data are shown as the mean ± SEM, and are representative of three independent experiments. Target gene mRNA is normalized to GAPDH mRNA. Analysis is performed using one-way ANOVA (Dunnett's multiple comparisons test). The exact *p* values and sample numbers are shown in the figure or figure legends. * $p < 0.05$, ** $p < 0.01$, *** $p < 0.001$. Source data are provided as a Source Data file.

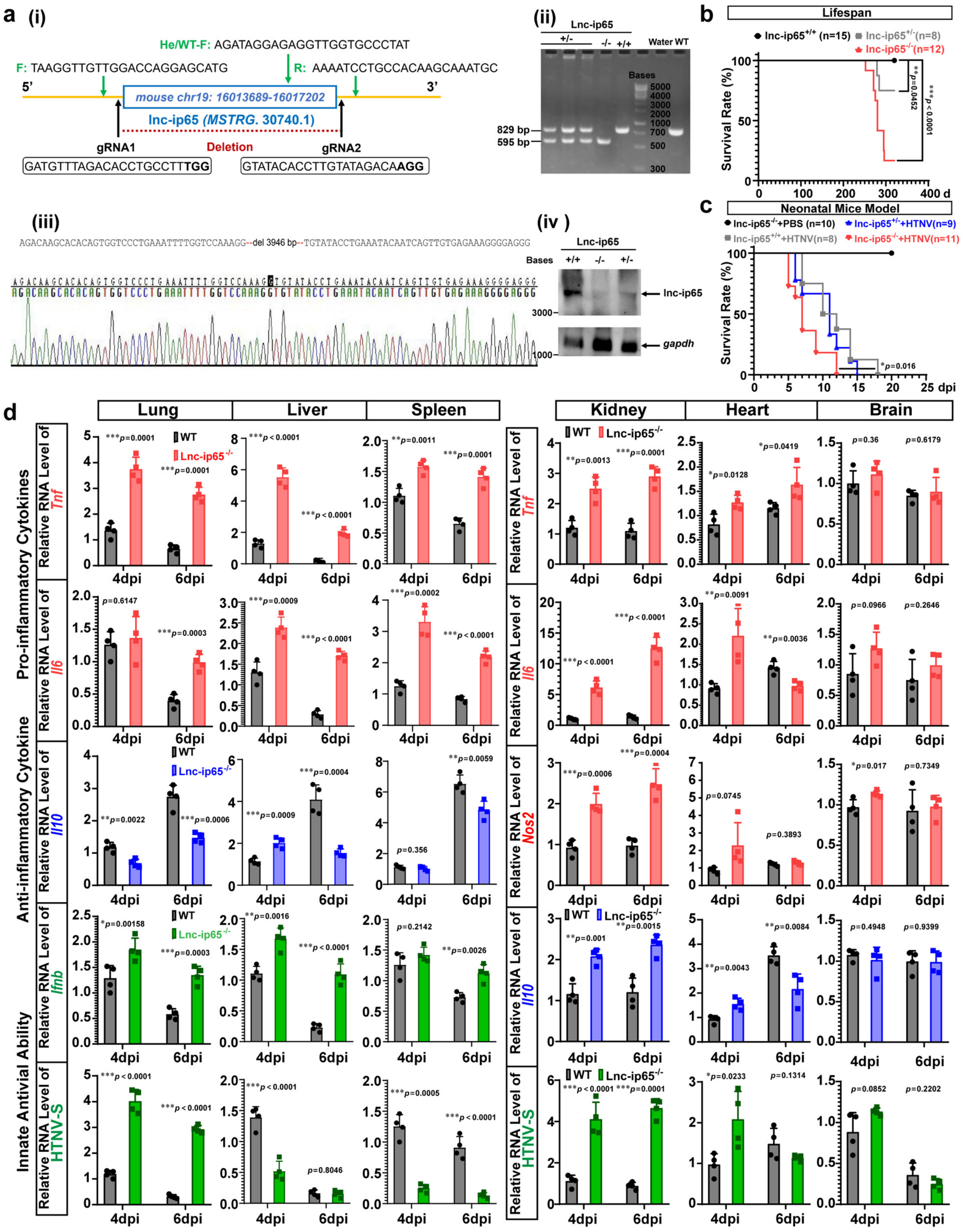
a (i)
He/WT-F: AGATAGGAGAGGTTGGTGCCCTAT
F: TAAGGTTGTTGGACCAGGAGCATG
R: AAAATCCTGCCACAAGCAAATGC
5'
3'
mouse chr19: 16013689-16017202
lnc-ip65 (MSTRG. 30740.1)
gRNA1
Deletion
gRNA2
GATGTTTAGACACCTGCCTTTGG
GTATACACCTTGTATAGACAAGG
(ii)
Lnc-ip65
+/-
-/-
+/+
Water WT
Bases
829 bp
595 bp
b
Lifespan
lnc-ip65+/+ (n=15)
lnc-ip65+/-(n=8)
lnc-ip65-/-(n=12)
Survival Rate (%)
**p=0.0452
***p<0.0001
c
Neonatal Mice Model
lnc-ip65-/-+PBS (n=10)
lnc-ip65+/-+HTNV(n=9)
lnc-ip65+/+ +HTNV(n=8)
lnc-ip65-/-+HTNV(n=11)
*p=0.016
dpi
(iii)
AGACAAGCACACAGTGGTCCCTGAAATTTTGGTCCAAAGG--del 3946 bp--TGTATACCTGAAATACAATCAGTTGTGAGAAAGGGGAGGG
(iv)
Lnc-ip65
Bases
+/+
-/-
+/-
lnc-ip65
gapdh
d
Lung
Liver
Spleen
Kidney
Heart
Brain
Pro-inflammatory Cytokines
Anti-inflammatory Cytokine
Innate Antiviral Ability
Relative RNA Level of Tnf
Relative RNA Level of Il6
Relative RNA Level of Il10
Relative RNA Level of Ifnb
Relative RNA Level of HTNV-S
Relative RNA Level of Nos2
WT
Lnc-ip65-/-
4dpi
6dpi

Suppl. Fig.18: Deteriorated Inflammation in HTNV-infected Lnc-ip65$^{-/-}$ Mice, Related to Fig.8

(**a**) Knockout design with CRISPR/Cas9 technology (-i). PCR (-ii), DNA-Seq (-iii) and Northern blot (-iv) analysis to verify the lncRNA knockout efficacy.

(**b**) Lifespan data shown by survival curve for the WT and transgenic mice.

(**c**) Survival data of the HTNV-challenged lethal neonatal mouse model.

(**d**) qRT-PCR analysis of the indicated genes in the multiple tissues from Fig.8a (n=4).

Data are shown as the mean ± SD, and are representative of two independent experiments. Target gene mRNA is normalized to GAPDH mRNA. Analysis is performed mainly with survival curve comparison (**b** and **c**, log-rank [Mantel–Cox] test), or two-sided unpaired Student's *t* test (**d**). The exact *p* values and sample numbers are shown in the figure or figure legends. * $p < 0.05$, ** $p < 0.01$, *** $p < 0.001$. DNA or RNA markers are shown to the blots in Bases, and target DNA segments or indicated RNA probes used are also marked. Source data are provided as a Source Data file.

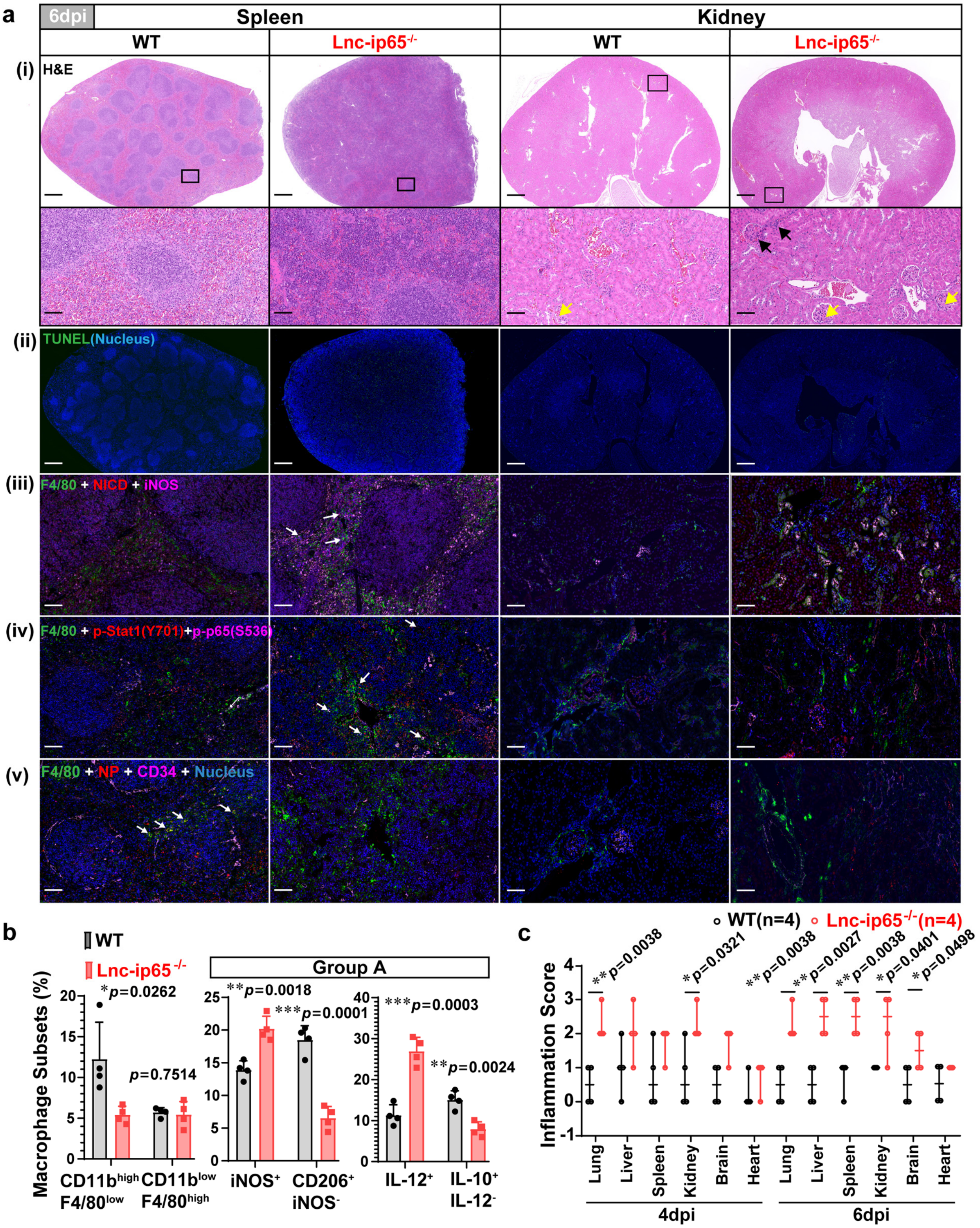
a
6dpi
Spleen
Kidney
WT
Lnc-ip65-/-
WT
Lnc-ip65-/-
(i) H&E
(ii) TUNEL(Nucleus)
(iii) F4/80 + NICD + iNOS
(iv) F4/80 + p-Stat1(Y701)+p-p65(S536)
(v) F4/80 + NP + CD34 + Nucleus
b
WT
Lnc-ip65 -/-
Group A
Macrophage Subsets (%)
*p=0.0262
p=0.7514
**p=0.0018
***p=0.0001
***p=0.0003
**p=0.0024
CD11bhigh F4/80low
CD11blow F4/80high
iNOS+
CD206+ iNOS-
IL-12+
IL-10+ IL-12-
c
WT(n=4)
Lnc-ip65-/-(n=4)
Inflammation Score
**p=0.0038
*p=0.0321
**p=0.0038
**p=0.0027
**p=0.0038
*p=0.0401
*p=0.0498
Lung
Liver
Spleen
Kidney
Brain
Heart
4dpi
6dpi

302

303

Suppl. Fig.19: Aggravated Immunopathologic Injuries in Lnc-ip65$^{-/-}$ Mice Tissues, Related to Fig.8

(**a**) Representative H&E (scale bars, 500 µm for the upper and 50 µm for the bottom) (-i), TUNEL (scale bars, 500 µm) (-ii) and immunofluorescent staining (scale bars, 50 µm) (-iii to -v) of the spleen (left) and kidney (right) tissues at 6 dpi of two independent experiments. Black arrows show the infiltrated lymphocytes, and yellow arrows point to the slight hyperplasia of the extracellular matrix (-i). Arrows indicate the F4/80^{+} macrophages with iNOS^{+} NICD^{+}(-iii), p-p65^{+}(-iv) or NP^{+}(-v).

(**b**) Statistical analysis of the flow cytometry assessment in Fig.8e (n=4).

(**c**) Inflammatory score evaluation of various organs from WT and lnc-ip65$^{-/-}$ mice.

Data are shown as the mean ± SD, and are representative of two independent experiments. Analysis is performed mainly with two-sided unpaired Student's *t* test. The exact *p* values and sample numbers are shown in the figure or figure legends. * $p < 0.05$, ** $p < 0.01$, *** $p < 0.001$. Source data are provided as a Source Data file.

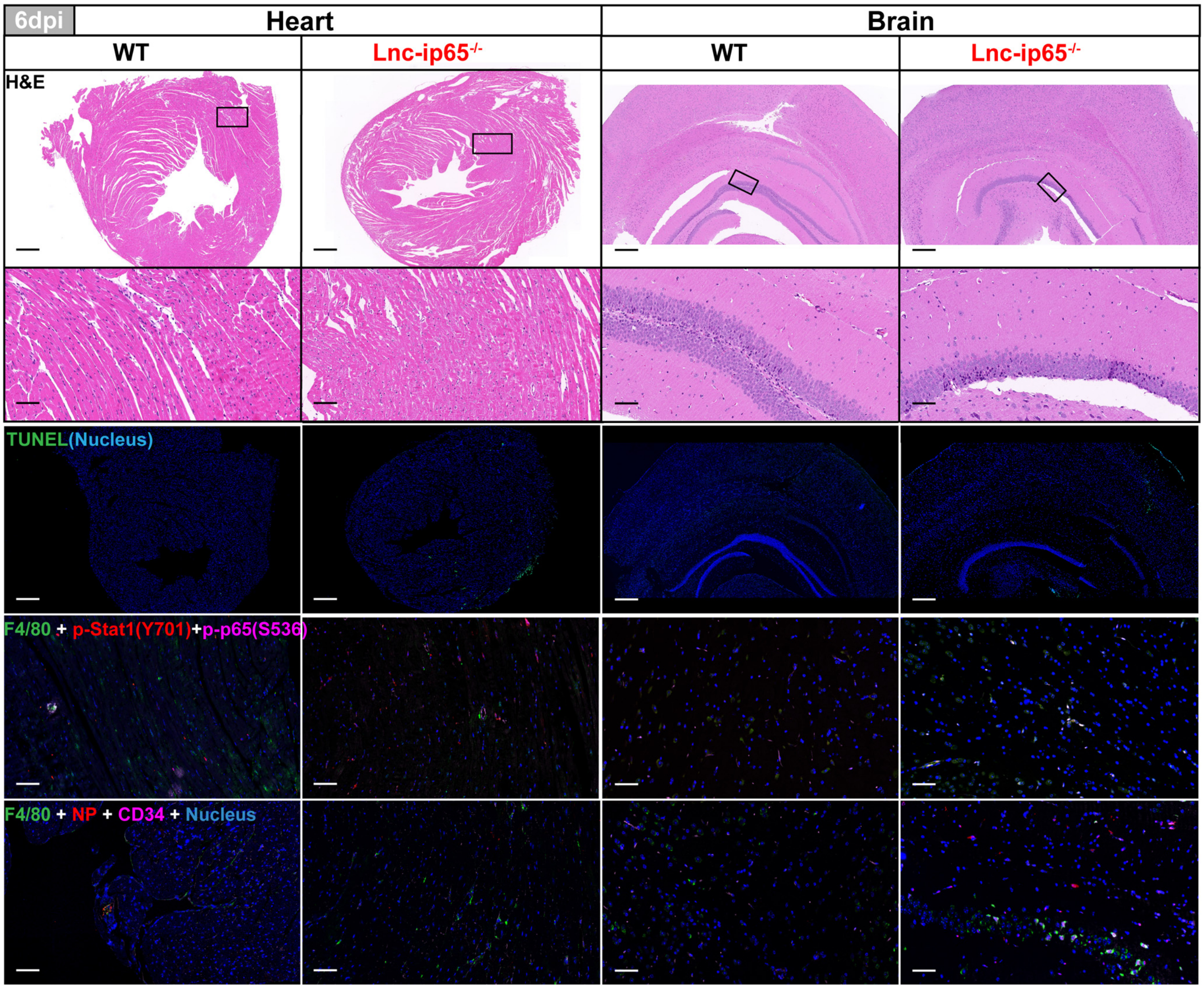

Suppl. Fig.20: Immunopathologic Injuries of the Hearts and Brains from HTNV-infected Lnc-ip65$^{-/-}$ Mice, Related to Fig.8

Representative H&E (scale bars, 500 μm for the upper and 50 μm for the bottom), TUNEL (scale bars, 500 μm) and immunofluorescent staining (scale bars, 50 μm) of the heart (left) and brain (right) tissues of two independent experiments.

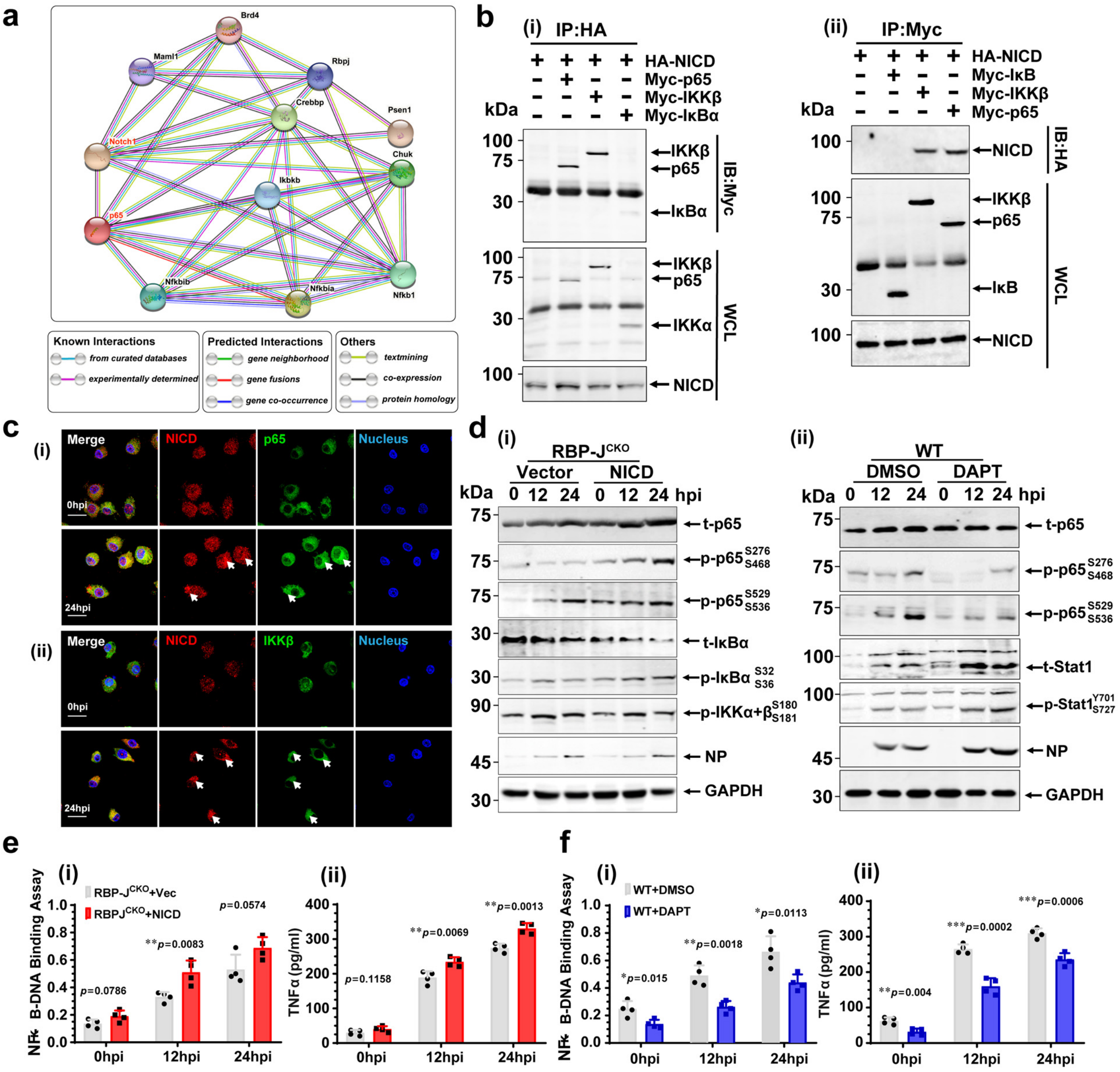
a
Brd4
Maml1
Rbpj
Crebbp
Psen1
Notch1
Chuk
Ikbkb
p65
Nfkbib
Nfkbia
Nfkb1
Known Interactions
from curated databases
experimentally determined
Predicted Interactions
gene neighborhood
gene fusions
gene co-occurrence
Others
textmining
co-expression
protein homology
b
(i) IP:HA
HA-NICD
Myc-p65
Myc-IKKβ
Myc-IκBα
kDa
IKKβ
p65
IκBα
IB:Myc
IKKα
WCL
NICD
(ii) IP:Myc
HA-NICD
Myc-IκB
Myc-IKKβ
Myc-p65
NICD
IB:HA
IKKβ
p65
IκB
WCL
c
(i)
Merge
NICD
p65
Nucleus
0hpi
24hpi
(ii)
IKKβ
d
(i) RBP-J^CKO
Vector
NICD
hpi
t-p65
p-p65 S276 S468
p-p65 S529 S536
t-IκBα
p-IκBα S32 S36
p-IKKα+β S180 S181
NP
GAPDH
(ii) WT
DMSO
DAPT
t-Stat1
p-Stat1 Y701 S727
e
(i)
RBP-J^CKO+Vec
RBPJ^CKO+NICD
NFκB-DNA Binding Assay
p=0.0786
**p=0.0083
p=0.0574
0hpi
12hpi
24hpi
(ii)
TNFα (pg/ml)
p=0.1158
**p=0.0069
**p=0.0013
f
(i)
WT+DMSO
WT+DAPT
*p=0.015
**p=0.0018
*p=0.0113
(ii)
**p=0.004
***p=0.0002
***p=0.0006

Suppl. Fig.21: NICD Promotes p65-mediated M1 Activation during HTNV Infection

(**a**) The crosstalk between the Notch and NF-κB pathways based on the STRING database.

(**b**) Representative coimmunoprecipitation (co-IP) analysis of three independent experiments for the interaction between HA-NICD and myc-tagged NF-κB-related proteins in NIH/3T3 cells. Anti-HA (-i) or anti-myc (-ii) antibodies are used to pull down myc-tagged p65/IKKβ/IκBα or HA-tagged NICD, respectively.

(**c**) Representative immunofluorescent assays of three independent experiments for NICD with p65 (-i) or IKKβ (-ii) in HTNV-infected mBMDM (MOI=1).

(**d**) Representative immunoblot analysis of three independent experiments for the NF-κB in RBP-J^{CKO} mBMDM expressing NICD (i, MOI=0.5) or pretreated with DAPT (50 μmol/L) (-ii, MOI=5).

(**e**) DNA-binding ability (-i) and TNFα production (-ii) for mBMDM from (**d**-i) (n=4).

(**f**) DNA-binding ability (-i) and TNFα production (-ii) for mBMDM from (**d** -ii) (n=4).

Data are shown as the mean ± SEM, and are representative of three independent experiments. Analysis is performed mainly with two-sided unpaired Student's *t* test. The exact *p* values and sample numbers are shown in the figure or figure legends. * $p < 0.05$, ** $p < 0.01$, *** $p < 0.001$. Molecular weight markers are shown to the left of the blots in kDa, and antibodies used are indicated to the right. Source data are provided as a Source Data file.

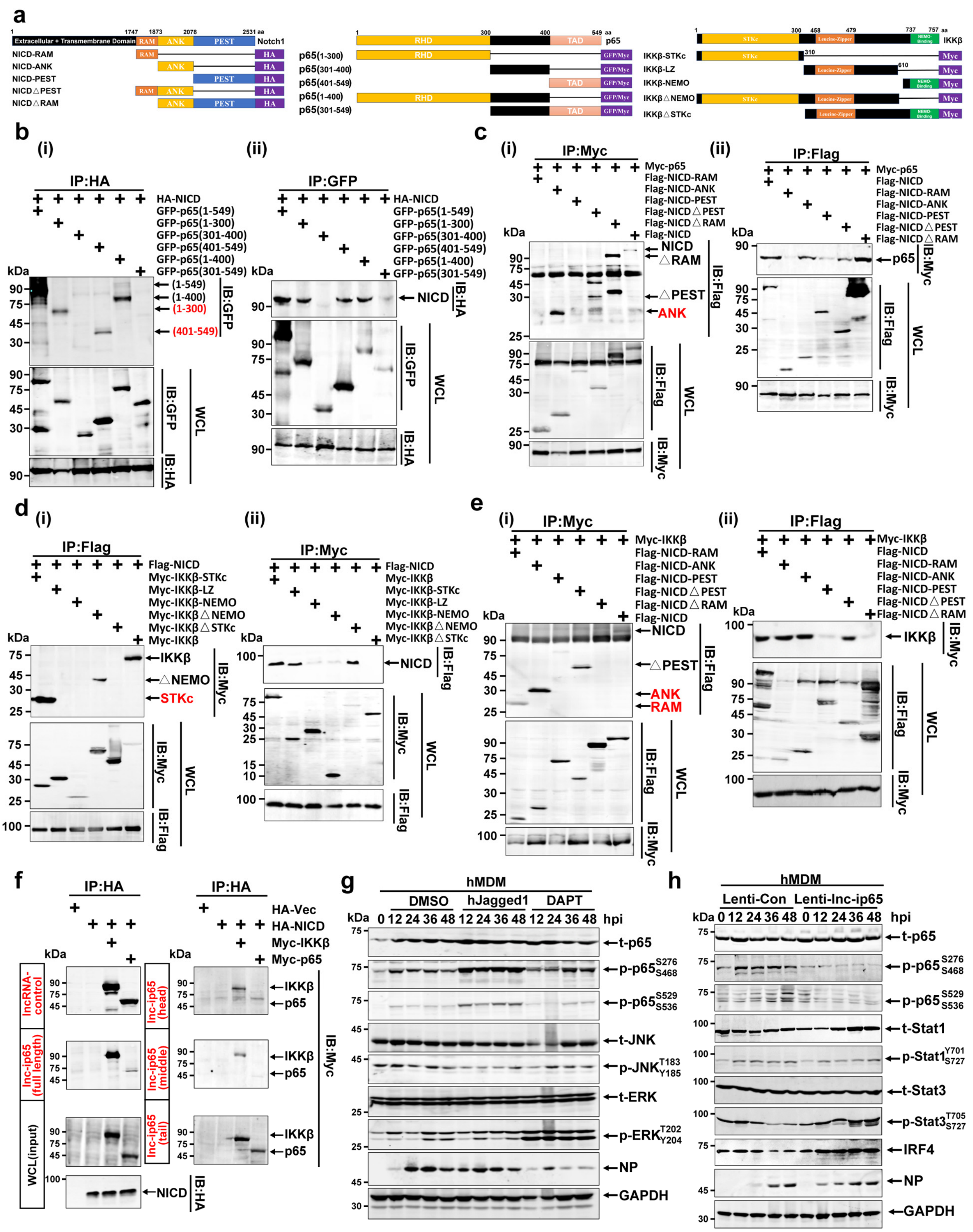
a
Extracellular + Transmembrane Domain
RAM
ANK
PEST
HA
Notch1
NICD-RAM
NICD-ANK
NICD-PEST
NICD△PEST
NICD△RAM
RHD
TAD
p65
GFP/Myc
p65(1-300)
p65(301-400)
p65(401-549)
p65(1-400)
p65(301-549)
STKc
Leucine-Zipper
NEMO-Binding
IKKβ
Myc
IKKβ-STKc
IKKβ-LZ
IKKβ-NEMO
IKKβ△NEMO
IKKβ△STKc
b
(i)
IP:HA
HA-NICD
GFP-p65(1-549)
GFP-p65(1-300)
GFP-p65(301-400)
GFP-p65(401-549)
GFP-p65(1-400)
GFP-p65(301-549)
kDa
IB:GFP
IB:HA
WCL
(ii)
IP:GFP
NICD
c
IP:Myc
Myc-p65
Flag-NICD-RAM
Flag-NICD-ANK
Flag-NICD-PEST
Flag-NICD△PEST
Flag-NICD△RAM
Flag-NICD
IB:Flag
IB:Myc
IP:Flag
d
Flag-NICD
Myc-IKKβ-STKc
Myc-IKKβ-LZ
Myc-IKKβ-NEMO
Myc-IKKβ△NEMO
Myc-IKKβ△STKc
Myc-IKKβ
e
Myc-IKKβ
f
HA-Vec
HA-NICD
Myc-IKKβ
Myc-p65
lncRNA-control
lnc-ip65 (full length)
WCL(input)
lnc-ip65 (head)
lnc-ip65 (middle)
lnc-ip65 (tail)
g
hMDM
DMSO
hJagged1
DAPT
hpi
t-p65
p-p65 S276 S468
p-p65 S529 S536
t-JNK
p-JNK T183 Y185
t-ERK
p-ERK T202 Y204
NP
GAPDH
h
Lenti-Con
Lenti-lnc-ip65
t-Stat1
p-Stat1 Y701 S727
t-Stat3
p-Stat3 T705 S727
IRF4

Suppl. Fig.22: NICD Activates NF-κB Pathway by Recruiting p65 and IKKβ, Which Is Restrained by Lnc-ip65

(**a**) Schematic diagram for constructing truncated NICD, p65 or IKKβ.

(**b**) Representative co-IP analysis of three independent experiments for the interaction between HA-NICD and truncated GFP-tagged p65 in NIH/3T3 cells. Anti-HA (-i) or anti-GFP (-ii) antibodies are used to pull down GPF-tagged p65 mutants or HA-NICD, respectively.

(**c**) Representative co-IP analysis of three independent experiments for the interaction between myc-p65 and truncated flag-tagged NICD by in NIH/3T3 cells. Anti-myc (-i) or anti-flag (-ii) antibodies are used to pull down flag-tagged NICD mutants or myc-p65, respectively.

(**d**) Representative co-IP analysis of three independent experiments for the interaction between flag-NICD and truncated myc-tagged IKKβ in NIH/3T3 cells. Anti-flag (-i) or anti-myc (-ii) antibodies are used to pull down myc-tagged IKKβ mutants or flag-NICD, respectively.

(**e**) Representative co-IP analysis of three independent experiments for the interaction between myc-IKKβ and truncated flag-tagged NICD in NIH/3T3 cells. Anti-myc (-i) or anti-flag (-ii) antibodies are used to pull down flag-tagged NICD mutants or myc-IKKβ, respectively.

(**f**) Representative co-IP analysis of three independent experiments for the interaction between HA-NICD and myc-p65 or myc-IKKβ in NIH/3T3 cells that are co-transfected with vectors transcribing lnc-ip65 and its mutants.

(**g**) Representative immunoblot analysis of three independent experiments for the phosphorylation of p65, JNK or ERK in hMDM (MOI=5). Cells are pre-treated with hJagged1 (25 ng/ml) or DAPT (15 μmol/L) for 24 hr before HTNV infection.

(**h**) Representative immunoblot analysis of three independent experiments for the activation of M1- or M2-related TFs in hMDM exogenously expressing lnc-ip65 (MOI=5).

Data are representative of three independent experiments. Molecular weight markers are shown to the left of the blots in kDa, and antibodies used are indicated to the right. Source data are provided as a Source Data file.

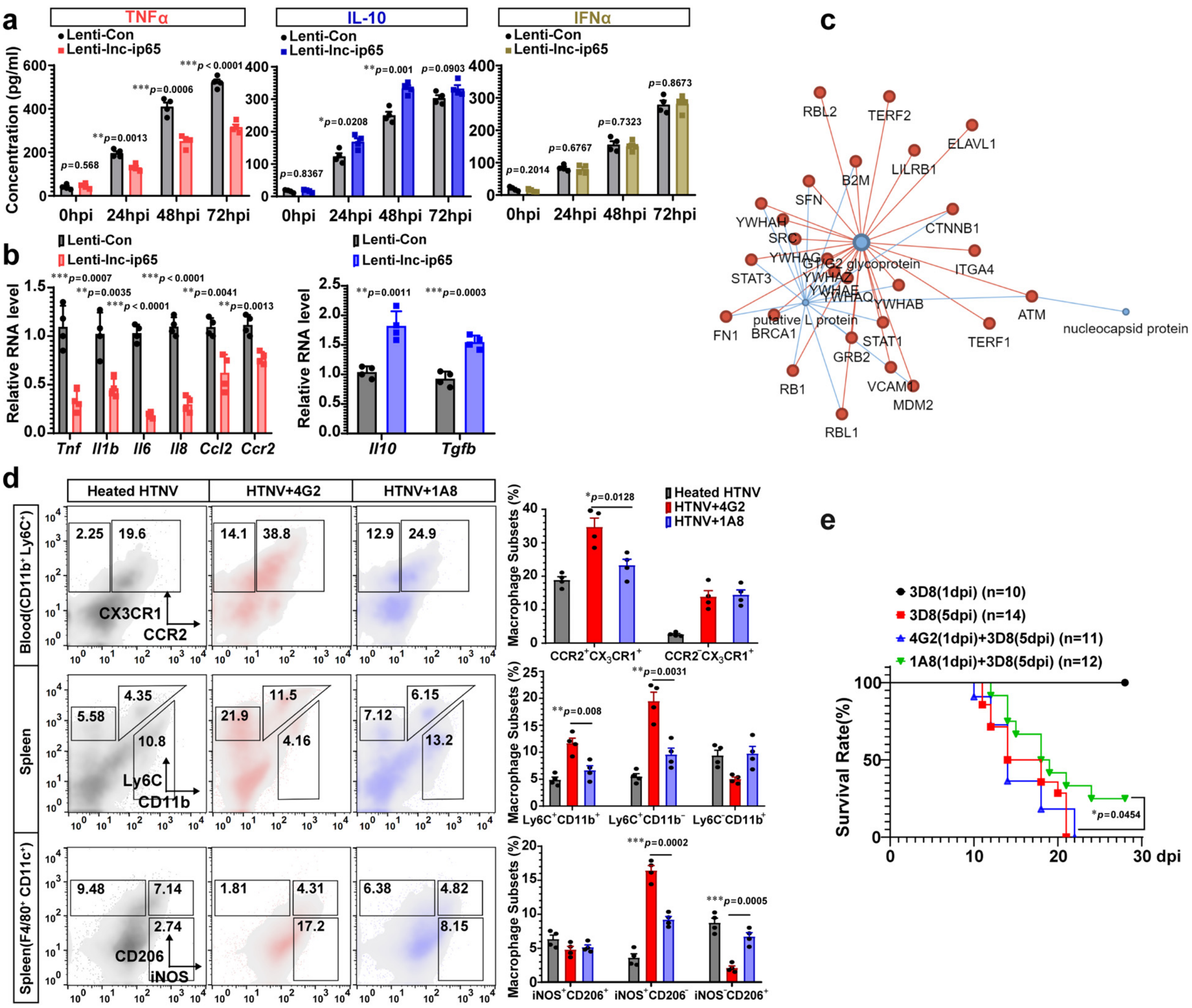

Suppl. Fig.23: Lnc-ip65 Suppresses Human Inflammatory Macrophage Polarization, Related to Fig.9

(**a** and **b**) Supernatant cytokine concentration detected by ELISA (**a**), and qRT-PCR analysis of the pro- and anti-inflammatory gene expression (**b**) for hMDM from Suppl. Fig.23h (n=4).

(**c**) The potential interaction of hantaviral proteins with host factors predicated with P-HIPSTer.

(**d**) Representative flow cytometry analysis of two independent experiments for the activation of MPS in neonatal mice at 3 dpi from Fig.9e (n=4).

(**e**) Survival data for 4-day-old neonatal mice challenged with HTNV (i.p., 8×10^5 $TCID_{50}$/g). Treatments, 3D8 (0.05 μg/g) at 1 or 5 dpi, or 3D8 (0.05 μg/g) combined with 4G2 (0.25 μg/g) or 1A8 (0.25 μg/g) at 5 dpi (every two days).

Data are shown as the mean ± SEM, and are representative of two independent experiments. Analysis is performed mainly with two-sided unpaired Student's *t* test (**a-d**), or the survival curve comparison (**e**, log-rank [Mantel–Cox] test). The exact *p* values and sample numbers are shown in the figure or figure legends. * $p < 0.05$, ** $p < 0.01$, *** $p < 0.001$. Source data are provided as a Source Data file.

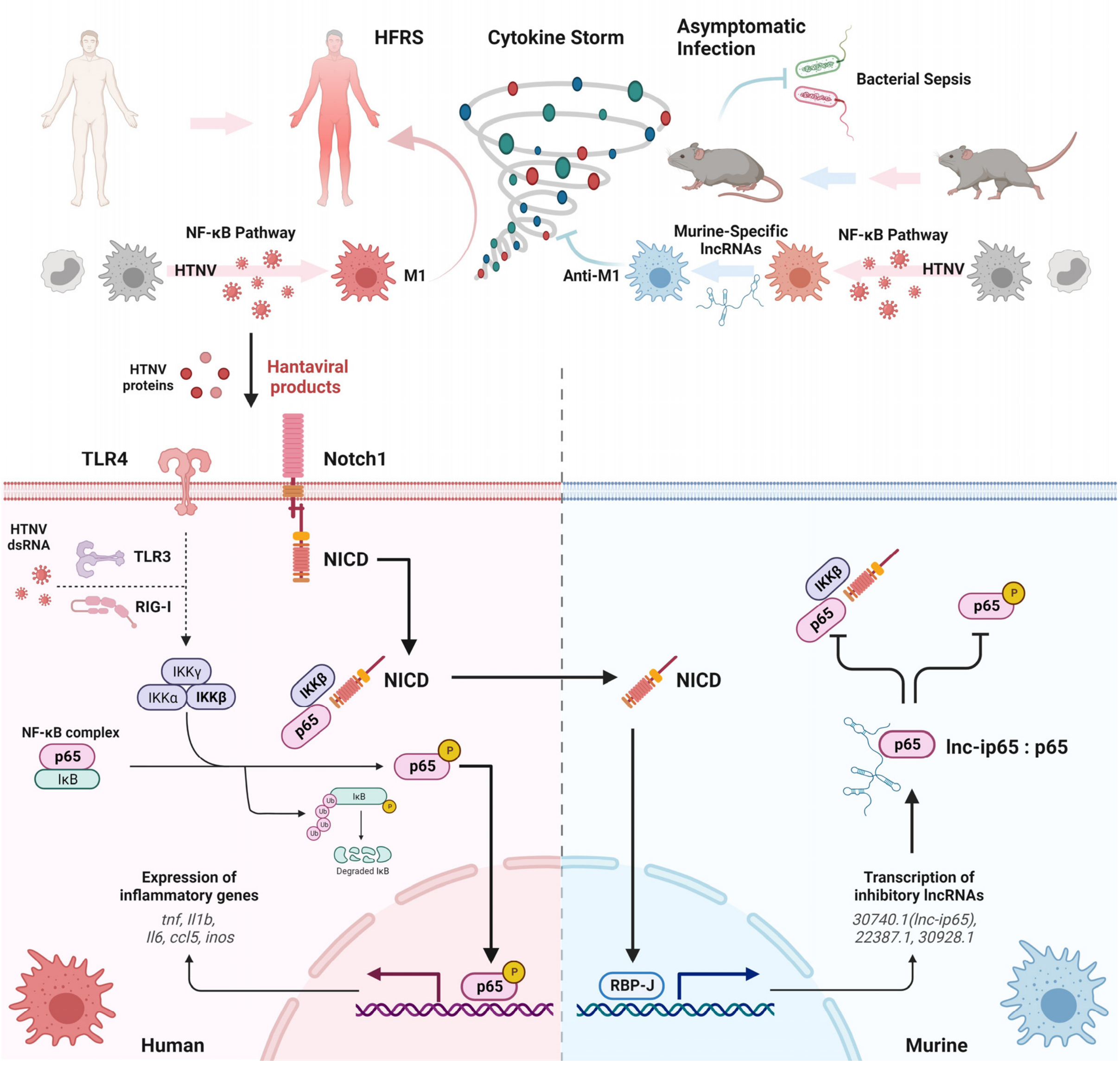
HFRS
Cytokine Storm
Asymptomatic Infection
Bacterial Sepsis
NF-κB Pathway
HTNV
M1
Anti-M1
Murine-Specific lncRNAs
NF-κB Pathway
HTNV
HTNV proteins
Hantaviral products
TLR4
Notch1
HTNV dsRNA
TLR3
RIG-I
NICD
IKKγ
IKKα
IKKβ
IKKβ
p65
NICD
NICD
NF-κB complex
p65
IκB
p65
P
IκB
Ub
Degraded IκB
Expression of inflammatory genes
tnf, Il1b, Il6, ccl5, inos
p65
RBP-J
IKKβ
p65
p65
P
p65
lnc-ip65 : p65
Transcription of inhibitory lncRNAs
30740.1(lnc-ip65), 22387.1, 30928.1
Human
Murine

Suppl. Fig.24: Working Model Deciphering the Differential Macrophage Phenotype Rewired by Hantaan Virus in Murine versus Humans

In brief, this study demonstrates that a unique anti-inflammatory phenotype of macrophage at the late infection phase is reprogrammed by HTNV through Notch-lncRNA-p65 pathway in mice versus humans, which might interpret the discrepant disease outcomes of natural reservoirs or HFRS patients. Hyperactivation of inflammatory macrophage (M1) launches the TNFα-centered cytokine storm in human beings and correlates with the disease severity of HFRS. The unique anti-M1 status of murine macrophage at late infection stage protects mice from HTNV challenge and subsequent bacterial sepsis. HTNV NP facilitates Notch-mediated M1 activation and exacerbates disease progression, while murine-specific lnc-ip65 downstream of the Notch pathway contributes to the late-phase inactivation of M1. Lnc-ip65 interacts with p65 and inhibits its phosphorylation, the deficiency of which would aggravate systemic inflammation and sensitize mice to HTNV infection. The graphical abstract was created with BioRender.com (Agreement number: YB2689PDFA).

Suppl. Table 1: The incidence and mortality rate of HFRS in China, Related to Fig.2

Regions	2016				2017			
	Reported Cases	Incidence Rate	Death Toll	Mortality Rate	Reported Cases	Incidence Rate	Death Toll	Mortality Rate
*Shaanxi	933	2.4599	1	0.0026	2064	5.4129	14	0.0367
Heilongjiang	1200	3.148	2	0.0052	1119	2.9455	2	0.0053
Liaoning	863	1.9692	3	0.0068	1078	2.4624	3	0.0069
Jilin	515	1.8705	2	0.0073	574	2.1003	3	0.011
Shandong	985	1.0003	12	0.0122	1252	1.2587	10	0.0101
Jiangxi	676	1.4806	5	0.011	571	1.2434	1	0.0022
Fujian	366	0.9534	0	-	388	1.0015	1	0.0026
Hunan	588	0.8678	3	0.0044	632	0.9264	4	0.0059
Hubei	236	0.4033	5	0.0085	505	0.8581	6	0.0102
Zhejiang	345	0.6229	0	-	351	0.6279	1	0.0018
Hebei	434	0.5845	1	0.0013	447	0.5983	0	-
Yunnan	216	0.4555	0	-	229	0.48	0	-
Jiangsu	330	0.4137	8	0.01	375	0.4688	3	0.0038
Henan	176	0.1857	1	0.0011	435	0.4563	11	0.0115
Anhui	207	0.3369	0	-	273	0.4406	2	0.0032
Guangdong	410	0.3779	3	0.0028	443	0.4028	0	-
Inner Mongoria	106	0.4221	1	0.004	93	0.369	0	-
Sichuan	110	0.1341	1	0.0012	260	0.3147	1	0.0012
Tianjin	31	0.2004	0	-	32	0.2048	0	-
Guizhou	59	0.1672	0	-	58	0.1632	0	-
Gansu	19	0.0731	0	-	20	0.0766	0	-
Shanxi	8	0.0218	0	-	26	0.0706	2	0.0054
Hainan	3	0.0329	0	-	3	0.0327	0	-
Beijing	9	0.0415	0	-	7	0.0322	0	-
Tibet	1	0.0309	0	-	1	0.0302	0	-
Ningxia	4	0.0599	0	-	2	0.0296	0	-
Guangxi	5	0.0104	0	-	14	0.0289	0	-
Chongqing	13	0.0431	0	-	5	0.0164	0	-
Xinjiang	1	0.0042	0	-	3	0.0125	0	-
Shanghai	4	0.0166	0	-	2	0.0083	0	-
Taiwan	4	0.0056	0	-	0	-	0	-
Total	8853	0.6458	48	0.0035	11262	0.8162	64	0.0046

* *A. agrarius* mice were captured in the field of Hu County in the Weihe Plain (106–110°E, 34–36°N, Shaanxi Province, in which there was the highest incidence rate of HFRS in China (2017). The epidemiological data of HFRS in China were acquired from the Chinese Center for Disease Control and Prevention (CDCD) (Available at: http://www.stats.gov.cn/).

Suppl. Table 2: RNA Sequences for RNAi or RNA Detection in This Study

**Antisencse LNA™ GapmeR Standard for RNAi**	
lncRNA MSTRG-22387.1	TGTGCGGGCAAGGAGG
lncRNA MSTRG-30928.1-1	GTAGCCAACAGATGAG
lncRNA MSTRG-30928.1-2	AAAGTAGCCAACAGAT
lncRNA MSTRG-30740.1-1	GCAGAACTTTGACGGA
lncRNA MSTRG-30740.1-2	TGACATAACCTCTTTA
lncRNA MSTRG-30740.1-3	GTAACAGAAGGTGGAC
lncRNA MSTRG-30740.1-4	GGGTGGGTGTCAATAA
lncRNA MSTRG-30740.1-10	AATTTGGAGTAAGAGT
**Biotin-labeled Probes for Northern Blot**	
lncRNA MSTRG-22387.1	5'-BiosG/TCGCGCAGTTCGCACTCA/Bio-3'
lncRNA MSTRG-30740.1	5'-BiosG/TCTGGATGCTGCACAAATGAGT/Bio-3'
lncRNA MSTRG-30928.1	5'-BiosG/TAGCTTACATGCCAGTCACTT/Bio-3'
**SiRNA Targeting Sequences for RNAi of Murine LncRNAs or Genes**	
lncRNA MSTRG-22387.1-1	GGGAAACCATTACATAAGC
lncRNA MSTRG-22387.1-2	AAACCATTACATAAGCCCG
lncRNA MSTRG-22387.1-3	CCATTACATAAGCCCGCGC
lncRNA MSTRG-30740.1-1	CGTTCTTATTGACAGTATA
lncRNA MSTRG-30740.1-2	GGTTAAAGAGGTTATGTCA
lncRNA MSTRG-30740.1-3	CAAGATATAAGCAGCTTAA
lncRNA MSTRG-30928.1-1	CACAAGATACCAAAGTTTA
lncRNA MSTRG-30928.1-2	GGCTACTTTCAATATTCTA
lncRNA MSTRG-30928.1-3	GGAAAGTCGTACCTCCTAA
lncRNA MSTRG-60100.1-1	GGCACACTTTCAGTAAATGTACC
lncRNA MSTRG-60100.1-2	ATGATGGTTACCTTTTTCACTGT
lncRNA MSTRG-60100.1-3	CCTGAAACTGCGAGACAAATAA
lncRNA MSTRG-59654.1-1	GAGGAAATGTTTAGTAAAGCAAA
lncRNA MSTRG-59654.1-2	ATGTCTTTGTGCCCAAACTCACA
lncRNA MSTRG-57001.1-1	CACAGTCTATTCTGTGTATATGT
lncRNA MSTRG-57001.1-2	GGCACACTATGCAAGAACTTAAC
lncRNA MSTRG-61517.1-1	GGGAGAAAACACATTATCACAAT
lncRNA MSTRG-61517.1-2	CACTGACAAAATAATTATGCATC
lncRNA MSTRG-61517.1-3	GAGTTTACAATTAAACTATTATC
lncRNA MSTRG-11443.1-1	TGCTACATTTGACAGTTTTCTTC
lncRNA MSTRG-11443.1-2	ATGGAACAAAAGCAATTAAATAG
TLR3-1	CTGAAAGGAAATGTGTGATTTAA
TLR3-2	CAGGATTTAACTCCATTTCAAAA
TLR3-3	TTCACAAGTTTGCAAACTTTAAC
TLR4-1	GTGGATCTTTCTTATAACTATAT
TLR4-2	GTGTATCTTTGAATATGAGATTG
TLR4-3	ATCATGATTTCATCATTTATTCT
RIG-I-1	TTCTGAACTATCCGAATGTTTGA
RIG-I-2	GGCCATTTCCCGTCTTTGAATAG
RIG-I-3	TTGTAAACATTACAATAATTTAA
MDA5-1	GAGTGCAATATTGTTATTCGTTA
MDA5-2	ATGCATCATGTCAATATGACACC
MDA5-3	CTCATAACTTTGCACTTCATTGT

Suppl. Table 3: PCR Primers Applied in This Study

**Murine Macrophage Activation Related Genes**	
Mus-TNF-α F	GCCTCTTCTCATTCCTGCTT
Mus-TNF-α R	CTCCTCCACTTGGTGGTTTG
Mus-IL-6 F	TGCAAGAGACTTCCATCCAGTTG
Mus-IL-6 R	TAAGCCTCCGACTTGTGAAGTGGT
Mus-MCP-1 F	TTCTTCGATTTGGGTCTCCTTG
Mus-MCP-1 R	GTGCAGCTCTTGTCGGTGAA
Mus-iNOS F	ACAACAGGAACCTACCAGCTCA
Mus-iNOS R	GATGTTGTAGCGCTGTGTGTCA
Mus-IL-10 F	TGAATTCCCTGGGTGAGAAGCTGA
Mus-IL-10 R	TGGCCTTGTAGACACCTTGGTCTT
Mus-Arg1 F	TCCTTAGAGATTATCGGAGCGCCT
Mus-Arg1 R	TTTCCAGCAGACCAGCTTTCCTCA
Mus-YM-1 F	CATTCAGTCAGTTATCAGATTCC
Mus-YM-1 R	AGTGAGTAGCAGCCTTGG
Mus-TGF-β F	TACCATGCCAACTTCTGTCTGGGA
Mus-TGF-β R	TGTGTTGGTTGTAGAGGGCAAGGA
Mus-GAPDH F	CCTCGTCCCGTAGACAAAATG
Mus-GAPDH R	TGAGGTCAATGAAGGGGTCGT
**Murine Notch Pathway Relate Genes**	
Mus-Notch1 F	TGCCAGGACCGTGACAACTC
Mus-Notch1 R	CACAGGCACATTCGTAGCCATC
Mus-Notch2 F	GAGAAAAACCGCTGTCAGAATGG
Mus-Notch2 R	GGTGGAGTATTGGCAGTCCTC3'
Mus-Notch3 F	AGTGCCGATCTGGTACAACTT
Mus-Notch3 R	CACTACGGGGTTCTCACACA
Mus-Jagged1 F	AAAGGCTTCACCGGCACCTAC
Mus-Jagged1 R	CTCCCAGCCGTCACTACAGATACA
Mus-Jagged2 F	GCGACCAGTACGGCAACAA
Mus-Jagged2 R	CCGTGGAGCAAATTACATCCTT
Mus-Hes1 F	AAAGACGGCCTCTGAGCAC
Mus-Hes1 R	GGTGCTTCACAGTCATTTCCA
Mus-Hey1 F	CCGACGAGACCGAATCAATAAC
Mus-Hey1 R	TCAGGTGATCCACAGTCATCTG
**Murine-specific LncRNAs Downstream Notch Signaling**	
MSTRG.31351.1F	ACACAGACCACATGGAGGTCAGTC
MSTRG.31351.1R	GCCACCACCATGATGCGGATG
MSTRG.31038.1F	CTGACACGCCCAGGTTCCTA
MSTRG.31038.1R	TCTGACCTGGGTTCTCCATGTT
MSTRG.30928.1F	TGTCTGTATCTCATCTGTTGGCT
MSTRG.30928.1R	TCAGTGCTTGGTTCTCTTGTGT
MSTRG.30740.1F	ATGGATTGTGACATCGAGCGGAAG
MSTRG.30740.1R	AGAAGGTGGACGAGGCAGCTAG
MSTRG.30311.1F	GCAGCCTCCACAGCCAACAC
MSTRG.30311.1R	CCACTGTCACACGCAGCCTTAG
MSTRG.26682.1F	AGCTTGTGACTGTGACAATAGCA
MSTRG.26682.1R	TGTTTCAGATTTCTTCCCTCTCTATGC

MSTRG.22387.1F	GACCCTCAGTCCCCTCCTT
MSTRG.22387.1R	GGCGCGGGCTTATGTAATG
MSTRG.21694.1F	GCTTGGTGTGAGGCTGTGAC
MSTRG.21694.1R	ACCACACCCATGCTCTCTGT
MSTRG.17804.1F	TCTGGTGTGTAAGTTGCTTTCCT
MSTRG.17804.1R	ACGGACTCAAGTCTGCTGAG
MSTRG.14108.1F	CTTGTGCGGGATTGGAAGGC
MSTRG.14108.1R	GCCCATGGGTAGAATGGCCA
MSTRG.11443.1F	CACCTCTATGGCCCACACCT
MSTRG.11443.1R	TCTCGGAATAGACGCGGAAGA
MSTRG.9634.1F	TCCATGCAGCACAATGACCT
MSTRG.9634.1R	CTGTGGGTGCCCTGAAGTTG
MSTRG.7244.1F	ATTCGCCTGAGACACGCAAGC
MSTRG.7244.1R	GCTCTAGCACCAAGCTCCATACC
MSTRG.6924.1F	TGCCAAGGGCATTCCAGAAG
MSTRG.6924.1R	AGGTCTGCATCTGGCTTCCA
MSTRG.6898.1F	AGCCCACAAAGTCAGAGCCA
MSTRG.6898.1R	GCCCTGGGTGACTTGTAAGG
MSTRG.61517.1F	AGCCATTGAGGGAAGATGCTG
MSTRG.61517.1R	CACCTCTTTCTGTGTCTTTCCAG
MSTRG.61225.1F	TGGGCTTCATGATGATACTTCCA
MSTRG.61225.1R	ACACATTCACGTTGATGTACAAACT
MSTRG.60100.1F	AGCCTGTGTTCCAGCATCAGTTAC
MSTRG.60100.1R	TTCACTCCGACTTCCAGCTCTCC
MSTRG.59654.1F	CCAACCACTGGGGAGGAAATG
MSTRG.59654.1R	CGGGCTTGGAACTTACTCTCT
MSTRG.57004.1F	TGGCGAACACAAGGTCAGACATAG
MSTRG.57004.1R	TCCTGATGTTGCTGCTCTCACTTC
MSTRG.57001.1F	GGGAATCATAGGCAGTGGGC
MSTRG.57001.1R	GCCAGCGTTCTATCCCAAGC
MSTRG.54891.1F	CCTGCCTAGCGCAAGCTTATGTAG
MSTRG.54891.1R	TACACCTGACAGAAGCCACTCCAG
MSTRG.53376.1F	ACTGGTGGACAGGGAACAGT
MSTRG.53376.1R	GGAGCCACGCCATGTTTCAT
MSTRG.52933.1F	TTGCTGTGATGAGATGCCATGACC
MSTRG.52933.1R	CTTGCCTAGCTGCCTGTTGCC
MSTRG.52051.1F	GGTGACATAGAGCCACTGCAC
MSTRG.52051.1R	GCTGAGCCATGATCACCAACA
MSTRG.51772.1F	CCACCTGCCTCTGCCTCCTG
MSTRG.51772.1R	ATCACCACCACCTGGCTAGATGG
MSTRG.51733.1F	TTGGTTTGTGGTGCTGTGCT
MSTRG.51733.1R	AGTCAACATGGCTGTGCTGG
MSTRG.47857.1F	TGTGTCCTTGGATTCGAGAAGG
MSTRG.47857.1R	TCAGGGCTTCAGTGGGTTCA
MSTRG.43613.1F	GCAAGAGGAAGAAGCAGGTCTAGC
MSTRG.43613.1R	CGTGCGGAGGTCAAGGATTCTG
MSTRG.42109.1F	TGCGGAAGCTGCATGAACCTG
MSTRG.42109.1R	TTGCTGACCAACGTGATGAGTTCC
MSTRG.38296.1F	AGCGGATGGTACAGCCTGAGTC
MSTRG.38296.1R	TGATAAGCGTGCTGTGTAGTGGTG

**Human Notch Pathway Relate Genes**	
Homo-Notch1 F	GAGGCGTGGCAGACTATGC
Homo-Notch1 R	CTTGTACTCCGTCAGCGTGA
Homo-Notch2 F	CAACCGCAATGGAGGCTATG
Homo-Notch2 R	GCGAAGGCACAATCATCAATGTT
Homo-Notch3 F	TGGCGACCTCACTTACGACT
Homo-Notch3 R	CACTGGCAGTTATAGGTGTTGAC
Homo-Notch4 F	TGTGAACGTGATGTCAACGAG
Homo-Notch4 R	ACAGTCTGGGCCTATGAAACC
Homo-Dll1 F	GATTCTCCTGATGACCTCGCA
Homo-Dll1 R	TCCGTAGTAGTGTTCGTCACA
Homo-Dll3 F	CACTCCCGGATGCACTCAAC
Homo-Dll3 R	GATTCCAATCTACGGACGAGC
Homo-Dll4 F	GTCTCCACGCCGGTATTGG
Homo-Dll4 R	CAGGTGAAATTGAAGGGCAGT
Homo-Jagged1 F	GTCCATGCAGAACGTGAACG
Homo-Jagged1 R	GCGGGACTGATACTCCTTGA
Homo-Jagged2 F	TGGGCGGCAACTCCTTCTA
Homo-Jagged2 R	GCCTCCACGATGAGGGTAAA
Homo-Hes1 F	TCAACACGACACCGGATAAAC
Homo-Hes1 R	GCCGCGAGCTATCTTTCTTCA
Homo-Hey1 F	GTTCGGCTCTAGGTTCCATGT
Homo-Hey1 R	CGTCGGCGCTTCTCAATTATTC
Homo-GAPDH F	GGAGCGAGATCCCTCCAAAAT
Homo-GAPDH R	GGCTGTTGTCATACTTCTCATGG
**Positive Controls for RIP Experiments**	
U1 snRNA F	GGGAGATACCATGATCACGAAGGT
U1 snRNA R	CCACAAATTATGCAGTCGAGTTTCCC

**Transgenic Mice Identification PCR Primers**		
Lyz2-Cre	Cre F	CCGGTCGATGCAACGAGTGATGAGG
	Cre R	GCCTCCAGCTTGCATGATCTCCGG
RBP^{CKO}	RBP-J-F	GTTCTTAACCTGTTGGTCGGAACC
	PGK-D1	ACCGGTGGATGTGGAATGTGT
	RBP-J-R	GCTTGAGGCTTGATGTTCTGTATTGC
$NICD^{STOP\text{-}floxed}$	N1	TAAGCCTGCCCAGAAGACTC
	N2	GAAAGACCGCGAAGAGTTTG
	N3	AAAGTCGCTCTGAGTTGTTAT
Lnc-ip65$^{-/-}$	30740.1-F	TAAGGTTGTTGGACCAGGAGCATG
	30740.1-He/WT-F	AGATAGGAGAGGTTGGTGCCCTAT
	30740.1-R	AAAATCCTGCCACAAGCAAATGC

Suppl. Table 4: The Key Resources Table in This Study

REAGENT or RESOURCE	SOURCE	IDENTIFIER
**Antibodies**		
**Flow Cytometry Assays**		
Alexa Fluor® 488 Rat Anti-Mouse IL-6 (MP5-20F3)	BD Biosciences	Cat# 561363; RRID: AB_10694253
Alexa Fluor® 647 Rat Anti-Mouse CD14 (rmC5-3)	BD Biosciences	Cat# 565743; RRID: AB_2739340
Alexa Fluor® 647 Rat Anti-Mouse CD206 (MR5D3)	BD Biosciences	Cat# 565250; RRID: AB_2739133
APC-Cy™7 Mouse Anti-Human CD16 (3G8)	BD Biosciences	Cat# 557758; RRID: AB_396864
APC-Cy™7 Mouse Anti-Human CD3 (SK7)	BD Biosciences	Cat# 557832; RRID: AB_396890
APC-Cy™7 Rat Anti-CD11b (M1/70)	BD Biosciences	Cat# 557657; RRID: AB_396772
APC-R700 Mouse Anti-Human IL-17A (N49-653)	BD Biosciences	Cat# 565163; RRID: AB_2739087
BB515 Mouse Anti-Human CD4 (RPA-T4)	BD Biosciences	Cat# 564419; RRID: AB_2744419
BB700 Hamster Anti-Mouse CD11C (HL3)	BD Biosciences	Cat# 566505; RRID: AB_2869773
BB700 Rat Anti-Mouse CD197 (CCR7) (4B12)	BD Biosciences	Cat# 566462; RRID: AB_2744307
BB700 Rat Anti-Mouse TNF (MP6-XT22)	BD Biosciences	Cat# 566511; RRID: AB_2869775
BUV661 Mouse Anti- Human HLA-DR (G46-6)	BD Biosciences	Cat# 612980
BV421 Mouse Anti-Human RORγt (Q21-559)	BD Biosciences	Cat# 563282; RRID: AB_2738114
BV421 Rat Anti-Human and Viral IL-10 (JES3-9D7)	BD Biosciences	Cat# 564053; RRID: AB_2738566
BV421 Rat Anti-Mouse CX3CR1 (Z8-50)	BD Biosciences	Cat# 567531
BV480 Rat Anti-Mouse F4/80 (T45-2342)	BD Biosciences	Cat# 565635; RRID: AB_2739313
BV510 Mouse Anti-Human CD14 (MφP9)	BD Biosciences	Cat# 563079; RRID: AB_2737993
BV510 Mouse Anti-Human IFN-γ (B27)	BD Biosciences	Cat# 563287; RRID: AB_2738118
BV605 Mouse Anti-Human CD206 (19.2)	BD Biosciences	Cat# 740417; RRID: AB_2740147
BV605 Mouse Anti-Human CD25 (2A3)	BD Biosciences	Cat# 562660; RRID: AB_2744343
BV605 Rat Anti-Mouse CD192 (CCR2) (475301)	BD Biosciences	Cat# 747969; RRID: AB_2872430
BV650 Mouse Anti-Human CD11c (B-ly6)	BD Biosciences	Cat# 563404; RRID: AB_2732048
FITC Mouse Anti-HTNV NP (1A8)	Prepared by our Lab	N/A
FITC Rat Anti-Mouse IL-10 (JES5-16E3)	BD Biosciences	Cat# 554466; RRID: AB_395411
FITC Rat Anti-Mouse Ly-6C (AL-21)	BD Biosciences	Cat# 561085; RRID: AB_394628
FITC Rat Anti-Mouse TNF (MP6-XT22)	BD Biosciences	Cat# 561064; RRID: AB_395379
FITC Mouse Anti-Human CD11b (ICRF44)	BD Biosciences	Cat# 562793; RRID: AB_1645544
PE Hamster Anti-Mouse CD80 (16-10A1)	BD Biosciences	Cat# 561955; RRID: AB_395039
PE Mouse anti-Human FoxP3 (236A/E7)	BD Biosciences	Cat# 560852; RRID: AB_10563418
PE Mouse Anti-Human IL-8 (G265-8)	BD Biosciences	Cat# 554720; RRID: AB_395529
PE Rat Anti-Mouse CD86 (GL1)	BD Biosciences	Cat# 561963; RRID: AB_10896971
PE Rat Anti-Mouse F4/80 (T45-2342)	BD Biosciences	Cat# 565410; RRID: AB_2687527
PE Rat Anti-Mouse IL-12 (p40/p70) (C15.6)	BD Biosciences	Cat# 554479; RRID: AB_395420
PE Rat Anti-mouse iNOS (CXNFT)	Thermo Fisher	Cat# 12-5920-82; RRID: AB_2572642
PE-Cy™7 Mouse Anti-GATA3 (L50-823)	BD Biosciences	Cat# 560405; RRID: AB_1645544
PerCP-Cy™5.5 Mouse Anti-Human TNF (MAb11)	BD Biosciences	Cat# 560679; RRID: AB_1727579
PerCP-Cy™5.5 Mouse Anti-T-bet (O4-46)	BD Biosciences	Cat# 561316; RRID: AB_10611726
**Immunoblot & Immunofluorescent Measurements**		
Anti-NF-κB p65 Antibody	Abcam	Cat# ab16502; RRID: AB_443394

Anti-activated Notch1 Antibody (NICD)	Abcam	Cat# ab8925; RRID: AB_306863
Anti-CD34 Antibody [EP373Y]	Abcam	Cat# ab81289; RRID: AB_1640331
Anti-DDDDK Tag (Binds to FLAG® tag sequence) Antibody [F-tag-01]	Abcam	Cat# ab18230; RRID: AB_444336
Anti-ERK1+ERK2 (phospho T202 + Y204) Antibody [ERK12T202Y204-A11]	Abcam	Cat# ab278538
Anti-ERK1+ERK2 Antibody [EPR17526]	Abcam	Cat# ab184699; RRID: AB_2802136
Anti-F4/80 Antibody [CI: A3-1]	Abcam	Cat# ab6640; RRID: AB_1140040
Anti-GAPDH Antibody [6C5]	Abcam	Cat# ab8245; RRID: AB_2107448
Anti-GFP Antibody	Abcam	Cat# ab290; RRID: AB_303395
Anti-HA Tag Antibody	Abcam	Cat# ab9110; RRID: AB_307019
Anti-IKKα+IKKβ (phospho S180+S181) Antibody	Abcam	Cat# ab55341; RRID: AB_883038
Anti-IKKα+IKKβ Antibody [EPR16628]	Abcam	Cat# ab178870
Anti-iNOS Antibody [EPR16635]	Abcam	Cat# ab210823; RRID: AB_2861417
Anti-IRF4 Antibody	Santa Cruz Biotechnology	Cat# sc-48338; RRID: AB_627828
Anti-IRF5 Antibody [EPR17067]	Abcam	Cat# ab181553; RRID: AB_2801301
Anti-IκB α (phosphoS36) Antibody [EPR6235(2)]	Abcam	Cat# ab133462; RRID: AB_2801653
Anti-IκBα (phospho S32) Antibody [EPR3148]	Abcam	Cat# ab92700; RRID: AB_10562951
Anti-IκBα Antibody [E130]	Abcam	Cat# ab32518; RRID: AB_733068
Anti-Jagged1 Antibody	Abcam	Cat# ab7771; RRID: AB_2280547
Anti-Jagged2 Antibody [EPR3646]	Abcam	Cat# ab226814
Anti-JNK1 (phospho T183/Y185) Antibody [EPR20763]	Abcam	Cat# ab215208
Anti-JNK1 Antibody [EPR17557]	Abcam	Cat# ab199380
Anti-Lamin B1 Antibody	Abcam	Cat# ab16048; RRID: AB_443298
Anti-Myc Tag Antibody [9E10]	Abcam	Cat# ab32; RRID: AB_303599
Anti-NF-κB p65 (phospho S276) Antibody	Abcam	Cat# ab194726
Anti-NF-κB p65 (phospho S468) Antibody	Abcam	Cat# ab31473; RRID: AB_881299
Anti-NF-κB p65 (phospho S529) Antibody	Abcam	Cat# ab97726; RRID: AB_10681170
Anti-NF-κB p65 (phospho S536) Antibody	Abcam	Cat# ab86299; RRID: AB_1925243
Anti-Notch1 Antibody [EP1238Y]	Abcam	Cat# ab52627; RRID: AB_881725
Anti-Notch2 Antibody	Abcam	Cat# ab137665
Anti-Notch3 Antibody	Abcam	Cat# ab23426; RRID: AB_776841
Anti-STAT1 (phospho S727) Antibody [EPR3146]	Abcam	Cat# ab109461; RRID: AB_10863745
Anti-STAT1 (phospho Y701) Antibody	Abcam	Cat# ab30645; RRID: AB_779082
Anti-STAT1 Antibody	Abcam	Cat# ab47425; RRID: AB_882708
Anti-STAT3 (phospho S727) Antibody [E121-31]	Abcam	Cat# ab32143; RRID: AB_2286742
Anti-STAT3 (phospho Y705) Antibody [EPR23968-52]	Abcam	Cat# ab267373
Anti-STAT3 Antibody [EPR787Y]	Abcam	Cat# ab68153; RRID: AB_2889877
Anti-Tubulin Antibody	Abcam	Cat# ab6046; RRID: AB_2210370
Donkey Anti-Goat IgG H&L (Cy3 ®)	Abcam	Cat# ab6949; RRID: AB_955018
FITC Anti-NF-κB p65 (phospho S536) Antibody [NFKBp65S536-B7]	Abcam	Cat# ab278631
Goat Anti-Mouse IgG H&L (Cy3 ®)	Abcam	Cat# ab97035; RRID: AB_10680176
Goat Anti-Mouse IgG H&L (Cy5 ®)	Abcam	Cat# ab6563; RRID: AB_955068

Goat Anti-Rabbit IgG H&L (Cy3 ®)	Abcam	Cat# ab6939; RRID: AB_955021
Goat Anti-Rabbit IgG H&L (Cy5 ®)	Abcam	Cat# ab6564; RRID: AB_955061
Human/Mouse/Rat RelA/NF κB p65 Antibody	R&D Systems	Cat# AF5078; RRID: AB_2179033
IRDye® 680RD Goat Anti-Mouse IgG (H + L)	LI-COR	Cat# 925-68070; RRID: AB_2651128
IRDye® 800CW Goat Anti-Rabbit IgG (H + L)	LI-COR	Cat #926-32211; RRID: AB_621843
Mouse monoclonal Anti-HTNV Gn (Gn-1)	Prepared by our Lab	N/A
Mouse monoclonal Anti-HTNV NP (1A8)	Prepared by our Lab	N/A
Mouse/Rat Notch1 Antibody	R&D Systems	Cat# AF1057; RRID: AB_2153372
Phospho-NF-κB p65/RelA-S276 Rabbit pAb	ABclonal	Cat# AP0123; RRID: AB_2771505
Phospho-NF-κB p65/RelA-S468 Rabbit pAb	ABclonal	Cat# AP0446; RRID: AB_2771508
Phospho-NF-κB p65/RelA-S529 Rabbit pAb	ABclonal	Cat# AP0944; RRID: AB_2863855
Phospho-NF-κB p65/RelA-S536 Rabbit pAb	ABclonal	Cat# AP0475; RRID: AB_2771511
Rabbit Anti-Rat IgG H&L (FITC)	Abcam	Cat# ab6730; RRID: AB_955327
**Neutralizing & ELISA Experiments**		
Anti-TNF alpha Antibody [2C8]	Abcam	Cat# ab8348; RRID: AB_306503
Anti-Flavivirus Group Antigen [D1-4G2-4-15 (4G2)]	Absolute Antibody	Cat# Ab00230-2.0; RRID: AB_2715504
HRP-labelled 1A8 for NP Detection by ELISA	Prepared by our Lab	N/A
Mouse Monoclonal Anti-HTNV GP (3D8)	Prepared by our Lab	N/A
TNF-alpha/TNFA/TNFSF2 Neutralizing Antibody	SinoBiological	Cat# 50349-RN023
**Strains**		
Hantaan Virus (HTNV, 76-118)	Conserved in our lab	N/A
Dengue Virus 2 (DENV2)	Conserved in our lab	N/A
DH5α	TransGen Biotech	Cat# CD201
Enterovirus 71 (EV71)	Conserved in our lab	N/A
Herpes Simplex Type 2 (HSV-2)	Conserved in our lab	N/A
Sendai Virus (SeV)	Conserved in our lab	N/A
Vesicular Stomatitis Virus (VSV)	Conserved in our lab	N/A
**Critical Commercial Assays**		
Bio-Plex Calibration Kit	Bio-Rad	Cat# 171203060
Bio-Plex Human 40-plex	Bio-Rad	Cat# 171AK99MR2
Bio-Plex Validation Kit	Bio-Rad	Cat# 171203001
BrdU Immunohistochemistry Kit	Abcam	Cat# ab125306
Co-immunoprecipitation Kit	Proteintech	Cat# KIP-1
Compat-Able™ BCA Protein Assay Kit	Thermo Fisher	Cat# 21063
DCFDA/H2DCFDA - Cellular ROS Assay Kit	Abcam	Cat# ab113851
Diagnostic Kit for IgG Antibody to Hantaviruses (ELSIA)	WAITAI BioPharm	Cat# YZB/Guo 3760-2014
Dual-Luciferase Assay Kit	Promega	Cat# TM040
EasySep™ Human Monocyte Isolation Kit	StemCell	Cat# 19359
Fixation/Permeablization Kit	BD Biosciences	Cat# 554714
Fluorescent In Situ Hybridization Kit	Ribo Biotechnology	Cat# C10910
H&E staining kit	Servicebio	Cat# G1005
Human Interferon alpha 1 ELISA Kit	Abcam	Cat# ab213479

Human TNF alpha ELISA Kit	Abcam	Cat# ab181421
Magna RIP™	Millipore Sigma	Cat# 17-700
Mouse IL-1 beta/IL-1F2 Quantikine ELISA Kit	R&D Systems	Cat# MLB00C
Mouse IL-10 ELISA Kit	Abcam	Cat# ab108870
Mouse IL-6 Quantikine ELISA Kit	R&D Systems	Cat# M6000B
Mouse Interferon alpha 1 ELISA Kit	Abcam	Cat# ab252352
Mouse IP-10 ELISA Kit (CXCL10)	Abcam	Cat# ab214563
Mouse MCP1 ELISA Kit (CCL2)	Abcam	Cat# ab208979
Mouse TNF alpha ELISA Kit	Abcam	Cat# ab208348
Ms Ig Kpa Comp Bead Set	BD Biosciences	Cat# 552843
Neon™ Transfection System Kit	Invitrogen	Cat# MPK10096
NE-PER Nuclear and Cytoplasmic Extraction Reagents	Thermo Fisher	Cat# 78833
NF-κB p65 Transcription Factor Assay Kit	Abcam	Cat# ab133112
NorthernMax™ Kit	Invitrogen	Cat# AM1940
PrimeScript™ RT Reagent Kit (Perfect Real Time)	TaKaRa	Cat# RR037B
Rat Ig Kpa Comp Bead Set	BD Biosciences	Cat# 552844
RNA-Binding Protein Immunoprecipitation Kit		
RNAscope 3-plex Negative Control Probes	ACD Bio	Cat# 320871
RNAscope Fluorescent Multiplex Reagent Kit	ACD Bio	Cat# 320850
RNAscope Probe Diluent	ACD Bio	Cat# 300041
SDS-PAGE Gel kit	CW Bio	Cat# CW0022S
SYBR Premix EX Taq™ (Perfect Real Time)	TaKaRa	Cat# RR420A
Total RNA Extraction Kit	TIANGEN Biotech	Cat# DP419
Transcription Factor Buffer Set	BD Biosciences	Cat# 562574
**Chemicals, Peptides, and Recombinant Proteins**		
2-Deoxy-D-glucose (2-DG)	Selleck	Cat# S4701
4’,6-diamidino-2-phenylindole (DAPI)	Thermo Fisher	Cat# D9542
Alcohol	Sinopharm	Cat# 100092683
Ammonia solution	Servicebio	Cat# G1005-4
Anti-Flag magnetic beads	Bimake	Cat# B26102
Anti-HA magnetic beads	Bimake	Cat# B26202
Anti-Myc magnetic beads	Bimake	Cat# B26302
Antimycin A	Sigma-Aldrich	Cat# A8674-25MG
Bovine Serum Albumin (BSA)	Sigma-Aldrich	Cat# A1933
Brilliant Stain Buffer	BD Biosciences	Cat# 563794
Carbonyl cyanide 4-(trifluoromethoxy) phenylhydrazone (FCCP)	Sigma-Aldrich	Cat# C2920-10MG
Clophosome®, Clodronate Liposomes (Neutral)	FormuMax	Cat# F70101C-N-10
cOmplete™, Mini Protease Inhibitor Cocktail	Sigma-Aldrich	Cat# 4693124001
Antisense LNA™ GapmeR Standard for lncRNA MSTRG-22387.1	QIAGEN	Cat# 339511 LG00239397-DDA
Antisense LNA™ GapmeR Standard for lncRNA MSTRG-30740.1-1	QIAGEN	Cat# 339511 LG00239366-DDA
Antisense LNA™ GapmeR Standard for lncRNA MSTRG-30740.1-2	QIAGEN	Cat# 339511 LG00239367-DDA

Antisense LNA™ GapmeR Standard for lncRNA MSTRG-30740.1-3	QIAGEN	Cat# 339511 LG00239368-DDA
Antisense LNA™ GapmeR Standard for lncRNA MSTRG-30740.1-4	QIAGEN	Cat# 339511 LG00239369-DDA
Antisense LNA™ GapmeR Standard for lncRNA MSTRG-30740.1-10	QIAGEN	Cat# 339511 LG0023975-DDA
Antisense LNA™ GapmeR Standard for lncRNA MSTRG-30928.1-1	QIAGEN	Cat# 339511 LG00239418-DDA
Antisense LNA™ GapmeR Standard for lncRNA MSTRG-30928.1-2	QIAGEN	Cat# 339511 LG00239419-DDA
Custom LNA™ Detection Probes for lncRNA MSTRG-22387.1 (for Northern Blot)	QIAGEN	Cat# 339500 LCD0168369-BKJ
Custom LNA™ Detection Probes for lncRNA MSTRG-30740.1 (for Northern Blot)	QIAGEN	Cat# 339500 LCD0168366-BKJ
Custom LNA™ Detection Probes for lncRNA MSTRG-30928.1 (for Northern Blot)	QIAGEN	Cat# 339500 LCD0168372-BKJ
Custom LNA™ Detection Control Probes (for GAPDH)	QIAGEN	Cat# 339508 LCD0000001-BDJ
Custom lncRNA FISH Probe 1 for MSTRG-30740-1	Ribo Biotechnology	N/A
Custom lncRNA FISH Probe 2 for MSTRG-30740-1	Ribo Biotechnology	N/A
Custom lncRNA FISH Probe Mix for MSTRG-22387-1	Ribo Biotechnology	N/A
Custom lncRNA FISH Probe Mix for MSTRG-30740-1	Ribo Biotechnology	N/A
Custom lncRNA FISH Probe Mix for MSTRG-30928-1	Ribo Biotechnology	N/A
DAPT (GSI-IX, LY-374973)	Selleck	Cat# S2215
Differentiating solution	Servicebio	Cat# G1005-3
Dimethyl sulfoxide (DMSO)	Invitrogen	Cat# D12345
DLL1 Protein, Mouse, Recombinant (His Tag)	SinoBiological	Cat# 50522-M08H
Ethylenediamine Tetraacetic Acid (EDTA)	Sigma-Aldrich	Cat# E5134
FAM labeled RNAs	GenePharma	Cat# A07001
Glucose	Agilent	Cat# 103577-100
GM-CSF, recombinant, murine	PeproTech	Cat# 315-03
Jagged 1 Protein, Human, Recombinant (His Tag)	SinoBiological	Cat# 11648-H08H
JetPEI reagents	PolyPlus	Cat# 101-40N
Lipofectamine 2000	Invitrogen	Cat# 1858793
Lipopolysaccharide (LPS) (from E. Coli 0111: B4)	InvivoGen	Cat# tlrl-3pelps
m-18S FISH Probe Mix (Red,20T, for mouse)	Ribo Biotechnology	Cat# lnc110104
M-CSF, recombinant, human	PeproTech	Cat# 300-25
M-CSF, recombinant, murine	PeproTech	Cat# 315-02
Neomycin	Sigma-Aldrich	Cat# N6386
Oligomycin A	Sigma-Aldrich	Cat# 75351-5MG
Paraformaldehyde	Sigma-Aldrich	Cat# P6148
Penicillin-Streptomycin	Sigma-Aldrich	Cat# P4333
Phosphatase Inhibitor Cocktail 2	Sigma-Aldrich	Cat# P5726
PMA	Sigma-Aldrich	Cat# P1585
Polyinosinic-polycytidylic acid (Poly(I:C))	InvivoGen	Cat# tlrl-pic
Prolong ® Probe - V-HTNV-S-C2	ACD Bio	Cat# 588541-C2

Prolong™ Gold Antifade Mountant	Thermo Fisher	Cat# P36930
Purified NA/LE Human BD Fc Block™	BD Biosciences	Cat# 564765
Purified Rat Anti-Mouse CD16/CD32 (Mouse BD Fc Block™)	BD Biosciences	Cat# 553141
Puromycin	Sigma-Aldrich	Cat# P9620
RBC lysis buffer (1x)	Gibco	Cat# 21875-034
Resin	Sinopharm	Cat# 10004160
Restore™ Western Blot Stripping Buffer	Thermo Fisher	Cat# 21063
RIPA Lysis Buffer (10x)	Sigma-Aldrich	Cat# 20-188
RNAscope® Probe -Mm-MSTRG-30740-C3	ACD Bio	Cat# 588571
Rotenone	Sigma-Aldrich	Cat# R8875-1G
Seahorse XF DMEM (base) media	Agilent	Cat# 103575-100
Stain Buffer	BD Biosciences	Cat# 554657
TMB ELISA Substrate (High Sensitivity)	Abcam	Cat# ab171523
Triton X-100	Sigma-Aldrich	Cat# 93418
TRIzol reagent	Invitrogen	Cat# 15596-018
Trypsin Digestion Solution, 0.25% (without phenol red)	Solarbio	Cat# T1350
Trypsin-EDTA Solution,0.25% (with phenol red)	Solarbio	Cat# T1320
TSA® Plus Cy3	Akoya Biosciences	Cat# NEL744E001KT
TSA® Plus Cy5	Akoya Biosciences	Cat# NEL745E001KT
Type IV Collagenase	Sigma-Aldrich	Cat# C4-BIOC
Xylene	Sinopharm	Cat# 10023418
**Recombinant DNA**		
pcDNA3.1-S (NP)	Constructed by our Lab	N/A
pcDNA3.1-M (GP)	Constructed by our Lab	N/A
pcDNA3.1-R218H	Hongyan Qin and Hua Han Lab	N/A
pcDNA3.1-Flag/HA-NICD	Constructed by our Lab	N/A
pcDNA3.1-Myc-IKKβ	Constructed by our Lab	N/A
pcDNA3.1-Myc-IκBα	Constructed by our Lab	N/A
pcDNA3.1-GFP/Myc-p65	Constructed by our Lab	N/A
pcDNA3.1-RFP-IκBα	Constructed by our Lab	N/A
pcDNA3.1-Stat1/IRF5/Stat3/IRF4	Constructed by our Lab	N/A
pcDNA3.1- MSTRG.22387.1	Constructed by our Lab	N/A
pcDNA3.1- MSTRG.30740.1	Constructed by our Lab	N/A
pcDNA3.1- MSTRG.30928.1	Constructed by our Lab	N/A
pNF-κB-luc	Beyotime	Cat# vD2206-1μg
PsPAX2 and pMD2.G (Lentivirus System)	Conserved in our Lab	N/A
pLVX-Lnc-ip65-ZsGreen1 (Lenti-lnc-ip65)	Constructed by our Lab	N/A
pFastBac™ Dual-S	Constructed by our Lab	N/A
Renilla Plasmid (pRL-TK)	Conserved in our lab	N/A
Truncated NICD/ p65/ IKKβ Plasmids	Constructed by our Lab	N/A
Truncated Lnc-ip65 Plasmids	Constructed by our Lab	N/A
**Experimental Models: Cell Lines & Transgenic Mice**		

**Cell Lines**		
bEnd.3	Procell	Cat# CL-0598
HEK293	Procell	Cat# CL-0001
HUVEC	Procell	Cat# CL-0122
MH-S	Procell	Cat# CL-0597
NIH/3T3	Procell	Cat# CL-0171
RAW264.7	Procell	Cat# CL-0190
THP-1	Procell	Cat# CL-0233
Vero E6	ATCC	Cat# CRL-1586
**Transgenic Mice**		
C57BL/6J	The Jackson Laboratory	JAX stock 000664
IFNAR1 Deficient (IFNAR1$^{-/-}$) C57BL/6J Mice	The Jackson Laboratory	JAX stock 010830
Lnc-ip65 Deficient (lnc-ip65$^{-/-}$) C57BL/6J Mice	Constructed by our Lab	N/A
NICD$^{STOP\text{-}floxed}$ (Lyz2-Cre^{+}) C57BL/6J Mice	Hongyan Qin and Hua Han Lab (Department of Genetics and Developmental Biology, AFMU)	N/A
RBP-J^{CKO} (Lyz2-Cre^{+} RBP-J^{floxed}) C57BL/6J Mice	Hongyan Qin and Hua Han Lab	N/A
RIG-I Deficient (RIG-I$^{-/-}$) C57BL/6J Mice	Experimental Animal Center of AFMU	N/A
**Oligonucleotides**		
RNAi Sequences, See **Suppl. Table 2**	This Paper	N/A
PCR Primer Sequences, See **Suppl. Table 3**	This Paper	N/A
**Software and Algorithms**		
Adobe Illustrator CC 2018	Adobe	https://www.adobe.com/
Adobe Photoshop CC 2018	Adobe	https://www.adobe.com/
Bio-Plex Manager software, Version 6.0	Bio-Rad	https://www.bio-rad.com/
CaseViewer	3DHISTECH	https://www.3dhistech.com/
FlowJo v10	TreeStar	https://www.flowjo.com/
GraphPad Prism 8	GraphPad Software	https://www.graphpad.com/
Hitachi TEM system	Hitachi	https://www.hitachi-hightech.com/us
ImageJ v1.50	ImageJ	https://www.imagej.nih.gov/ij/
Image Studio™ Lite Software	Odyssey	https://licor.com/bio/image-studio-lite/
LightCycler® 96 Application Software	Roche	https://www.roche-applied-science.com
R v.3.6.2	R-project	https://www.r-project.org/
Seahorse Wave Desktop Software, Version 2.6	Agilent	https://www.agilent.com.cn/
