# Supplementary material for: Disparate macrophage responses are linked to infection outcome of Hantan virus in humans or rodents
Source: Nat Commun. 2024 Jan 10;15:438. doi: 10.1038/s41467-024-44687-4 (PMC10781751; doi:10.1038/s41467-024-44687-4)
Supplement: Supplementary file 3 — Description of Additional Supplementary Files [file 41467_2024_44687_MOESM3_ESM.pdf]

Description of Additional Supplementary Files

Supplementary Data 1

Sequences of Murine-specific LncRNAs Downstream Notch pathway, Related to Fig.6

Supplementary Data 2

Protein Binding Potential of Murine-specific LncRNAs, Related to Fig.6
