# Supplementary material for: Disparate macrophage responses are linked to infection outcome of Hantan virus in humans or rodents
Source: Nat Commun. 2024 Jan 10;15:438. doi: 10.1038/s41467-024-44687-4 (PMC10781751; doi:10.1038/s41467-024-44687-4)
Supplement: Supplementary file 6 — Reporting Summary [file 41467_2024_44687_MOESM6_ESM.pdf]

nature portfolio

Corresponding author(s): Fanglin Zhang

Last updated by author(s): Dec 7, 2023

Reporting Summary

Nature Portfolio wishes to improve the reproducibility of the work that we publish. This form provides structure for consistency and transparency in reporting. For further information on Nature Portfolio policies, see our Editorial Policies and the Editorial Policy Checklist.

Statistics

For all statistical analyses, confirm that the following items are present in the figure legend, table legend, main text, or Methods section.

n/a	Confirmed	
☐	☒	The exact sample size (n) for each experimental group/condition, given as a discrete number and unit of measurement
☐	☒	A statement on whether measurements were taken from distinct samples or whether the same sample was measured repeatedly
☐	☒	The statistical test(s) used AND whether they are one- or two-sided<br>*Only common tests should be described solely by name; describe more complex techniques in the Methods section.*
☐	☒	A description of all covariates tested
☐	☒	A description of any assumptions or corrections, such as tests of normality and adjustment for multiple comparisons
☐	☒	A full description of the statistical parameters including central tendency (e.g. means) or other basic estimates (e.g. regression coefficient) AND variation (e.g. standard deviation) or associated estimates of uncertainty (e.g. confidence intervals)
☐	☒	For null hypothesis testing, the test statistic (e.g. F, t, r) with confidence intervals, effect sizes, degrees of freedom and P value noted<br>*Give P values as exact values whenever suitable.*
☒	☐	For Bayesian analysis, information on the choice of priors and Markov chain Monte Carlo settings
☒	☐	For hierarchical and complex designs, identification of the appropriate level for tests and full reporting of outcomes
☒	☐	Estimates of effect sizes (e.g. Cohen's d, Pearson's r), indicating how they were calculated

Our web collection on statistics for biologists contains articles on many of the points above.

Software and code

Policy information about availability of computer code

Data collection

The following tools are used for data collection:
(1) BD FACS-Canto II flow cytometer (BD);
(2) Bio-Plex MAGPIX Multiplex Reader (Bio-Rad);
(3) Olympus VS200, FV 3000 (Olympus);
(4) Live Cell Station (A1R-HD25, Nikon);
(5) Odyssey CLx Infrared Imaging System (LI-COR Biosciences);
(6) LightCycler® 96 Application Software (Roche);
(7) Biotek Synergy 2 SL Microplate Reader (Biotek);
(8) Spark Multimode Microplate Reader (TECAN);
(9) XF extracellular flux analyzer (Seahorse Biosciences);
(10) GloMax® 20/20 Luminometer (Promega);
(11) HT7700 transmission electron microscope (Hitachi);
(12) Panoramic MIDI (3DHISTECH).

Data analysis

The following software were used for data analysis:
(1) Image J software (version 1.5) and Image Studio Lite Software (Odyssey) were used for immunoblot analysis;
(2) FlowJo software (version 10) was used for analysis of FACS-Canto II data;
(3) GraphPad Prism (version 8.0) and SPSS (version 26.0) were used for statistical analysis;
(4) R (version 3.6.2),was used for gene set enrichment analysis.

For comparison of two groups, two-tailed unpaired Student's t test is applied. For multiple comparisons, one way ANOVA is performed, followed by Dunnett's multiple comparisons test. Survival analysis is performed with the log-rank [Mantel–Cox] test. Differences are considered statistically significant when the p values were <0.05 (*), <0.01 (**) and <0.001 (***). Statistically nonsignificant data (p value > 0.05) are indicated as NS. Data are presented as the mean ± SEM if not stated otherwise in the figure legends. The exact p values are shown in the figure or figure legends.

For manuscripts utilizing custom algorithms or software that are central to the research but not yet described in published literature, software must be made available to editors and reviewers. We strongly encourage code deposition in a community repository (e.g. GitHub). See the Nature Portfolio guidelines for submitting code & software for further information.

Data

Policy information about availability of data

All manuscripts must include a data availability statement. This statement should provide the following information, where applicable:

- Accession codes, unique identifiers, or web links for publicly available datasets
- A description of any restrictions on data availability
- For clinical datasets or third party data, please ensure that the statement adheres to our policy

The authors declare that the data supporting the findings of this study are available within the manuscript, its supplementary information and the Source Data file。All data are publicly accessible. This study did not generate any unique code, software, or algorithms. RNA-seq data are available online from the ArrayExpress database with the accession number E-MTAB-11353 (for fig.4a) and E-MTAB-11926 (for fig.6a). Source data are provided with this paper.

Human research participants

Policy information about studies involving human research participants and Sex and Gender in Research.

Reporting on sex and gender	All available patient samples were collected irrespective of sex or gender. Healthy controls were collected from both sexes. Sex was determined based on self-reporting.
Population characteristics	Peripheral blood samples and related medical records were collected from two hundred thirty-six hospitalized patients aged from 18 to 35 years old at the Department of Infectious Disease, Tangdu Hospital, from October 2016 to March 2018 (HFRS patients, n=185; Japanese encephalitis patients, the febrile phase, n=15; hepatitis B patients, confirmed chronic infection for more than one-year, inactive phase without liver cirrhosis and antiviral therapy, n=18; hepatitis C patients, confirmed chronic infection for more than one-year, inactive phase without liver cirrhosis and antiviral therapy, n=18). All patients were Han Chinese, and the proportion of males to females nearly equalled 1:1.
Recruitment	All patients visiting our clinics and wiling to participate in the study were included. We excluded HFRS patients with autoimmune diseases, viral hepatitis, haematological diseases, diabetes, cardiovascular diseases, and other kidney or liver diseases. The diagnosis of HFRS or Japanese encephalitis was made based on typical symptoms and signs as well as IgM and IgG antibody positivity against HTNV or JEV in the serum as assessed by ELISA by the Department of Clinical Laboratory, Tangdu Hospital. The diagnosis of chronic HBV or HCV infection was confirmed by viral RNA detection with qRT–PCR. The definition of HFRS phases, classification of disease severity and exclusion criteria were previously described (Yi et al., 2013; Zhang et al., 2015).
Ethics oversight	This study was approved by the Institutional Review Board of Tangdu Hospital (TDLL-2016323). All patients signed the informed consent and agreed to use their samples.

Note that full information on the approval of the study protocol must also be provided in the manuscript.

Field-specific reporting

Please select the one below that is the best fit for your research. If you are not sure, read the appropriate sections before making your selection.

☒ Life sciences ☐ Behavioural & social sciences ☐ Ecological, evolutionary & environmental sciences

For a reference copy of the document with all sections, see nature.com/documents/nr-reporting-summary-flat.pdf

Life sciences study design

All studies must disclose on these points even when the disclosure is negative.

Sample size	This is determined by the software SPSS (power value, more than 0.9), and based on our prior studies and other previous papers with similar experiments. The exact sample size has been displayed in the figures or stated in the figure legend or Method part.
Data exclusions	This has been described in the method part. For the patient sample, we excluded HFRS patients with autoimmune diseases, viral hepatitis, hematological diseases, diabetes, cardiovascular diseases, and other kidney or liver diseases. For the biological experiments, no samples or animals are excluded from analyses.

Replication	This has been descripbed in the figure legends. For the technical replication, each sample was tested three time for the mean value (Flow cytometry assessment).
Randomization	The samples were randomized acquired through table of random numbers.
Blinding	For the human patients, this is a retrospective study for the clinical samples, and no blinding strategy was applied.<br>For the biological experiments, Animals are allocated to their respective group at birth by a blinded investigator. For other experiments, including cell experiments, before performing the corresponding treatment, samples are randomly assigned to control and experimental groups by an investigator blinded to subsequent experimental information using the random number table. The standard laboratory procedures are strictly followed to keeping the experimental environment and facilities consistent and performed under the same conditions. Investigators are blinded to group allocation during data collection, image quantification and data analysis.

Behavioural & social sciences study design

All studies must disclose on these points even when the disclosure is negative.

Study description	N/A
Research sample	N/A
Sampling strategy	N/A
Data collection	N/A
Timing	N/A
Data exclusions	N/A
Non-participation	N/A
Randomization	N/A

Ecological, evolutionary & environmental sciences study design

All studies must disclose on these points even when the disclosure is negative.

Study description	N/A
Research sample	N/A
Sampling strategy	N/A
Data collection	N/A
Timing and spatial scale	N/A
Data exclusions	N/A
Reproducibility	N/A
Randomization	N/A
Blinding	N/A

Did the study involve field work? ☐ Yes ☒ No

Reporting for specific materials, systems and methods

We require information from authors about some types of materials, experimental systems and methods used in many studies. Here, indicate whether each material, system or method listed is relevant to your study. If you are not sure if a list item applies to your research, read the appropriate section before selecting a response.

Materials & experimental systems

n/a	Involved in the study
☐	☒ Antibodies
☐	☒ Eukaryotic cell lines
☒	☐ Palaeontology and archaeology
☐	☒ Animals and other organisms
☒	☐ Clinical data
☒	☐ Dual use research of concern

Methods

n/a	Involved in the study
☒	☐ ChIP-seq
☐	☒ Flow cytometry
☒	☐ MRI-based neuroimaging

Antibodies

Antibodies used

For flow cytometry assays, Alexa Fluor® 488 Rat Anti-Mouse IL-6 (MP5-20F3) (BD Biosciences; Cat# 561363; RRID: AB_10694253); Alexa Fluor® 647 Rat Anti-Mouse CD14 (rmC5-3) (BD Biosciences; Cat# 565743; RRID: AB_2739340); Alexa Fluor® 647 Rat Anti-Mouse CD206 (MR5D3) (BD Biosciences; Cat# 565250; RRID: AB_2739133); APC-Cy™7 Mouse Anti-Human CD16 (3G8) (BD Biosciences; Cat# 557758; RRID: AB_396864); APC-Cy™7 Mouse Anti-Human CD3 (SK7) (BD Biosciences; Cat# 557832; RRID: AB_396890); APC-Cy™7 Rat Anti-CD11b (M1/70) (BD Biosciences; Cat# 557657; RRID: AB_396772); APC-R700 Mouse Anti-Human IL-17A (N49-653) (BD Biosciences; Cat# 565163; RRID: AB_2739087); BB515 Mouse Anti-Human CD4 (RPA-T4) (BD Biosciences; Cat# 564419; RRID: AB_2744419); BB700 Hamster Anti-Mouse CD11C (HL3) (BD Biosciences; Cat# 566505; RRID: AB_2869773); BB700 Rat Anti-Mouse CD197 (CCR7) (4B12) (BD Biosciences; Cat# 566462; RRID: AB_2744307); BB700 Rat Anti-Mouse TNF (MP6-XT22) (BD Biosciences; Cat# 566511; RRID: AB_2869775); BUV661 Mouse Anti- Human HLA-DR (G46-6) (BD Biosciences; Cat# 612980); BV421 Mouse Anti-Human RORγt (Q21-559) (BD Biosciences; Cat# 563282; RRID: AB_2738114); BV421 Rat Anti-Human and Viral IL-10 (JES3-9D7) (BD Biosciences; Cat# 564053; RRID: AB_2738566); BV421 Rat Anti-Mouse CX3CR1 (Z8-50) (BD Biosciences; Cat# 567531); BV480 Rat Anti-Mouse F4/80 (T45-2342) (BD Biosciences; Cat# 565635; RRID: AB_2739313); BV510 Mouse Anti-Human CD14 (MφP9) (BD Biosciences; Cat# 563079; RRID: AB_2737993); BV510 Mouse Anti-Human IFN-γ (B27) (BD Biosciences; Cat# 563287; RRID: AB_2738118); BV605 Mouse Anti-Human CD206 (19.2) (BD Biosciences; Cat# 740417; RRID: AB_2740147); BV605 Mouse Anti-Human CD25 (2A3) (BD Biosciences; Cat# 562660; RRID: AB_2744343); BV605 Rat Anti-Mouse CD192 (CCR2) (475301) (BD Biosciences; Cat# 747969; RRID: AB_2872430); BV650 Mouse Anti-Human CD11c (B-ly6) (BD Biosciences; Cat# 563404; RRID: AB_2732048); FITC Mouse Anti-HTNV NP (1A8) (Prepared by our Lab); FITC Rat Anti-Mouse IL-10 (JES5-16E3) (BD Biosciences; Cat# 554466; RRID: AB_395411); FITC Rat Anti-Mouse Ly-6C (AL-21) (BD Biosciences; Cat# 561085; RRID: AB_394628); FITC Rat Anti-Mouse TNF (MP6-XT22) (BD Biosciences; Cat# 561064; RRID: AB_395379); FITC Mouse Anti-Human CD11b (ICRF44) (BD Biosciences; Cat# 562793; RRID: AB_1645544); PE Hamster Anti-Mouse CD80 (16-10A1) (BD Biosciences; Cat# 561955; RRID: AB_395039); PE Mouse anti-Human FoxP3 (236A/E7) (BD Biosciences; Cat# 560852; RRID: AB_10563418); PE Mouse Anti-Human IL-8 (G265-8) (BD Biosciences; Cat# 554720; RRID: AB_395529); PE Rat Anti-Mouse CD86 (GL1) (BD Biosciences; Cat# 561963; RRID: AB_10896971); PE Rat Anti-Mouse F4/80 (T45-2342) (BD Biosciences; Cat# 565410; RRID: AB_2687527); PE Rat Anti-Mouse IL-12 (p40/p70) (C15.6) (BD Biosciences; Cat# 554479; RRID: AB_395420); PE Rat Anti-mouse iNOS (CXNFT) (Thermo Fisher; Cat# 12-5920-82; RRID: AB_2572642); PE-Cy™7 Mouse Anti-GATA3 (L50-823) (BD Biosciences; Cat# 560405; RRID: AB_1645544); PerCP-Cy™5.5 Mouse Anti-Human TNF (MAb11) (BD Biosciences; Cat# 560679; RRID: AB_1727579); PerCP-Cy™5.5 Mouse Anti-T-bet (O4-46) (BD Biosciences; Cat# 561316; RRID: AB_10611726). For flow cytometry, antibodies are added 1 µl per 106 cells. Respective antibodies are shown in the Suppl. Table 4.

For immunoblot & immunofluorescent measurements, Anti-NF-κB p65 Antibody (Abcam; Cat# ab16502; RRID: AB_443394); Anti-activated Notch1 Antibody (NICD) (Abcam; Cat# ab8925; RRID: AB_306863); Anti-CD34 Antibody [EP373Y] (Abcam; Cat# ab81289; RRID: AB_1640331); Anti-DDDDK Tag (Binds to FLAG® tag sequence) Antibody [F-tag-01] (Abcam; Cat# ab18230; RRID: AB_444336); Anti-ERK1+ERK2 (phospho T202 + Y204) Antibody [ERK12T202Y204-A11] (Abcam; Cat# ab278538); Anti-ERK1+ERK2 Antibody [EPR17526] (Abcam; Cat# ab184699; RRID: AB_2802136); Anti-F4/80 Antibody [CI: A3-1] (Abcam; Cat# ab6640; RRID: AB_1140040); Anti-GAPDH Antibody [6C5] (Abcam; Cat# ab8245; RRID: AB_2107448); Anti-GFP Antibody (Abcam; Cat# ab290; RRID: AB_303395); Anti-HA Tag Antibody (Abcam; Cat# ab9110; RRID: AB_307019); Anti-IKKα+IKKβ (phospho S180+S181) Antibody (Abcam; Cat# ab55341; RRID: AB_883038); Anti-IKKα+IKKβ Antibody [EPR16628] (Abcam; Cat# ab178870); Anti-iNOS Antibody [EPR16635] (Abcam; Cat# ab210823; RRID: AB_2861417); Anti-IRF4 Antibody (Santa Cruz Biotechnology; Cat# sc-48338; RRID: AB_627828); Anti-IRF5 Antibody [EPR17067] (Abcam; Cat# ab181553; RRID: AB_2801301); Anti-IκB α (phosphoS36) Antibody [EPR6235(2)] (Abcam; Cat# ab133462; RRID: AB_2801653); Anti-IκBα (phospho S32) Antibody [EPR3148] (Abcam; Cat# ab92700; RRID: AB_10562951); Anti-IκBα Antibody [E130] (Abcam; Cat# ab32518; RRID: AB_733068); Anti-Jagged1 Antibody (Abcam; Cat# ab7771; RRID: AB_2280547); Anti-Jagged2 Antibody [EPR3646] (Abcam; Cat# ab226814); Anti-JNK1 (phospho T183/Y185) Antibody [EPR20763] (Abcam; Cat# ab215208); Anti-JNK1 Antibody [EPR17557] (Abcam; Cat# ab199380); Anti-Lamin B1 Antibody (Abcam; Cat# ab16048; RRID: AB_443298); Anti-Myc Tag Antibody [9E10] (Abcam; Cat# ab32; RRID: AB_303599); Anti-NF-κB p65 (phospho S276) Antibody (Abcam; Cat# ab194726); Anti-NF-κB p65 (phospho S468) Antibody (Abcam; Cat# ab31473; RRID: AB_881299); Anti-NF-κB p65 (phospho S529) Antibody (Abcam; Cat# ab97726; RRID: AB_10681170); Anti-NF-κB p65 (phospho S536) Antibody (Abcam; Cat# ab86299; RRID: AB_1925243); Anti-Notch1 Antibody [EP1238Y] (Abcam; Cat# ab52627; RRID: AB_881725); Anti-Notch2 Antibody (Abcam; Cat# ab137665); Anti-Notch3 Antibody (Abcam ; Cat# ab23426; RRID: AB_776841); Anti-STAT1 (phospho S727) Antibody [EPR3146] (Abcam; Cat# ab109461; RRID: AB_10863745); Anti-STAT1 (phospho Y701) Antibody (Abcam; Cat# ab30645; RRID: AB_779082); Anti-STAT1 Antibody (Abcam; Cat# ab47425; RRID: AB_882708); Anti-STAT3 (phospho S727) Antibody [E121-31] (Abcam; Cat# ab32143; RRID: AB_2286742); Anti-STAT3 (phospho Y705) Antibody [EPR23968-52] (Abcam; Cat# ab267373); Anti-STAT3 Antibody [EPR787Y] (Abcam; Cat# ab68153; RRID: AB_2889877); Anti-Tubulin Antibody (Abcam; Cat# ab6046; RRID: AB_2210370); Donkey Anti-Goat IgG H&L (Cy3 ®) (Abcam; Cat# ab6949; RRID: AB_955018); FITC Anti-NF-κB p65 (phospho S536) Antibody [NFKBp65S536-B7] (Abcam; Cat# ab278631); Goat Anti-Mouse IgG H&L (Cy3 ®) (Abcam; Cat# ab97035; RRID: AB_10680176); Goat Anti-Mouse IgG H&L (Cy5 ®) (Abcam; Cat# ab6563; RRID: AB_955068); Goat Anti-Rabbit IgG H&L (Cy3 ®) (Abcam; Cat# ab6939; RRID: AB_955021); Goat Anti-Rabbit IgG H&L (Cy5 ®) (Abcam; Cat# ab6564; RRID: AB_955061); Human/Mouse/Rat RelA/NF κB p65 Antibody (R&D Systems; Cat# AF5078; RRID: AB_2179033); IRDye® 680RD Goat Anti-Mouse IgG (H + L) (LI-COR; Cat# 925-68070; RRID: AB_2651128); IRDye® 800CW Goat Anti-Rabbit IgG (H + L) (LI-COR; Cat #926-32211; RRID: AB_621843); Mouse monoclonal Anti-HTNV Gn (Gn-1) (Prepared by our Lab); Mouse monoclonal Anti-HTNV NP (1A8) (Prepared by our Lab); Mouse/Rat Notch1 Antibody (R&D Systems; Cat# AF1057; RRID: AB_2153372); Phospho-NF-κB p65/RelA-S276 Rabbit pAb

	(ABclonal; Cat# AP0123; RRID: AB_2771505); Phospho-NF-κB p65/RelA-S468 Rabbit pAb (ABclonal; Cat# AP0446; RRID: AB_2771508); Phospho-NF-κB p65/RelA-S529 Rabbit pAb (ABclonal; Cat# AP0944; RRID: AB_2863855); Phospho-NF-κB p65/RelA-S536 Rabbit pAb (ABclonal; Cat# AP0475; RRID: AB_2771511); Rabbit Anti-Rat IgG H&L (FITC) (Abcam; Cat# ab6730; RRID: AB_955327). For immunoblot, antibodies are diluted as 1:1000; for immunostaining, antibodies are diluted as 1:200. Respective antibodies are shown in the Suppl. Table 4.
Validation	All the antibodies have been confirmed with immunoblot or immunofluorescent assays, which is marked with the RRID number as shown above. Related data could also be found on the website of associated companies.

Eukaryotic cell lines

Policy information about cell lines and Sex and Gender in Research

Cell line source(s)	THP-1, bEnd.3, NIH/3T3, RAW264.7 and MH-S cells were obtained from Procell (https://www.procell.com.cn/). Vero E6 cells were obtained from ATCC (https://www.atcc.org/).
Authentication	Authentication of the cell line were performed by a Human STR Profiling Cell Authentication Service (Procell or ATCC)
Mycoplasma contamination	Cells tested negative for mycoplasma contamination.
Commonly misidentified lines (See ICLAC register)	No misidentified lines were used in the study.

Animals and other research organisms

Policy information about studies involving animals; ARRIVE guidelines recommended for reporting animal research, and Sex and Gender in Research

Laboratory animals	The C57BL/6J mice (six- to eight-week-old male adult mice weighing 20-22 g or four-day-old neonatal mice) were provided by the Experimental Animal Center of Fourth Military Medical University. All animals were housed in standard cages in a temperature- and humidity-controlled environment on a 12-h light/dark cycle (temperature: 23±1°C; relative humidity: 50–60%) with free access to water. Mice were euthanized by CO2 inhalation at the appropriate time during the study and tissue samples were removed for further experiments.
Wild animals	The epidemiological data of the field mice were collected from the Shaanxi Provincial Notifiable Disease Surveillance System in collaboration with the research team of Pengbo Yu, which was authorized by the government (Tian et al., 2017). For the capture, traps were placed outdoors (set as 4 parallel lines of 25 traps each and spaced intervals of 5 meters). A. agrarius mice were removed from the traps once captured for further investigation. Mice were euthanized by CO2 inhalation at the appropriate time during the study and tissue samples were removed for further experiments. According to the standard measurement for HTNV infection in rodents (Tian and Stenseth, 2019; Tian et al., 2015; Xiao et al., 2018), the lung tissues were acquired for analysis. Mouse samples weighing 22-28 g and without apparent trauma and skin infection were included for analysis. Only male adult A. agrarius mice were used, and the rest were euthanized by CO2 inhalation.
Reporting on sex	Only the male mice were enrolled.
Field-collected samples	No field collected samples were used in the study.
Ethics oversight	The epidemiological data of the field mice were collected from the Shaanxi Provincial Notifiable Disease Surveillance System in collaboration with the research team of Pengbo Yu, which was authorized by the government (Tian et al., 2017)

Note that full information on the approval of the study protocol must also be provided in the manuscript.

Flow Cytometry

Plots

Confirm that:

- [x] The axis labels state the marker and fluorochrome used (e.g. CD4-FITC).
- [x] The axis scales are clearly visible. Include numbers along axes only for bottom left plot of group (a 'group' is an analysis of identical markers).
- [x] All plots are contour plots with outliers or pseudocolor plots.
- [x] A numerical value for number of cells or percentage (with statistics) is provided.

Methodology

Sample preparation	The monocytes or T cells from HFRS patients underwent intracellular cytokine staining immediately, while for the in vitro experiments, the secretion inhibitors are used to precisely measure cytokine production. Generally, FcγII/III receptors of monocytes and macrophages were blocked with anti-CD16/32 antibody (BD Bioscience) before staining the cell surface markers, and brilliant stain buffer (BD Bioscience) was applied prior to staining intracellular cytokines. For the TFs (FoxP3,

RORγt, GATA3 and T-bet) in T cells from healthy or patient PBMCs, the BD Pharmingen™ Transcription Factor Buffer Set was applied. The cells were manipulated in FCM buffer during the FCM assays, which included PBS containing 2% FBS (Gibco) and 2 mM EDTA. For in vitro assays, cells were enzymatically detached with Trypsin-EDTA solution (Solarbio) and subsequently washed and processed with FCM buffer. For the FCM detection of macrophages in spleens, the single-cell suspension of the spleen tissue was acquired through gentle grinding and filtration with a 70 µm cell strainer, and the erythrocytes were lysed with RBC lysis buffer (Gibco).

Instrument	Samples were analyzed with a BD FACSCalibur™ 3-laser flow cytometer or BD FACSCanto 10-laser flow cytometer (cell number=10,0000/ group). Finally, the data were processed with FlowJo v10 (TreeStar).
Software	FlowJo v10 (TreeStar)
Cell population abundance	No cell sorting was performed.
Gating strategy	Single cells were selected by FSC/SSC gates and then FSC/FSC-width, and cells were gated to exclude dead cells, doublets and triplets. The detailed gating strategy for monocytes or T cells were show in Suppl.Fig.1 and Suppl.Fig.2.

☒ Tick this box to confirm that a figure exemplifying the gating strategy is provided in the Supplementary Information.
